# Molecular recognition of pyrazine *N*,*N*′-dioxide using aryl extended calix[4]pyrroles†

**DOI:** 10.1039/d0sc01496f

**Published:** 2020-04-20

**Authors:** Chenxing Guo, Hu Wang, Vincent M. Lynch, Xiaofan Ji, Zachariah A. Page, Jonathan L. Sessler

**Affiliations:** Department of Chemistry, The University of Texas at Austin 105 East 24th Street, Stop A5300 Austin Texas 78712 USA zpage@cm.utexas.edu sessler@cm.utexas.edu; School of Chemistry and Chemical Engineering, Huazhong University of Science and Technology (HUST) Wuhan 430074 China xiaofanji@hust.edu.cn

## Abstract

Calix[4]pyrrole (C4P)-based systems have been extensively explored as binding agents for anions and ion pairs. However, their capacity to act as molecular containers for neutral species remains underexplored. We report here the molecular recognition of pyrazine *N*,*N*′-dioxide (**PZDO**) using a series of aryl extended C4Ps including three α,α-diaryl substituted C4Ps (receptors **1–3**), an α,β-diaryl substituted C4P (receptor **4**) and an α,α,α,α-tetraaryl substituted C4P (receptor **5**). Single crystal structural analyses of the 2 : 1 host–guest complexes between receptors **1–3** and **PZDO** revealed that the C4P subunits exist in an unusual partial cone conformation and that the **PZDO** guest is held within electron-rich cavities formed by the lower rims of the individual C4P macrocycle. In contrast, receptor **5** was seen to adopt the cone conformation in the solid state, allowing one **PZDO** molecule to be accommodated inside the upper-rim cavity. Evidence for guest-directed self-assembly is also seen in the solid state. Evidence for C4P–**PZDO** interactions in CD_3_CN/CD_3_OD solution came from ^1^H NMR spectroscopic titrations. Electrostatic potential maps created by means of density functional theory calculations were constructed. Density functional theory calculations were also performed to analyse the energetics of various limiting binding modes. On the basis of these studies, it is inferred that interactions between the ‘two-wall’ C4P derivatives (*i.e.* receptors **1–4**) and **PZDO** involve a complex binding mode that differs from what has been seen in previous host–guest complexes formed between C4Ps and *N*-oxides. The present study thus paves the way for the further design of C4P-based receptors with novel recognition features.

## Abstract 2

The molecular recognition of pyrazine *N*,*N′*-dioxide by aryl extended 'two-walled' calix[4]pyrrole-based receptors is seen to stabilise two different binding modes in the solid state.

## Graphical abstract

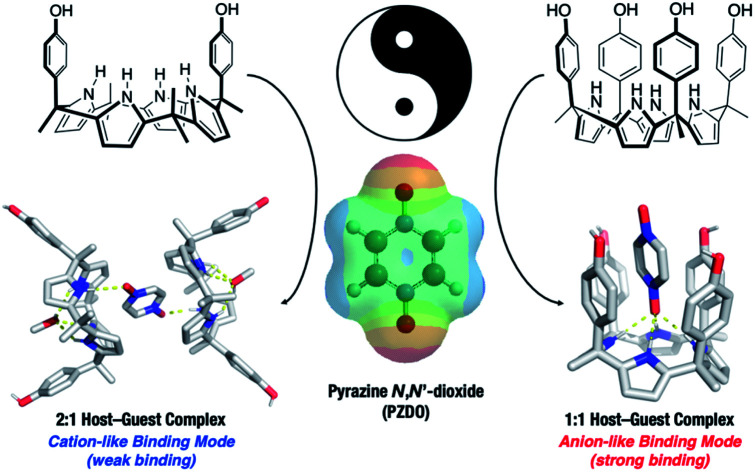

## Introduction

Heterocyclic *N*-oxides have emerged as promising therapeutic drugs or prodrugs thanks to their, *inter alia*, antimicrobial, antitumoral, anti-inflammatory and antiviral activities.^1^ In this context, efforts have been made to synthesise derivatives of quinoxaline 1,4-di-*N*-oxides and phenazine *N*,*N*′-dioxides and study their biological activities.^2,3^ Notably, pyrazine *N*,*N*′-dioxide (**PZDO**) is the redox-active motif embedded in these two types of heterocyclic *N*-oxides and is thought to account for the antitumoral activities towards hypoxic cells. Creating artificial receptors for **PZDO** and its analogues might inform the development of improved supramolecular drug delivery systems and sensors for this class of biologically active species.^4,5^ As a first step towards realising this promise, we report here a series of calix[4]pyrrole-based receptors that allow for the molecular recognition of **PZDO** in CD_3_CN/CD_3_OD solution and in the solid state.

Calix[4]pyrroles (C4Ps) are a class of easy-to-prepare nonaromatic macrocycles that have been extensively studied for their ability to stabilise complexes with anions and ion pairs, particularly in non-competitive solvents.^6–10^ Although an ability to form complexes with simple uncharged solvents was noted early on,^11^ much less effort has been devoted to the recognition of neutral guests using unfunctionalised C4Ps.§§An important class of C4Ps that act as receptors for neutral guests are those bearing fused tetrathiafulvalene (TTF)-subunits. Much of the substrate recognition capability of these systems is ascribed to the presence of the TTF moieties, rather than to the C4Ps *per se*. However, the C4P scaffolds were recognised for providing allosteric control over the recognition processes in question. See: J. S. Park and J. L. Sessler, *Acc. Chem. Res*., 2018, **51**, 2400–2410; S. Bähring, H. D. Root, J. L. Sessler and J. O. Jeppesen, *Org. Biomol. Chem*., 2019, **17**, 2594–2613. In 2009, however, Ballester *et al.* made a seminal contribution to the field by showing that water-soluble C4P derivatives could be used to bind effectively pyridine *N*-oxides (**PNO**s) in aqueous media.^12^ Subsequently, a number of C4P-based receptors and sensors for neutral guests were reported.^13–18^ Ballester and co-workers have also exploited appropriately sized bis-*N*-oxides (*e.g.* 4,4′-bipyridine *N*,*N*′-dioxide) to stabilise C4P dimers or direct the self-assembly of C4P-based capsules.^19–24^ Inspired by these efforts, our group recently prepared a stimuli-responsive supra-amphiphile in water that relies on host–guest interactions between a water-soluble C4P derivative and a tetraphenylethene (TPE)-derived pyridine bis-*N*-oxide.^25^

**Scheme 1:**
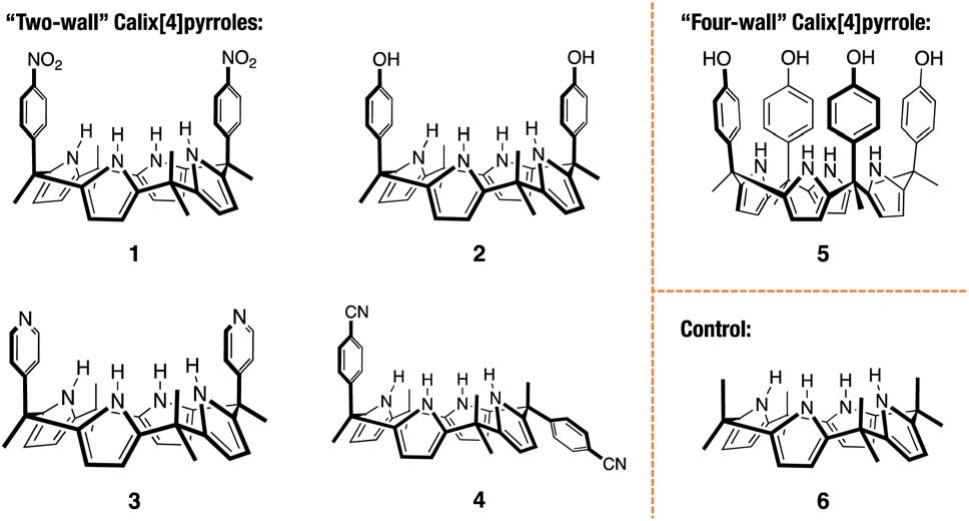
Chemical structures of the calix[4]pyrrole-based receptors **1–6** investigated in this study.

In principle, **PZDO** is one of the smallest aromatic bis-*N*-oxides. To date, **PZDO** and its derivatives have been extensively studied in the context of crystal engineering and have proved to be useful precursors for the fabrication of functional materials.^26–31^ On the other hand, to our knowledge, only one recent study by Mateo-Alonso and co-workers has targeted **PZDO** as a substrate for supramolecular complex formation, wherein the formation of a host–guest complex between an anthracene-linked tetralactam receptor and phenazine *N*,*N*′-dioxides was reported.^32^ As detailed below, we have now prepared several ‘two-wall’ C4P-based molecular receptors (**1–4**) that allow for the encapsulation of **PZDO** in the form of 2 : 1 host–guest complexes in the solid state (Scheme 1).

## Results and discussion

### Preparation and characterisation of the receptors

The *cis*-‘two-wall’ C4Ps **1–3** were prepared in accord with literature methods.^33–35^ The *trans*-‘two-wall’ C4P **4** was prepared *via* a simple two-step synthetic strategy. Briefly, commercially available 4-acetylbenzonitrile was converted to the corresponding dipyrromethane **7** (4-(1,1-di(1*H*-pyrrol-2-yl)ethyl)benzonitrile; *cf.* ESI† for structure) *via* the condensation reaction with pyrrole in the presence of trifluoroacetic acid (TFA). The key intermediate **7** was, in turn, converted to compound **4** by reacting with acetone in the presence of boron trifluoride diethyl etherate (BF_3_·OEt_2_). The desired *trans*-‘two-wall’ C4P **4** was then separated from its *cis*-congener, which also formed under conditions of the condensation, by means of column chromatography. The ‘four-wall’ C4P **5** and control compound **6** were also made according to reported procedures.^36,37^ Compounds **4** and **7** were fully characterised by ^1^H NMR and ^13^C NMR spectroscopies, as well as high-resolution mass spectrometry (HRMS) (see ESI† for details).

### Solid-state complex formation

**Fig. 1:**
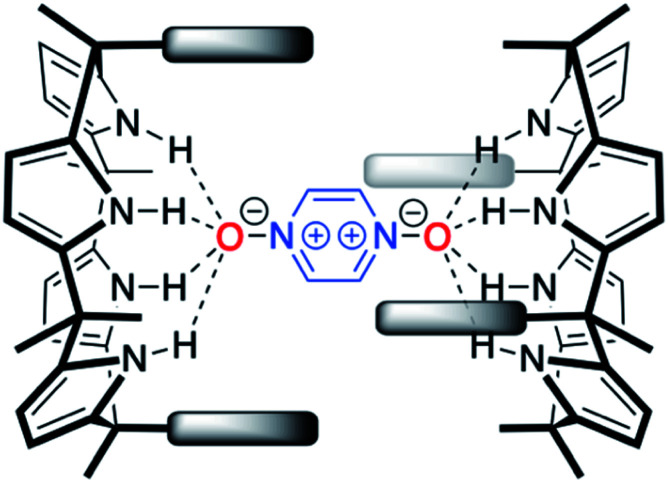
Initially expected binding mode for the 2 : 1 host–guest complex between α,α-*meso*-diaryl substituted C4Ps and **PZDO** in the solid state. The dashed lines indicate the putative convergent hydrogen bonds between the pyrrolic NH protons and the oxygen atoms of **PZDO**. As detailed in the text proper, no evidence for this binding mode was found by either experiment or theory.

With C4Ps **1–6** in hand, efforts turned to studying them as possible receptors for **PZDO**. On the basis of prior studies by Ballester and co-workers involving C4P-based receptors and aromatic bis-*N*-oxides, we envisaged an analogous binding mode would pertain in the case of α,α-diaryl substituted C4Ps and **PZDO** giving rise to the generic structure shown in Fig. 1. Specifically, we expected that two C4P hosts would combine to create a dimer that would encapsulate a single **PZDO** guest within the central cavity. Support for this expectation came from a study by Cafeo *et al.*, wherein a solid-state structure revealed a host–guest complex between the ‘two-wall’ C4P **1** and isophthalate dianion similar to that shown in Fig. 1.^38^ However, as detailed below, a different binding mode was seen when **PZDO** was allowed to crystallise in the presence of **1**.

**Fig. 2:**
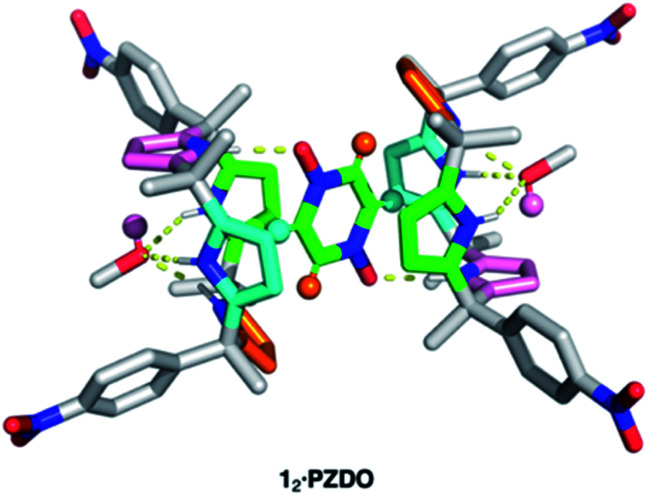
Crystal structure of the 2 : 1 host–guest complex between receptor **1** and **PZDO** with two molecules of methanol being bound to the receptor. Different colours are used to highlight various types of intermolecular non-covalent interactions contributing to the stabilisation of the methanol-solvated **12·PZDO** complex as inferred from the crystal structure. Yellow dashed lines: N–H⋯O hydrogen bonding; orange and cyan: C–H⋯π interactions; pink: O–H⋯π interactions; green: donor–acceptor π–π interactions. Note that non-polar hydrogen atoms of **1** and methanol molecules are removed for clarity.

Single crystals suitable for X-ray diffraction analyses were obtained by allowing **PZDO** to crystallise in the presence of **1**. This was done by dissolving **1** and **PZDO** in a 2 : 1 molar ratio of 1 : 1 chloroform : methanol, and allowing *n*-heptane to diffuse into the resulting solution. The resulting crystal structure is shown in Fig. 2. In contrast to the proposal presented in Fig. 1, each C4P subunit (*i.e.* receptor **1**) was seen to adopt the partial cone conformation rather than the expected cone conformation. Nevertheless, an overall 2 : 1 receptor–substrate complex (**12·PZDO**) is seen with the individual **PZDO** molecules encapsulated inside the cavity defined by the lower rims of the two constituent C4P moieties, albeit offset from the C4P–C4P axis. Methanol molecules are bound to the upper rim of each C4P subunit.

On the basis of the metric parameters, we infer that intermolecular hydrogen bonding interactions serve to stabilise the methanol-solvated **12·PZDO** complex. Specifically, each oxygen atom of **PZDO** is bound to one pyrrolic NH moiety *via* inferred N–H⋯O hydrogen bonds based on the observed N⋯O distance of 2.79 Å. Hydrogen bonds between the methanol oxygen atom and the three other pyrrolic NH moieties are reflected in an average N⋯O distance of 3.08 Å. Evidence for nonclassical hydrogen bonds involving both C–H⋯π and O–H⋯π interactions^39^ came from the fact that each aromatic hydrogen atom of **PZDO** resides in close proximity to an adjacent pyrrole moiety (*ca.* 2.4 Å H-to-centroid). One of the pyrrole rings also lies parallel to the bound **PZDO** at centroid-to-centroid distance of 3.6 Å, indicating possible donor–acceptor π–π interactions between these two moieties.^40^ The net result is an overall binding mode that differs from that seen for the recognition of other *N*-oxide moieties, which are typically accommodated by C4Ps in their cone conformations *via* predominantly pyrrolic NH hydrogen bonding interactions in analogy to what is seen in the case of most C4P anion complexes. In contrast, the binding of **PZDO** to **1** resembles the binding motif seen for small cations in C4P-based ion pair complexes.

**Fig. 3:**
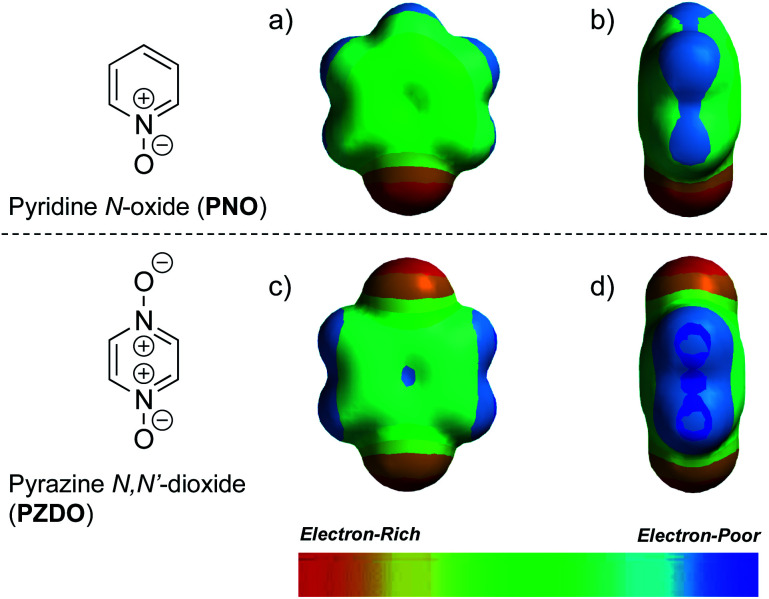
Electrostatic potential (ELPOT) maps showing (a) front and (b) side views of **PNO**, as well as (c) front and (d) side views of **PZDO**. Identical colour scales are used so as to allow for direct comparisons of the associated electrostatic potentials.

To gain insight into the disparate binding behaviour observed for **PNO** and **PZDO**, so-called electrostatic potential (ELPOT) maps of these two ostensibly similar substrates were generated by means of density functional theory (DFT) calculations carried out in the gas phase at the B3LYP/6-31G* level. As shown in Fig. 3, all oxygen atoms on the *N*-oxide moieties of **PNO** and **PZDO** are relatively electron-rich. This is consistent with the finding that these oxygen atoms form hydrogen bonds with the pyrrolic NH protons. On the other hand, the C–H hydrogen atoms of **PZDO**, as well as the associated π-system, were found to be more electron-deficient than those of **PNO**. Presumably, these features allow the hydrogen atoms and π electrons of **PZDO** to stabilise C–H⋯π and donor–acceptor π–π interactions with the pyrrole moieties of **1** more readily than **PNO**, thus facilitating encapsulation of **PZDO** within the lower-rim cavity of **1**.¶¶Support for the notion that the aromatic hydrogen atoms of **PZDO** are relatively electron-deficient and capable of forming nonclassical hydrogen bonds came from the early work by Babu and Nangia involving the study of C–H⋯O hydrogen bonding patterns within two distinct **PZDO** polymorphs. See: N. J. Babu and A. Nangia, *CrystEngComm*, 2007, **9**, 980–983.

**Fig. 4:**
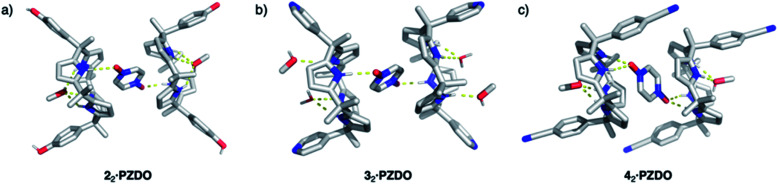
Crystal structures of the 2 : 1 host–guest complexes formed between receptors **2–4** and **PZDO** (shown as (a), (b), and (c), respectively). Presumed intermolecular hydrogen bonds between the hosts and **PZDO** are shown by the yellow dashed lines. Non-polar hydrogen atoms are removed for clarity.

To test the extent to which the C4P might serve to regulate the binding of **PZDO**, studies analogous to the above were carried out using a C4P derivative, *i.e.* receptor **2**, bearing more electron-rich ‘walls’. A single-crystal X-ray diffraction analysis of the resulting complex, **22·PZDO** complex (*cf.*Fig. 4a), revealed a binding mode nearly identical to that seen for **12·PZDO** complex; again, evidence of N–H⋯O hydrogen bonding, C–H⋯π, O–H⋯π and donor–acceptor π–π interactions was seen (see Table S2† for a summary of the bond lengths).

Further support for the notion that it is the substrate, **PZDO**, rather than the electronics of the diaryl ‘walls’ that dictates the binding mode seen for the present ‘two-wall’ C4P receptors came from the X-ray diffraction analysis of single crystals of **32·PZDO** obtained by vapour diffusion of *n*-heptane into the CHCl_3_/CH_3_OH solution containing **3** and **PZDO** (*cf.*Fig. 4b). Again, a non-classical binding mode analogous to what was seen for **12·PZDO** and **22·PZDO** was observed in the solid state (*cf.*Fig. 2 and 4a). However, in the case of **32·PZDO**, one molecule of water and one molecule of methanol, rather than just one molecule of methanol, were found inside the upper-rim cavity, giving rise to a slightly different geometry than that seen in the complexes based on **1** and **2**.

Receptors **1**, **2** and **3** all contain two ‘walls’ that are *cis* to one another. The present study was thus extended to include the *trans*-‘two-wall’ system **4**. In this latter case, analysis of single crystals grown from a CHCl_3_/CH_3_OH solution containing **4** and **PZDO** revealed the formation of a 2 : 1 receptor–substrate complex wherein receptor **4** was found to adopt the 1,2-alternate conformation (*cf.*Fig. 4c). Although unsubstituted C4Ps typically adopt the 1,3-alternate conformation in the absence of guests (*e.g*. anions), several *trans*-‘two-wall’ C4Ps have been reported to adopt the 1,2-alternate conformation in the solid state in the absence of a guest.^33,35,41–43^ These observations led us to suggest that the 1,2-alternate form may be the most stable conformation for *trans*-‘two-wall’ diaryl C4Ps. To the extent this inference is true, we conclude that the interaction with **PZDO** does not induce a conformational change in the case of receptor **4**. Nevertheless, as above, evidence for N–H⋯O hydrogen bonding, C–H⋯π, O–H⋯π and donor–acceptor π–π interactions is seen in the solid state. A short contact between the oxygen atom of **PZDO** and the 4-cyanophenyl moieties present in **4** was also observed, as evidenced by the 2.98 Å separation between the oxygen atom of **PZDO** and the centroid of the phenyl ring.

Taken in concert, the above structural findings lead us to conclude that **PZDO**, in spite of being a neutral species, behaves more like a small cation rather than an anion, in terms of its interaction with ‘two-wall’ diaryl C4Ps. Moreover, cation–π and donor–acceptor π–π interactions, rather than just pyrrolic NH-based hydrogen bonds, appear to control the interactions between the **PZDO** guest and the C4P receptors in the case of complexes **12·PZDO–42·PZDO**.

**Fig. 5:**
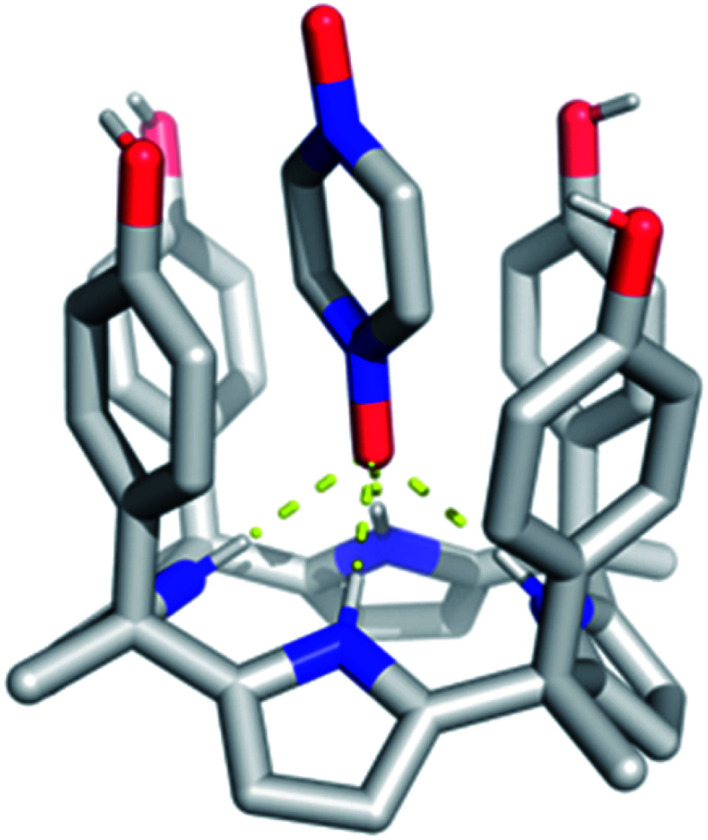
Crystal structure of the 1 : 1 host–guest complex between receptor **5** and **PZDO**. The intermolecular hydrogen bonds between the pyrrolic NH protons and **PZDO** found in the **5·PZDO** complex are shown by yellow dashed lines. Non-polar hydrogen atoms are removed for clarity.

In spite of what was observed with the ‘two-wall’ diaryl C4Ps **1–4**, we wondered if **PZDO** could act as an anion surrogate and bind to C4Ps in a manner analogous to **PNO**. With such considerations in mind, **PZDO** was tested in conjunction with receptor **5**, *i.e.* a so-called ‘four-wall’ C4P derivative bearing electron-rich moieties at the *meso*-positions.^36^ In this case, analysis of single crystals grown from a CHCl_3_/CH_3_OH solution containing **5** and **PZDO** revealed that receptor **5** adopts a cone conformation in the solid state. This choice of conformation allows **PZDO** to be accommodated within the upper-rim cavity *via* four convergent hydrogen bonds between one oxygen atom of **PZDO** and all four pyrrolic NH protons with an average length of 2.97 Å. As the result, a 1 : 1 host–guest complex (*i.e.***5·PZDO**; Fig. 5) was formed in analogy to what was seen in Ballester's seminal study.^12^ In addition, evidence of C–H⋯π and donor–acceptor π–π interactions was also found in the **5·PZDO** complex, as inferred from average bond lengths of 2.44 Å and 3.94 Å, respectively (*cf.* Table S2†).

Unfortunately, attempts to grow diffraction grade single crystals of the putative **PZDO** complex of **6**, an unsubstituted C4P used as a control, using the above procedures returned only mixtures of single crystals of methanol-solvated **6** and **PZDO**.||||The single crystals of **PZDO** obtained in the context of the present study appear to be one of the previously reported polymorphs (CCDC No.: 648204), although with slightly different metric parameters. Data for this crystal structure was thus deposited to the Cambridge Structural Database (CSD) and assigned a CCDC number of 1976061.

### Extended solid-state interactions

Given the interest in **PZDO** as a building block in crystal engineering studies, the packing diagrams of the above systems were considered in an effort to determine whether any guest-mediated self-assembled architectures are stabilised in the solid state.^44,45^ These analyses revealed that whereas only finite ensembles constructed *via* hydrogen bonds were found in the case of **12·PZDO** and **42·PZDO**, infinite ensembles were found in the case of **5·PZDO**, **32·PZDO** and **22·PZDO**. Coincidentally, these structures were found to exist in the form of one-, two- and three-dimensional hydrogen bonding networks, respectively (*vide infra*).

**Fig. 6:**
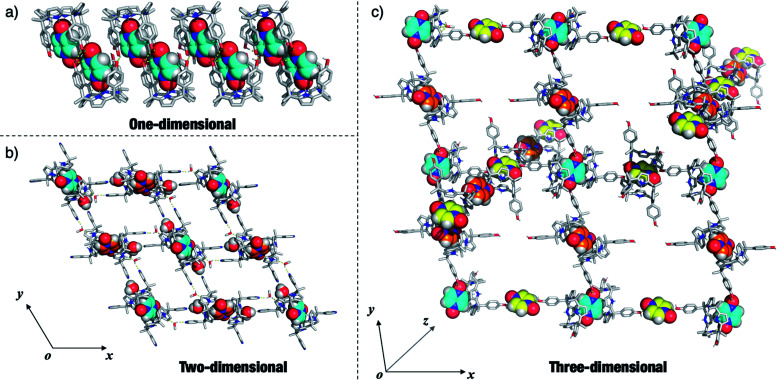
One-, two- and three-dimensional hydrogen bonding networks seen in the crystal structures of (a) **5·PZDO,** (b) **32·PZDO** and (c) **22·PZDO**, respectively. Only the essential solvent molecules serving to define these networks are shown. The **PZDO** and the H_2_O molecules present in the structures in (b) are shown using CPK representations. The carbon atoms of **PZDO** are rendered with different colours to highlight their different spatial orientations. Presumed intermolecular hydrogen bonds between the C4P hosts and **PZDO** are shown by yellow dashed lines. Non-polar hydrogen atoms of the hosts are removed for clarity. Note that as an aid to visualisation, the orientation of the *xyz* coordinate axes shown in the inset to (c) differs from the reported cell axes.

As shown in Fig. 6a, a pair of adjacent **5·PZDO** complexes was seen to constitute a [**5·PZDO**]_2_ dimeric pseudo-capsule, in which the oxygen atom of **PZDO** not directly bound to the pyrrolic NHs forms a presumed intermolecular hydrogen bond with one hydroxyl group of the other host molecule; adjacent [**5·PZDO**]_2_ pseudo-capsules were further linked by two intermolecular hydrogen bonds between the hydroxyl groups. As a result, a formal one-dimensional supramolecular polymer is formed.

In the case of the two-dimensional hydrogen bonding network seen in the extended structure of **32·PZDO** (Fig. 6b), both CH_3_OH and H_2_O act as bridges that link individual 2 : 1 **32·PZDO** complexes *via* presumed hydrogen bonding interactions. Here, the pyridine nitrogen atoms act as hydrogen bond acceptors.

In contrast to receptors **3** and **5**, **22·PZDO** complex is engaged in two distinctive one-dimensional networks that orientated along two of the *xyz* coordination axes as illustrated in Fig. 6c. The **PZDO** molecules, rendered with cyan, yellow and orange, are located in the *xy*, *xz* and *yz* planes, respectively. This complexity is thought to reflect the fact that C4P **2** contains two relatively accessible hydroxyl groups that can act as either hydrogen bond donors or hydrogen bond acceptors. Including interactions with both the bound substrate and the disordered methanol molecules (not shown in Fig. 6c but inferred from the structural analysis), both kinds of limiting hydrogen bonding interactions are seen in the extended crystal structure of **22·PZDO**. Presumably, this multiplicity of interactions plays a key role in stabilising the extended array seen in the solid state.

### Solution-phase analyses

**Fig. 7:**
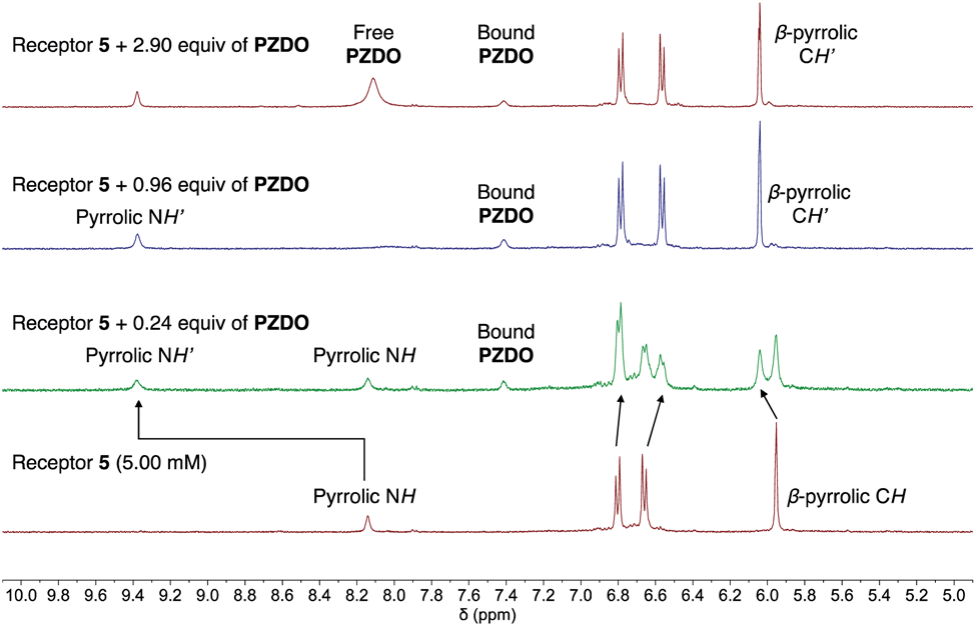
Changes in the ^1^H NMR spectra during the titration of receptor **5** with **PZDO** in CD_3_CN/CD_3_OD (1 : 1 v/v). The arrows indicate how the peaks in the selected region were shifted upon the host–guest complexation.

^1^H NMR spectral titrations were carried out in an effort to quantify the binding interactions between hosts **1–5** and **PZDO** in solution. Again, the unmodified C4P-based receptor **6** was used as a control compound. Since it and several of the other receptors were found to possess low solubilities in the original solvent system used to grow the crystals for the solid-state analyses (*i.e.* CHCl_3_/CH_3_OH), the ^1^H NMR spectral titrations were carried out in an alternative and better solubilising solvent system, namely a 1 : 1 mixture of CD_3_CN/CD_3_OD. The results of these binding studies are summarised in Fig. 7 and S1–S28 (ESI†).

Not surprisingly, perhaps given the presence of four, rather than two, ‘walls’ and the end-on binding seen in the solid state (*vide supra*), receptor **5** was found to possess the highest affinity for **PZDO**. Initial evidence for a strong interaction between **5** and **PZDO** came from the observation of slow exchange kinetics on the NMR time scale. Specifically, as can be seen from an inspection of Fig. 7, the singlet peak assigned to the pyrrolic NH protons of the free receptor at 8.14 ppm gradually disappears upon the addition of increasing quantities of **PZDO**. Concurrently, a new peak at 9.38 ppm assigned to the pyrrolic NH protons of the **PZDO** complex was seen to grow in. The aromatic hydrogens of **PZDO** were found to undergo an upfield shift from 8.14 ppm to 7.41 ppm. Furthermore, after the addition of *ca.* 1.0 equiv. of **PZDO**, no further changes in the chemical shifts were seen other than a continuous increase of the peak at 8.11 ppm ascribed to the aromatic hydrogens of the free **PZDO** guest. These observations were taken as evidence for not only a 1 : 1 binding stoichiometry (as seen in the solid state) but also an association constant (*K*_a_) > 10^4^ M^−1^ for this binding event in this solvent system (CD_3_CN/CD_3_OD 1 : 1 v/v).

The relatively high affinity inferred from the ^1^H NMR spectral titrations was confirmed by means of isothermal titration calorimetry (ITC). As shown in Fig. S23,† the binding isotherm could be fitted to a one-site binding mode, allowing an association constant of (1.53 ± 0.22) × 10^4^ M^−1^ to be determined.

In marked contrast to the slow exchange kinetics seen in the ^1^H NMR spectral titration of **5** with **PZDO**, evidence for fast exchange kinetics was seen when analogous titrations were carried out using receptors **1–4** or **6**. Although not a proof, fast exchange is often associated with weak binding interactions. In addition, although the crystal structures of **12·PZDO–42·PZDO** revealed 2 : 1 binding stoichiometries in the solid state, fitting the binding data to a 2 : 1 model gave poor fits and returned unreasonable stepwise binding constants (*cf.* ESI†). On the other hand, good fits were obtained when the data were fitted to a 1 : 1 binding isotherm. We thus believe that a 1 : 1 interaction dominates in solution. The corresponding mole ratio plots also proved consistent with a 1 : 1 binding mode. The calculated 1 : 1 association constants were found to be (1.4 ± 0.3) × 10^2^ M^−1^, 51 ± 5 M^−1^, 66 ± 7 M^−1^, <10 M^−1^ and 17 ± 0.2 M^−1^ for the host–guest complexes of **1**, **2**, **3**, **4** and **6** with **PZDO**, respectively, in CD_3_CN/CD_3_OD (1 : 1 v/v). Although these values are all low compared to what is seen for most C4P–anion interactions, they do serve to underscore the expectation that it is possible to create modified C4P systems, such as **1–3**, that act as receptors for appropriately chosen neutral substrates and that are more effective in this regard than the parent C4P system, **6**. However, it is also important to note that the electronic variations in the ‘walls’ in these ‘two-wall’ systems do not directly correlate with the binding affinities for **PZDO** complexation. Presumably, this reflects the fact that substrate recognition in solution as in the solid state (*vide supra*) involves the lower rims of the C4P subunits, rather than more classic pyrrolic NH–O^−^ interactions as seen in the case of previous studies involving **PNO**.

In an attempt to elucidate the binding mode between the ‘two-wall’ C4Ps and **PZDO** in solution and per the suggestion of a referee, an effort was made to analyse the complexes by two-dimensional nuclear Överhauser effect spectroscopy (2D NOESY). Specifically, the 2D NOESY spectra of **PZDO** (5.0 mM) were recorded in CD_3_CN/CD_3_OD (1 : 1 v/v) in the presence of 1.0 molar equiv. of receptors **2**, **3** and **6**, respectively. No clear crosspeak between **PZDO** and these receptors was found in the NOESY spectra (*cf.* Fig. S29–S31†). Moreover, the chemical shifts seen in the 1D NMR spectrum as a function of added guest proved difficult to interpret in terms of the limiting binding modes illustrated in Fig. 4 and 5.

### Computational analyses

To gain further insight into the interactions between the ‘two-wall’ C4Ps and **PZDO**, preliminary DFT calculations were performed (see ESI† for more details). Here, CAM-B3LYP was chosen as the density functional since it would allow for more accurate estimations of long-range interactions,^46^ thus allowing account of the putative donor–acceptor π–π interactions between the pyrrole moieties and the bound **PZDO**. Towards this end, ‘two-wall’ C4Ps **2** and **3**, and the control compound **6** were analysed as the case studies. Two plausible 1 : 1 binding modes—a ‘hypothetical mode’ and the ‘experimental mode’—were investigated, wherein the C4P receptor adopts a cone or partial cone conformation, respectively.

DFT-optimised structures of the 1 : 1 host–guest complexes and their relative energies are shown in Fig. S32.† It was found that the 1 : 1 host–guest complexes of **2** and **3** with **PZDO** seen by experiment [*i.e.* (**2·PZDO**)_exp_ and (**3·PZDO**)_exp_] proved less stable than their respective hypothetical counterparts [*i.e.* (**2·PZDO**)_hyp_ and (**3·PZDO**)_hyp_] by *ca.* 4.6 kcal mol^−1^ in the gas phase, whereas the (**6·PZDO**)_exp_ complex proved more stable than its hypothetical counterpart (**6·PZDO**)_hyp_, by 8.2 kcal mol^−1^. These findings provide support for the contention that the C4P subunit *per se* favours the binding of **PZDO** along the lower bowl-like rim, whereas introducing aryl ‘walls’ serves to create cavities into which **PZDO** is bound, with the effect scaling with the number of ‘walls’ (*i.e.*, 4 aryl substituents > 2). However, it is worth noting that contributions from solvation were not considered in these preliminary calculations.^47^ Thus, a clear determination of which limiting binding mode, if any, dominates in solution for the ‘two-wall’ C4Ps **2** and **3** cannot be made at the present time. In contrast, the limits provided by the ‘no-wall’ and ‘four-wall’ receptors (**6** and **5**, respectively) appear much more clearly defined.

DFT analyses were also used to probe the stability of the hypothetical capsule shown in Fig. 1. However, no energy-minimised structures could be found even after extensive computational time. Although not a proof, these findings lead us to infer that the hypothetical capsules formed by the *cis*-‘two-wall’ C4Ps with **PZDO** shown in Fig. 1 might be intrinsically unstable, presumably due to the steric clashes that would be present in such a tightly coupled system.

In contrast, DFT-optimised structures of the experimental 2 : 1 host–guest complexes (**22·PZDO**)_exp_ and (**32·PZDO**)_exp_ converged readily (*cf.* Fig. S33 and S34†). Moreover, as shown in Fig. S35,† these calculated structures are in good agreement with their corresponding single crystal structures. The same proved true for (**62·PZDO**)_exp_ (*cf.* Fig. S36†). Taken in aggregate, these results lead us to suggest that the 2 : 1 host–guest complexes observed in the solid state are energetically reasonable.

## Conclusions

In summary, we report the molecular recognition of **PZDO** using a series of C4P-based receptors (systems **1–6**), both in the solid state and in mixed organic media. The solid-state binding modes between these receptors and **PZDO** were unequivocally determined by means of single-crystal X-ray diffraction analyses. It was found that **PZDO**, unlike **PNO**, was capable of acting as either a pseudo-cation or pseudo-anion in terms of its interactions with the C4Ps receptors. In many cases, a non-axially symmetric binding mode was seen, which has little precedent in the case of C4P·**PNO** complexes. Moreover, in the present study a number of extended structures are seen in the solid state whose specifics depend on the choice of the ‘two-wall’ receptors. ^1^H NMR spectral titrations, NOESY studies and DFT calculations were performed in an effort to gain greater insight into the binding events in solution. It was found that the ‘four-wall’ system bearing four electron-rich ‘walls’, namely receptor **5**, had the highest affinity for **PZDO**, but that the ‘two-wall’ derivatives, **1–3**, were able to stabilise complexes with **PZDO** in CD_3_CN/CD_3_OD (1 : 1 v/v). The present study thus serves to extend our understanding of neutral substrate recognition mediated by C4P-type receptors, while expanding the lexicon of binding modes that this class of well-studied macrocycles is able to support.

## Conflicts of interest

There are no conflicts to declare.

## Supplementary Material

SC-011-D0SC01496F-s001

SC-011-D0SC01496F-s002

SC-011-D0SC01496F-s003
