# Supplementary material for: Molecular recognition of pyrazine N,N′-dioxide using aryl extended calix[4]pyrroles
Source: Chem Sci. 2020 Apr 20;11(22):5650–7. doi: 10.1039/d0sc01496f (PMC8159416; doi:10.1039/d0sc01496f)
Supplement: SC-011-D0SC01496F-s001 [file SC-011-D0SC01496F-s001.pdf]

Electronic Supplementary Material (ESI) for Chemical Science.
This journal is © The Royal Society of Chemistry 2020

Supporting Information for

Molecular recognition of pyrazine *N,N'*-dioxide using aryl extended calix[4]pyrroles†

Chenxing Guo,[a,‡] Hu Wang,[a,‡] Vincent M. Lynch,[a] Xiaofan Ji,*[b] Zachariah A. Page*[a] and Jonathan L. Sessler*[a]

[a] Department of Chemistry, The University of Texas at Austin, 105 East 24th Street, Stop A5300, Austin, Texas 78712, United States.

[b] School of Chemistry and Chemical Engineering, Huazhong University of Science and Technology (HUST), Wuhan 430074, China.

‡ These two authors contributed equally.

*Corresponding Authors: Xiaofan Ji (xiaofanji@hust.edu.cn), Zachariah A. Page (zpage@cm.utexas.edu) and Jonathan L. Sessler (sessler@cm.utexas.edu)

Table of Contents

1. General information S2
2. Synthesis and characterisation S3
3. Binding studies in solution S4
4. Computational analyses S23
5. Structural information derived from single-crystal diffraction analyses S36
6. X-ray experimental S39
7. ^{1}H NMR, ^{13}C NMR and HRMS spectra S45
8. References S48

1. General information

All reagents and solvents were purchased from commercial suppliers (Fisher, Sigma Aldrich, Acros Organics, *etc.*) and used without further purification. Analytical thin-layer chromatography (TLC) was performed on commercially available precoated silica gel plates containing a fluorescent indicator. Flash column chromatography was carried out using silica gel (230–400 mesh). High-resolution mass spectra (HRMS) were measured by an Agilent 6530 Q-TOF. ^{1}H and ^{13}C NMR spectra were recorded on a Varian 400 spectrometer at room temperature. Chemical shifts are reported in ppm using residual solvent signals as the internal reference. All NMR spectroscopic solvents were purchased from Cambridge Isotope Laboratories. Isothermal titration calorimetry (ITC) was performed using a MicroCal™ VP-ITC microcalorimeter.

Binding Studies. ^{1}H NMR spectral titrations were carried out in a 1:1 mixture of CD_3CN/CD_3OD. The concentration of the host in question was kept constant over the course of experiment while increasing quantities of **PZDO** were added to the solution. The binding stoichiometry was determined by means of the associated mole ratio plot and by assessing the goodness of fit to 1:1 and 2:1 binding models.

In the case of a presumed 1:1 binding event, the following equation was used to determine the association constant corresponding to the interaction between the C4P host and the **PZDO** guest:

$$\Delta\delta = (\Delta\delta_\infty/[G])\,(0.5[H]_0 + 0.5([G] + 1/K_a) - (0.5([H]_0^2 + (2[H]_0(1/K_a - [G])) + (1/K_a + [G])^2)^{0.5})) \qquad \textbf{(Eq. S1)}$$

where $\Delta\delta$ is the change in chemical shift associated with any chosen peak, $\Delta\delta_\infty$ is the saturated change in chemical shift associated with the chosen peak at the end of the titration, $[H]_0$ is the initial concentration of host (5.00 mM), and [G] is the variable corresponding to the concentration of **PZDO**.

OriginPro was used to fit data in a non-linear fashion in accord with the above equation. Iterations (choosing a fixed *a* value and changing $\Delta\varepsilon$ and K) were performed until an acceptable level of convergence was reached (chi-square tolerance value $\leq 1 \times 10^{-9}$).

In the case of a presumed 2:1 binding event, the stepwise binding constants (K_{11} and K_{21}) were determined using the BindFit web applet at www.supramolecular.org.[S3] For consistency, this same applet was used to double-check the 1:1 fits.

Computational Studies. The DFT calculations for generating the ELPOT maps of **PNO** and **PZDO** were carried out with the Spartan Student V7 software[S1] using the B3LYP density functional. The DFT computational analyses for optimizing the geometries of host–guest complexes were carried out with the Gaussian 16 suite of programs[S2] using the CAM-B3LYP density functional. All computational studies were carried out in the gas phase. A 6-31G* basis set was used for all atoms. The obtained crystal structures served as the starting coordinates for the geometry optimization of the observed host–guest complexes.

2. Synthesis and characterisation

Compounds **1**–**3**, **5**, **6** and **PZDO** were all prepared according to reported procedures.[S4–S9] The two-step synthesis of compound **4** is detailed below:

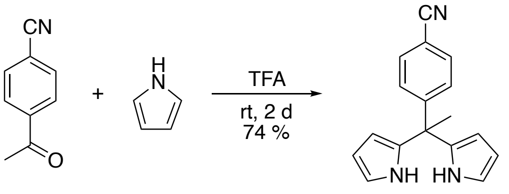

7

4-(1,1-Di(1H-pyrrol-2-yl)ethyl)benzonitrile (*7*). 4-Acetylbenzonitrile (1.48 g, 10.0 mmol) was dissolved in freshly distilled pyrrole (13.42 g, 200 mmol). Trifluoroacetic acid (TFA) (500 μL) was added to the mixture, and the resulting reaction mixture was stirred at room temperature for 2 days. Excess triethylamine (*ca.* 3 mL) was added to quench the reaction. After the removal of the unreacted pyrrole *in vacuo* (the temperature of the water bath and the pressure were set to 80 ºC and 80 bar, respectively), the dark brown residue was subjected directly to column chromatography over silica gel (eluent: dichloromethane). This yielded the desired product **7** as a white powder in 74% yield (1.92 g). ^{1}H NMR (400 MHz, $CDCl_3$): δ 7.82 (s, 4H, N*H* (pyrrole)) 7.56 (d, 4H, J = 8.4 Hz Ar*H*), 7.21 (d, 4H, J = 8.4 Hz Ar*H*), 6.72–6.71 (m, 2H, Ar*H* (pyrrole)), 6.20–6.17 (m, 2H, Ar*H* (pyrrole)), 5.94–5.92 (m, 2H Ar*H* (pyrrole)), 2.05 (s, 3H, C*H*$_3$). ^{13}C NMR (100 MHz, $CDCl_3$): δ 153.1, 136.0, 132.0, 128.4, 119.0, 117.8, 110.6, 108.6, 107.0, 45.2, 28.6. HRMS (ESI+) *m*/*z*: [M + H]$^+$ calcd for $C_{17}H_{16}N_3$, 262.1339; found, 362.1341.

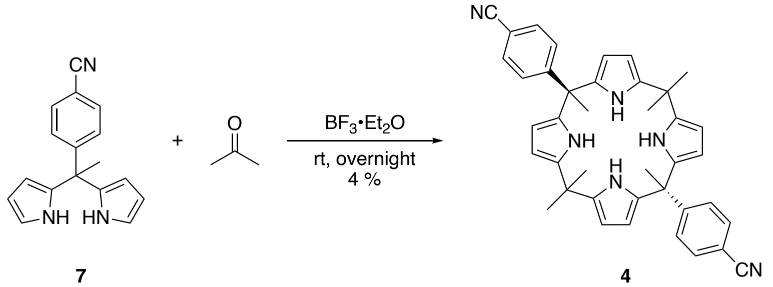

7 **4**

2,2'-(1-(Pyren-1-yl)ethane-1,1-diyl)bis(1H-pyrrole) (*4*). Dipyrromethane **7** (1.42 g, 5.43 mmol) was dissolved in 150 mL of acetone. Boron trifluoride diethyl etherate ($BF_3{\cdot}OEt_2$) (46.5%, 0.5 mL) was added to the mixture dropwise, and the resulting reaction mixture was stirred overnight at room temperature (*ca.* 16 h). Triethylamine (3 mL) was added to quench the reaction. After removing the volatiles *in vacuo*, the residue was dissolved in 100 mL of dichloromethane and washed with brine (2 × 100 mL). The organic phase was separated off and dried over anhydrous Na_2SO_4. The solvent was removed under reduced pressure, and the resulting residue was purified by column chromatography over silica gel (eluent: dichloromethane) to afford a pale yellow solid (116 mg, 4% yield). ^{1}H NMR (400 MHz, CD_2Cl_2): 7.58 (d, 4H, J = 8.4 Hz Ar*H*), δ 7.29 (s, 4H, N*H* (pyrrole)), 7.23 (d, 4H, J = 8.4 Hz Ar*H*), 5.96–5.94 (t, 4H, Ar*H* (pyrrole)), 5.77–5.75 (t, 4H Ar*H* (pyrrole)), 1.90 (s, 6H, C*H*$_3$), 1.54 (s, 12H, C*H*$_3$). ^{13}C NMR (100 MHz, CD_2Cl_2): δ 152.7, 139.4, 135.5, 132.1, 128.6, 119.2, 110.8, 106.5, 103.9, 45.4, 35.6, 29.3, 29.2. HRMS (ESI+) *m*/*z*: [M + H]$^+$ calcd for $C_{40}H_{39}N_6$, 603.3231; found, 603.3235.

3. Binding studies in solution

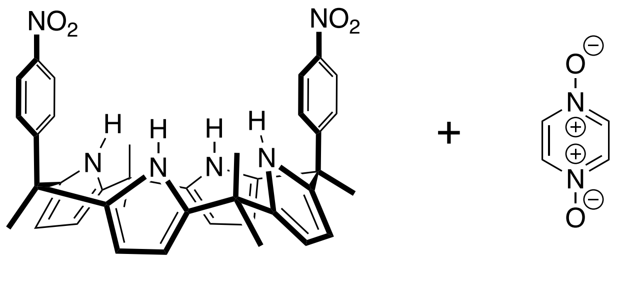

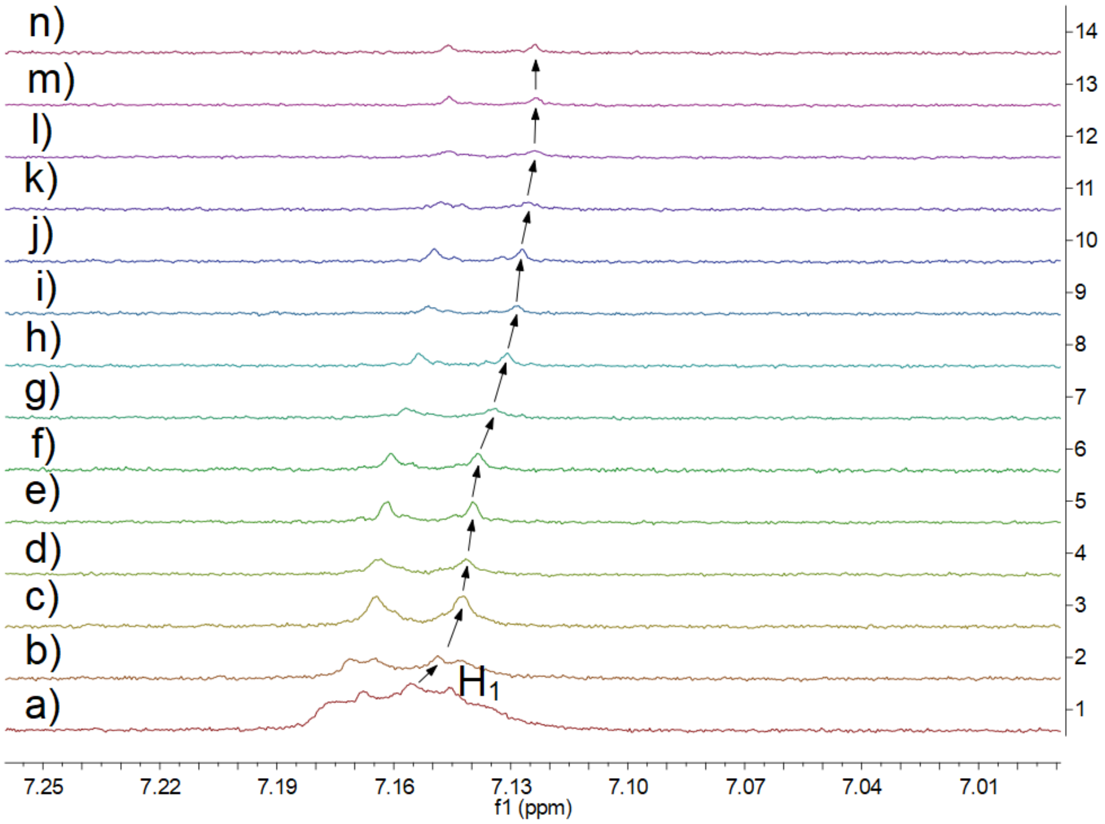

Fig. S1 ^{1}H NMR spectra of **1** in a 1:1 mixture of CD_3CN/CD_3OD recorded at a concentration of 5.00 mM in the presence of increasing concentrations of **PZDO**: (a) 0.00 mM; (b) 1.18 mM; (c) 2.23 mM; (d) 3.18 mM; (e) 4.03 mM; (f) 4.8 mM; (g) 6.94 mM; (h) 8.58 mM; (i) 9.87 mM; (j) 10.91 mM; (k) 11.77 mM; (l) 12.5 mM; (m) 13.65 mM; (n) 14.52 mM.

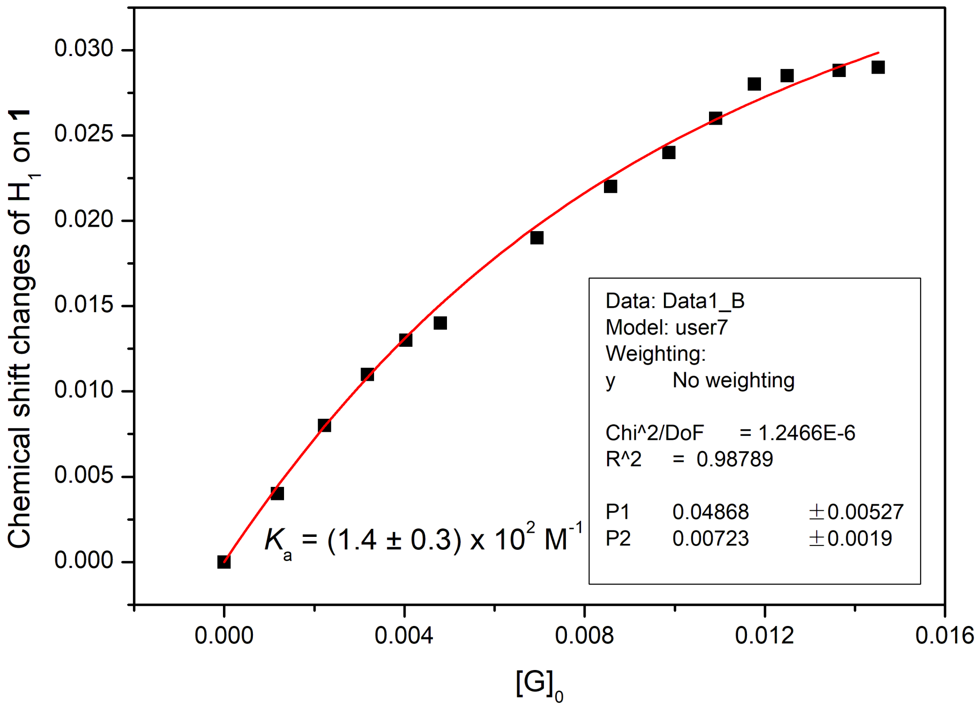

Fig. S2 Changes in the chemical shift corresponding to the H1 peak of **1** as a function of added **PZDO**. The red solid line was obtained from a non-linear curve-fitting using **Eq. S1**.

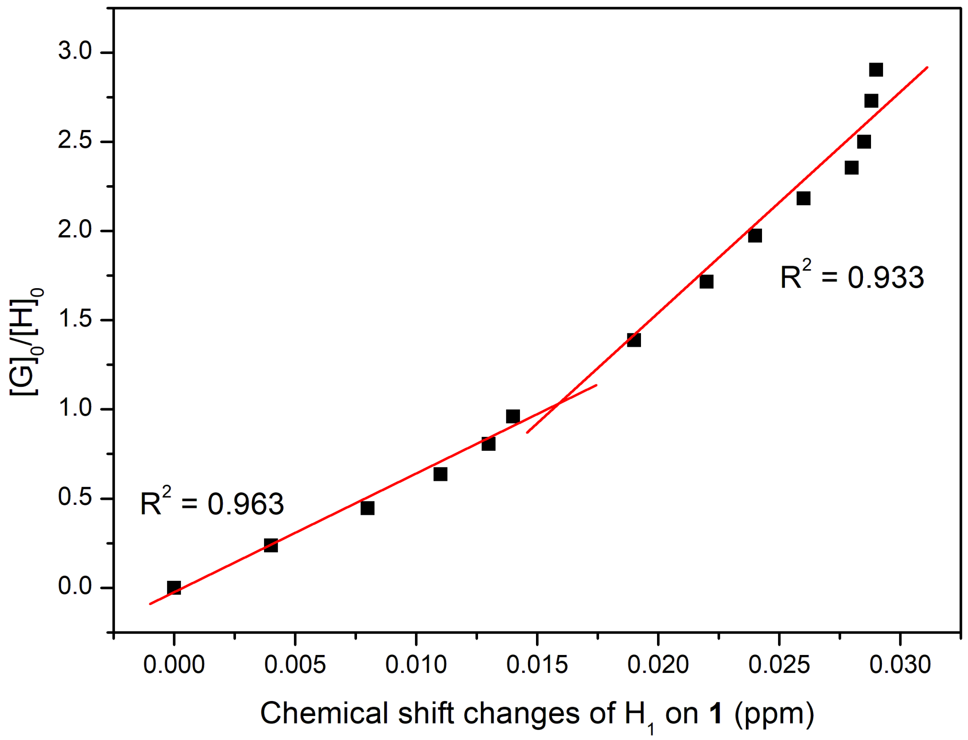

Fig. S3 Mole ratio plot for the interaction between **1** and **PZDO**. The result is consistent with a 1:1 binding stoichiometry.

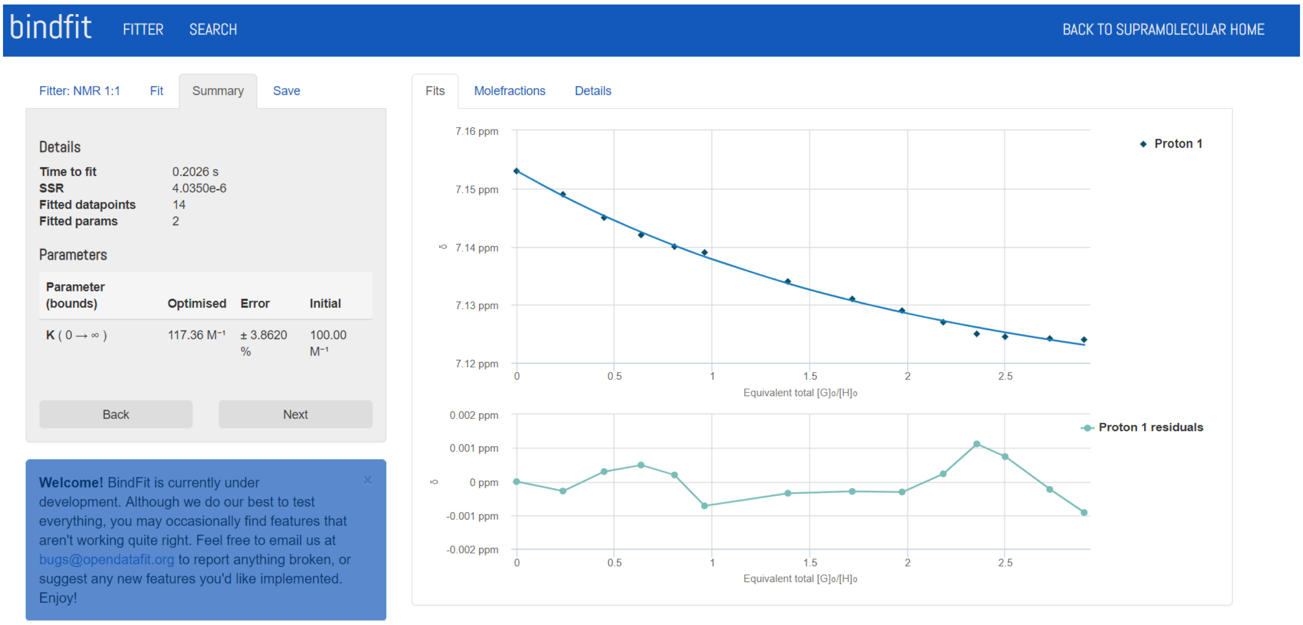

Fig. S4 Least-squares non-linear fitting of the changes in the chemical shift corresponding to the H_1 peak of **1** as a function of added **PZDO**. The solid lines were obtained from non-linear curve-fitting to a 1:1 binding model using the www.supramolecular.org web applet.

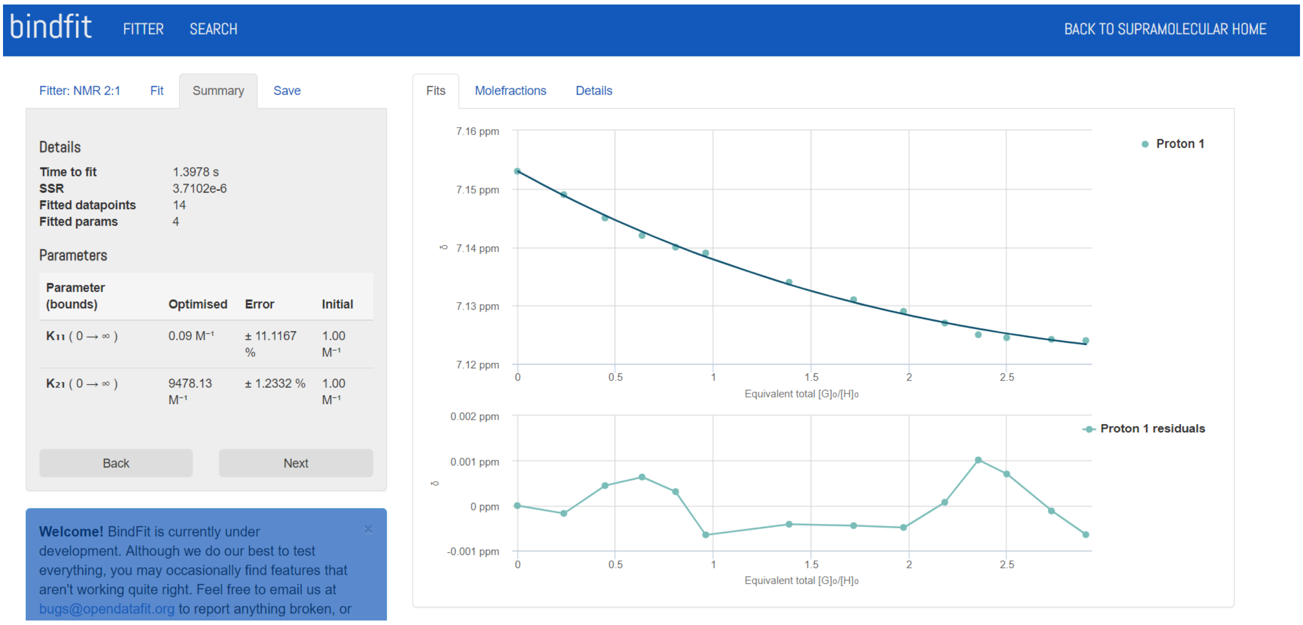

Fig. S5 Least-squares non-linear fitting of the changes in the chemical shift corresponding to the H_1 peak of **1** as a function of added **PZDO**. The solid lines were obtained from non-linear curve-fitting to a 2:1 binding model using the www.supramolecular.org web applet.

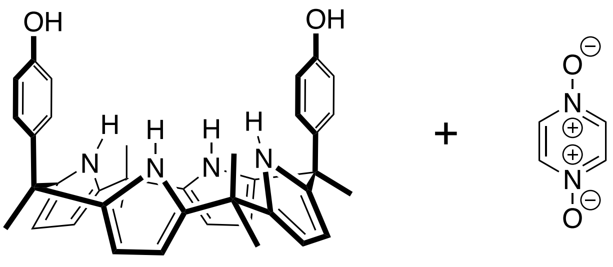

2 **PZDO**

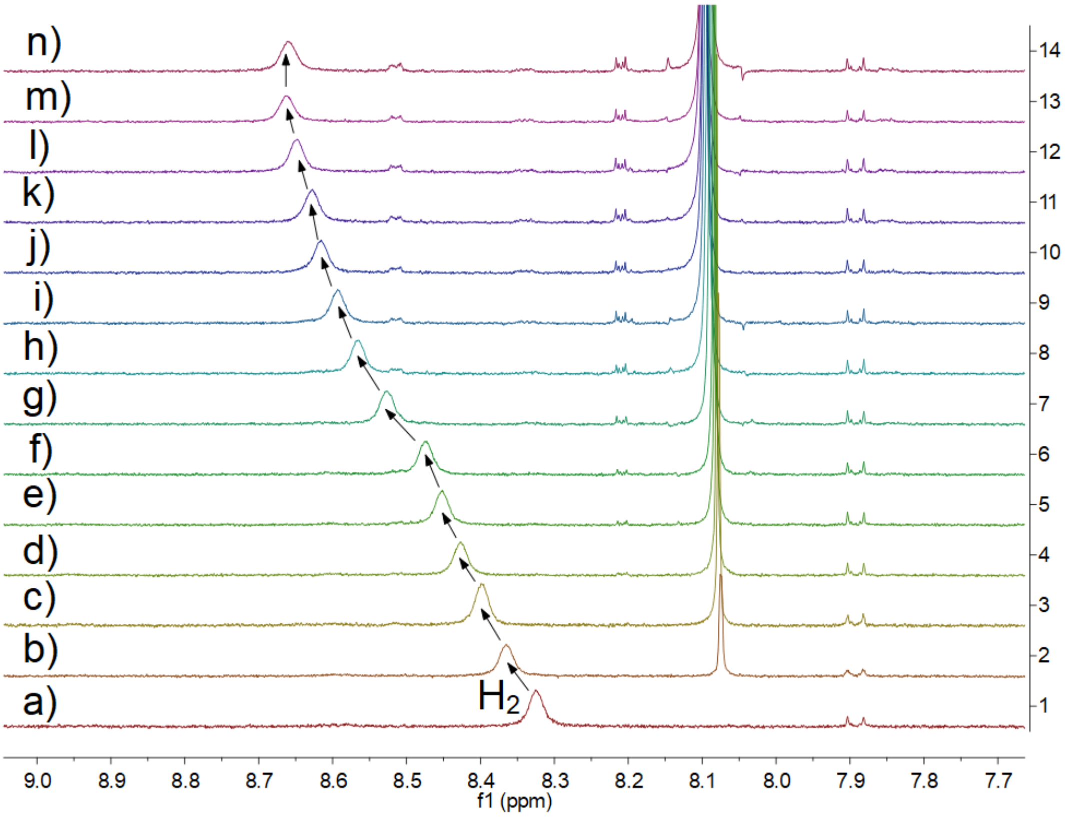

Fig. S6 ^{1}H NMR spectra of **2** in a 1:1 mixture of CD_3CN/CD_3OD recorded at a concentration of 5.00 mM in the presence of different concentrations of **PZDO**: (a) 0.00 mM; (b) 1.18 mM; (c) 2.23 mM; (d) 3.18 mM; (e) 4.03 mM; (f) 4.8 mM; (g) 6.94 mM; (h) 8.58 mM; (i) 9.87 mM; (j) 10.91 mM; (k) 11.77 mM; (l) 12.5 mM; (m) 13.65 mM; (n) 14.52 mM.

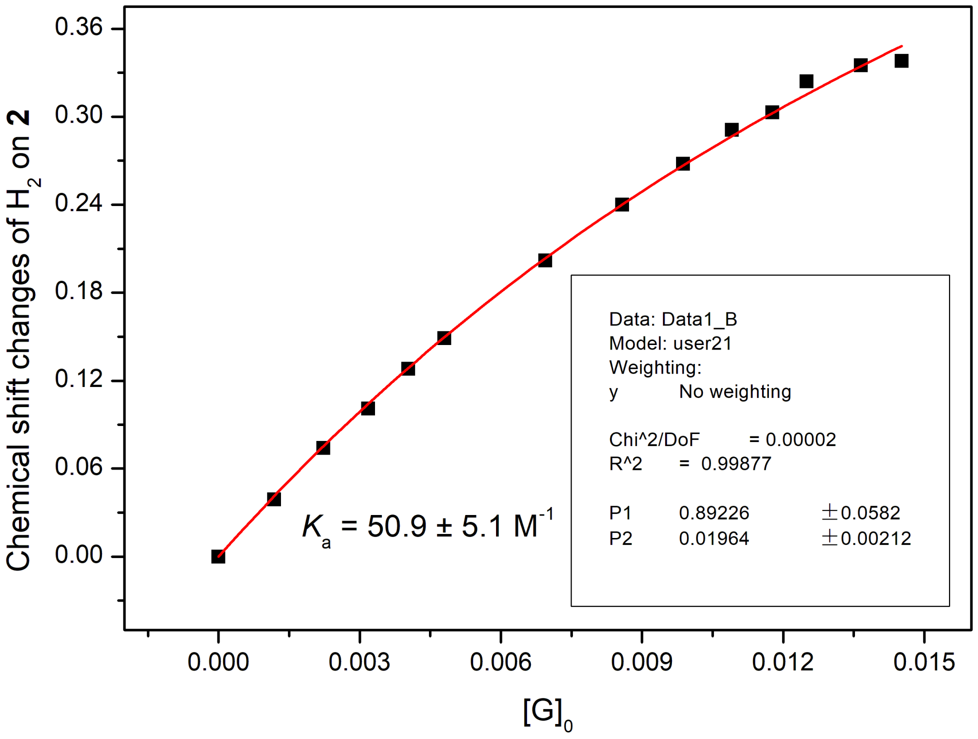

Fig. S7 Changes in the chemical shift corresponding to H_2 peak of **2** as a function of added **PZDO**. The red solid line was obtained from a non-linear curve-fitting using **Eq. S1**.

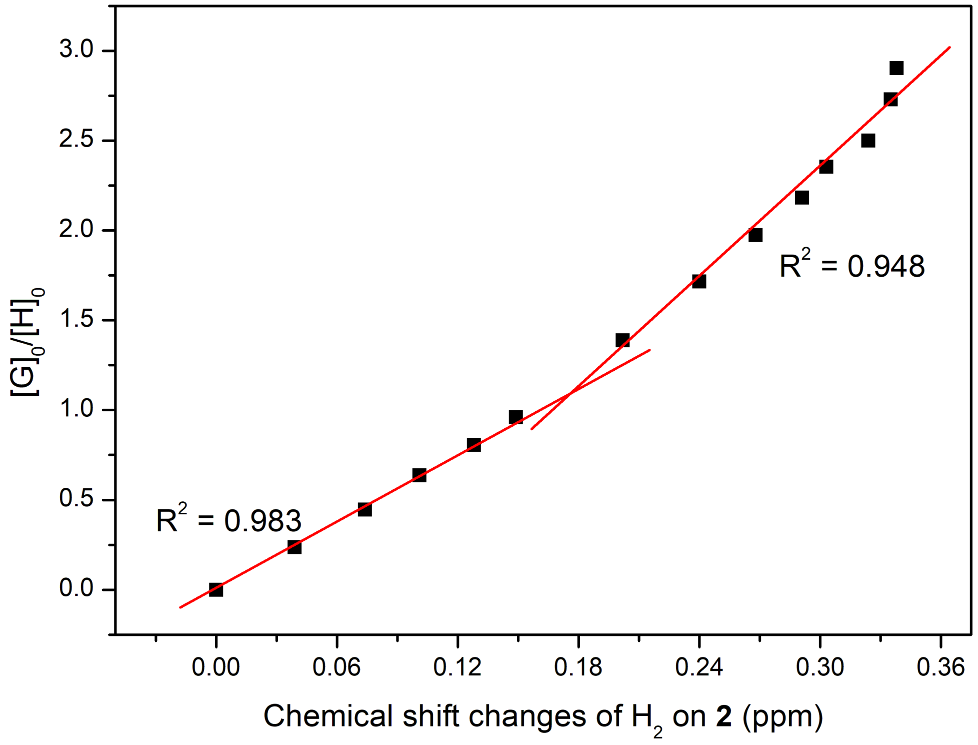

Fig. S8 Mole ratio plot for the interaction between **2** and **PZDO**. The result is consistent with a 1:1 binding stoichiometry.

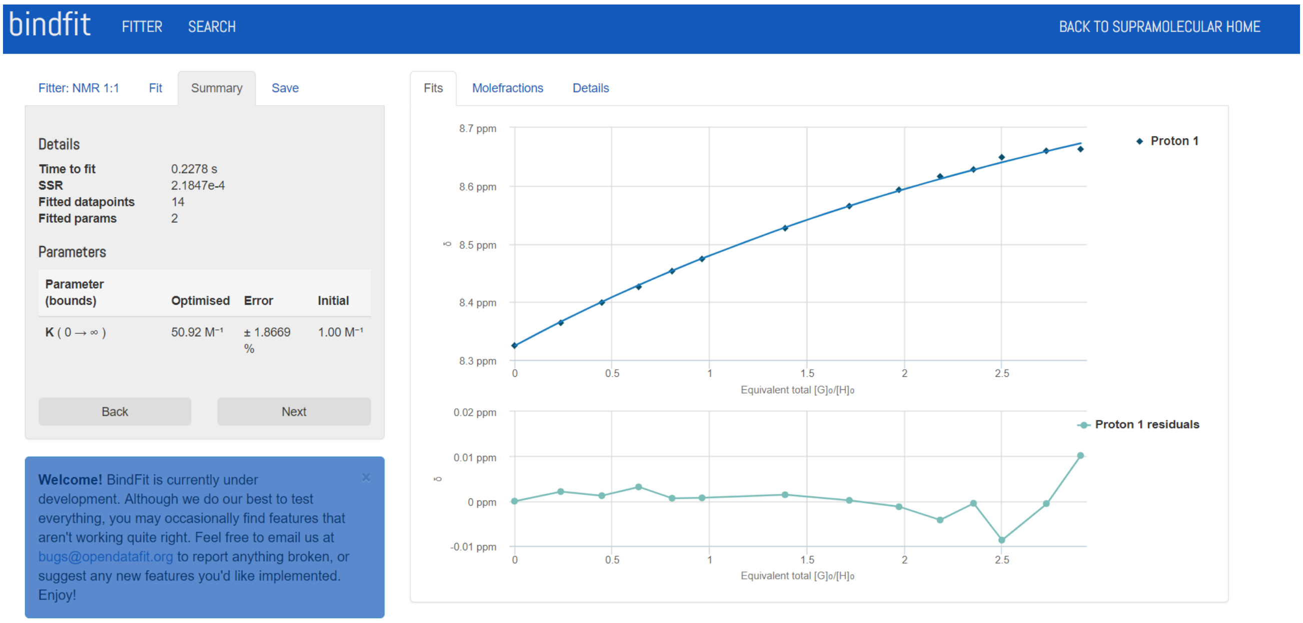

Fig. S9 Least-squares non-linear fitting of the changes in the chemical shift corresponding to the H_2 peak of **2** as a function of added **PZDO**. The solid lines were obtained from non-linear curve-fitting to a 1:1 binding model using the www.supramolecular.org web applet.

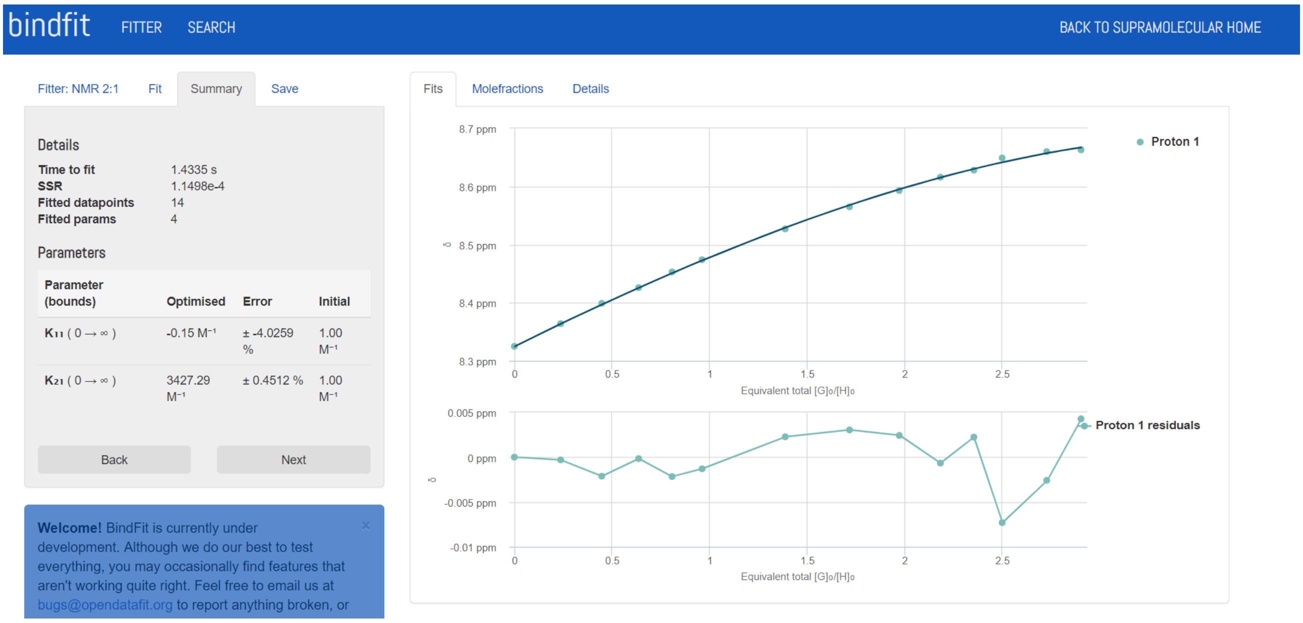

Fig. S10 Least-squares non-linear fitting of the changes in the chemical shift corresponding to the H_2 peak of **2** as a function of added **PZDO**. The solid lines were obtained from non-linear curve-fitting to a 2:1 binding model using the www.supramolecular.org web applet.

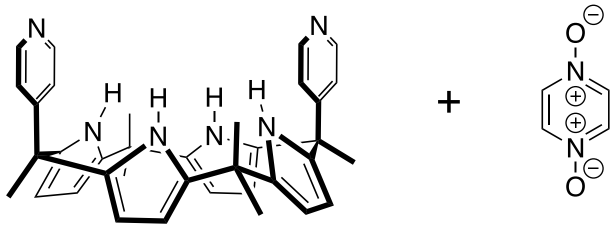

3 PZDO

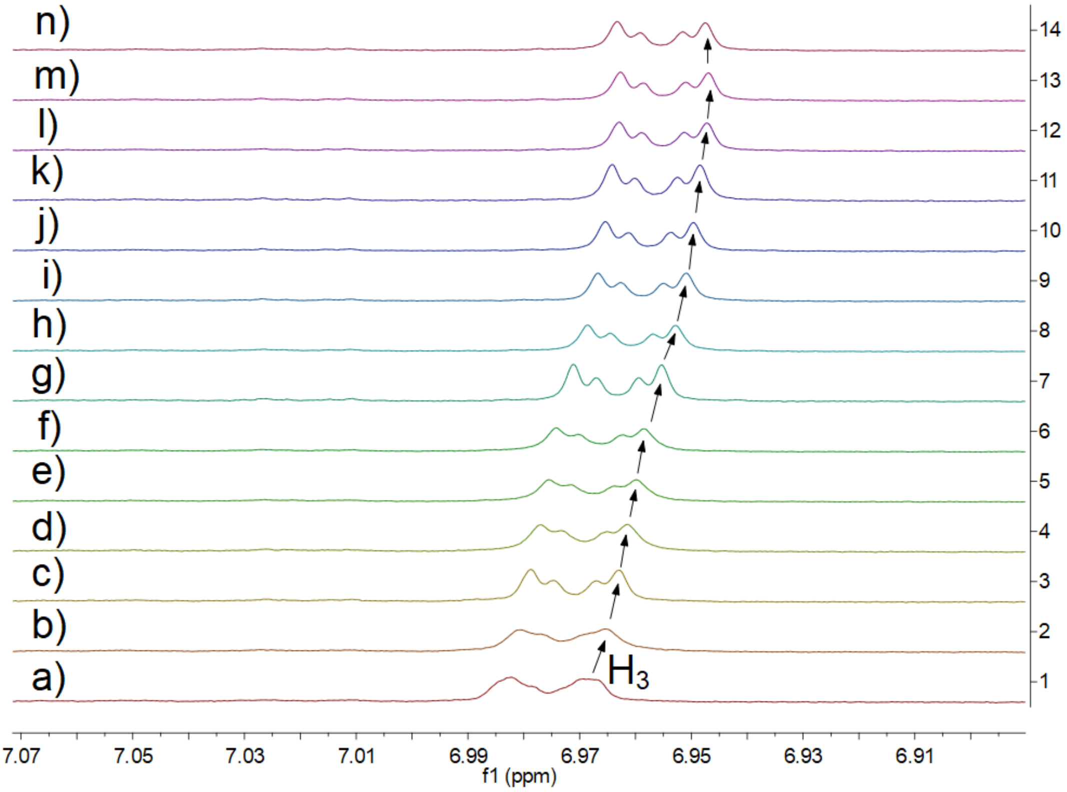

Fig. S11 ^{1}H NMR spectra of **3** in a 1:1 mixture of CD_3CN/CD_3OD recorded at a concentration of 5.00 mM in the presence of increasing concentrations of **PZDO**: (a) 0.00 mM; (b) 1.18 mM; (c) 2.23 mM; (d) 3.18 mM; (e) 4.03 mM; (f) 4.8 mM; (g) 6.94 mM; (h) 8.58 mM; (i) 9.87 mM; (j) 10.91 mM; (k) 11.77 mM; (l) 12.5 mM; (m) 13.65 mM; (n) 14.52 mM.

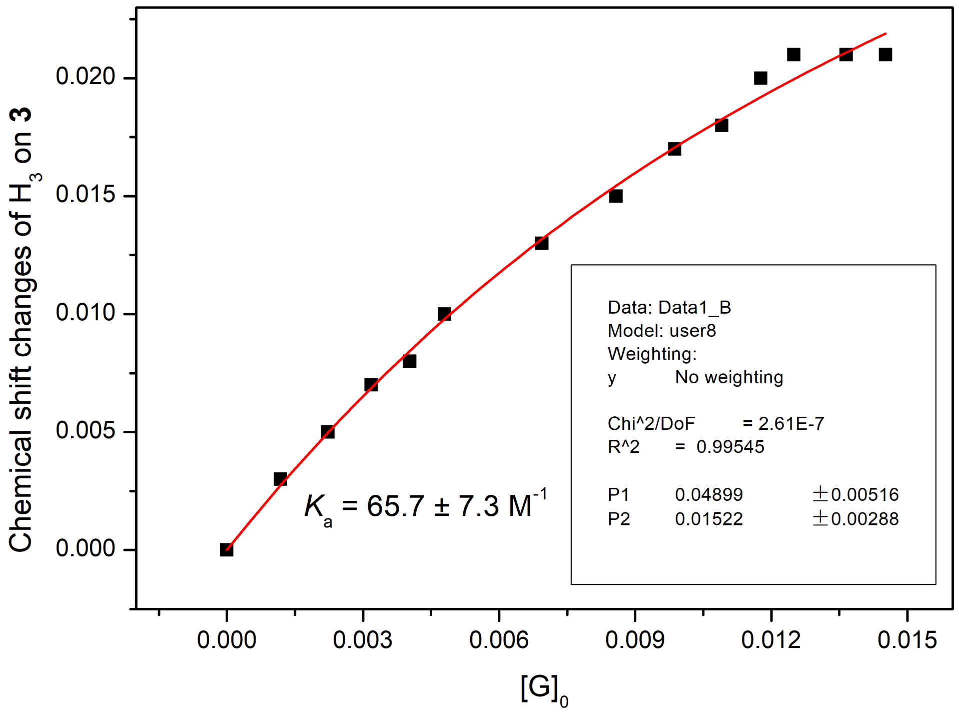

Fig. S12 Changes in the chemical shift corresponding to H3 peak of **3** as a function of the added **PZDO**. The red solid line was obtained from a non-linear curve-fitting using **Eq. S1**.

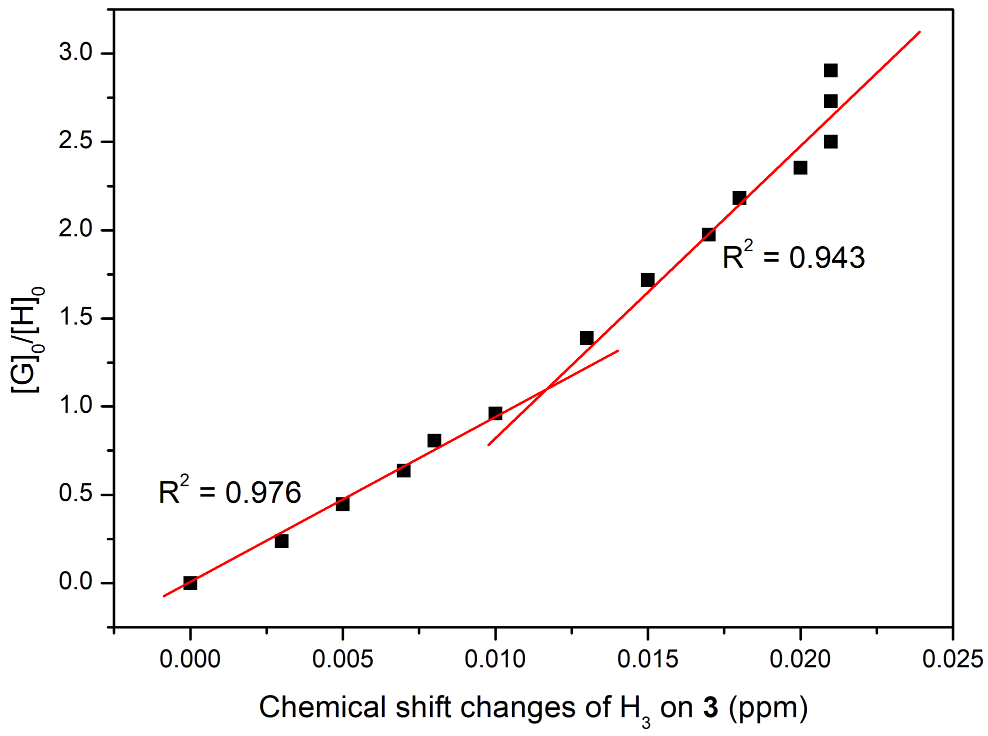

Fig. S13 Mole ratio plot for the complexation between **3** and **PZDO**. The result is consistent with a 1:1 binding stoichiometry.

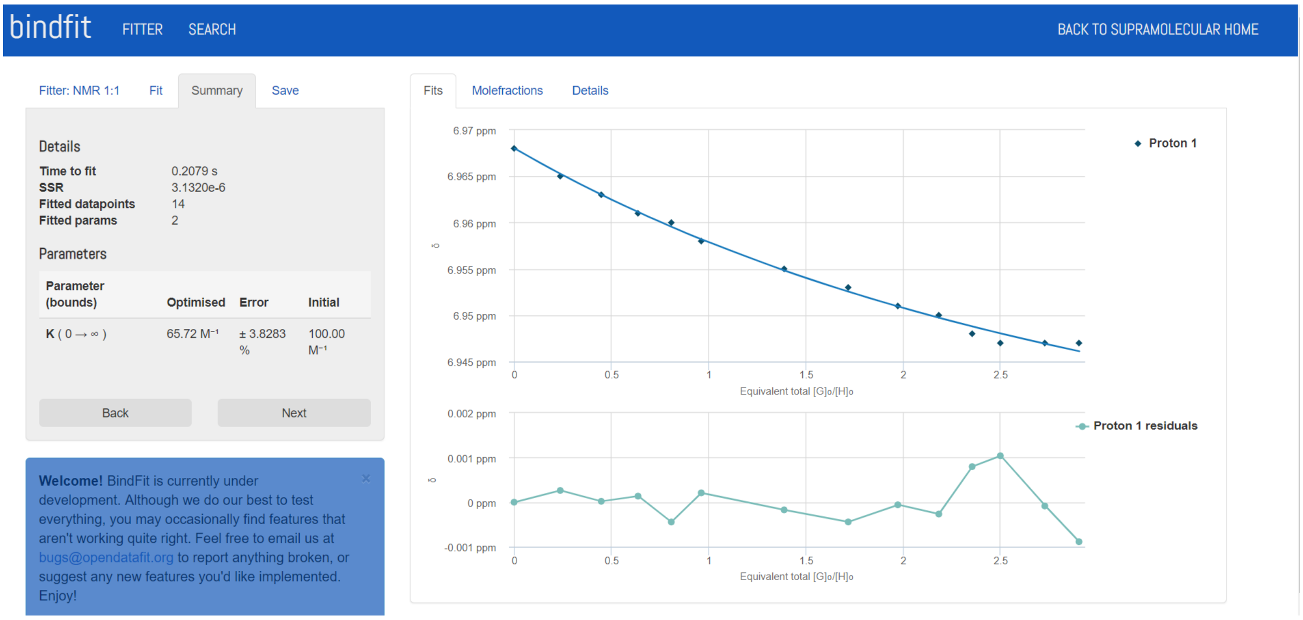

Fig. S14 Least-squares non-linear fitting of the changes in the chemical shift corresponding to the H_3 peak of **3** as a function of added **PZDO**. The solid lines were obtained from non-linear curve-fitting to a 1:1 binding model using the www.supramolecular.org web applet.

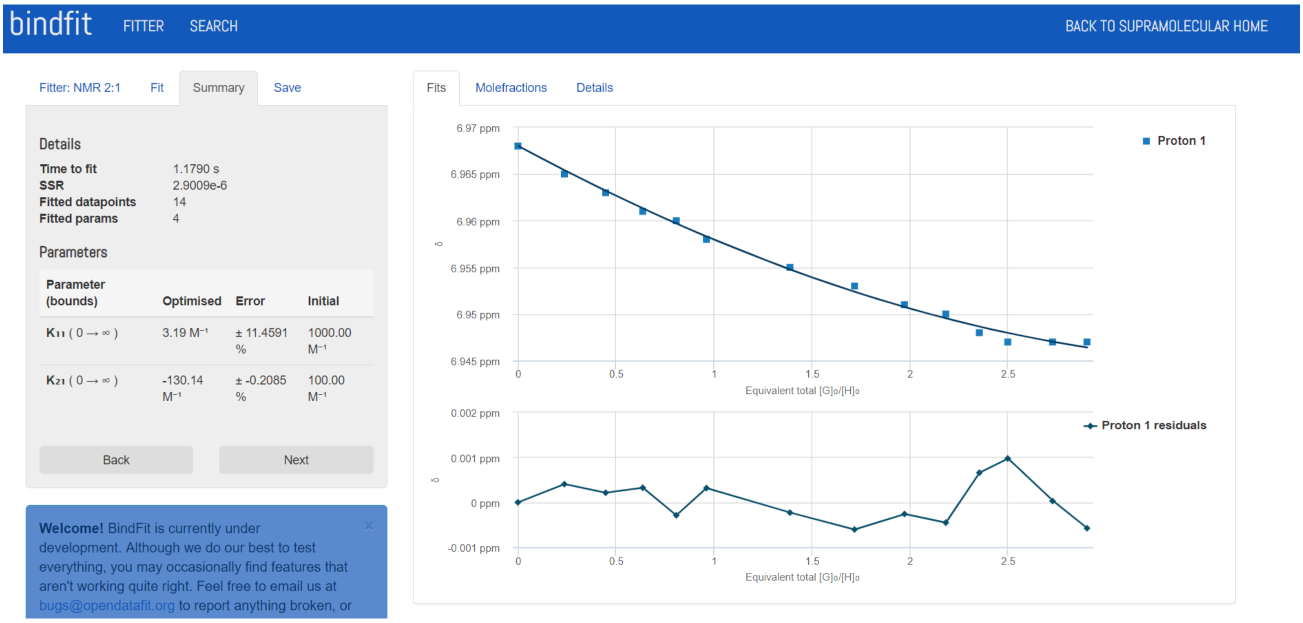

Fig. S15 Least-squares non-linear fitting of the changes in the chemical shift corresponding to the H_3 peak of **3** as a function of added **PZDO**. The solid lines were obtained from non-linear curve-fitting to a 2:1 binding model using the www.supramolecular.org web applet.

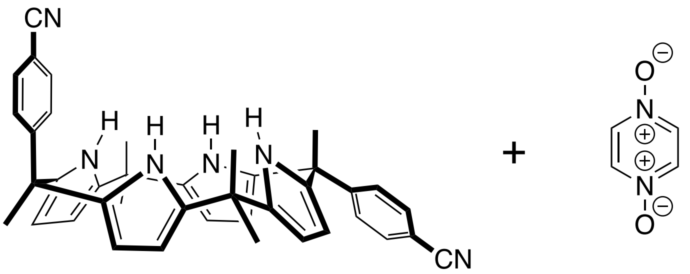

4 **PZDO**

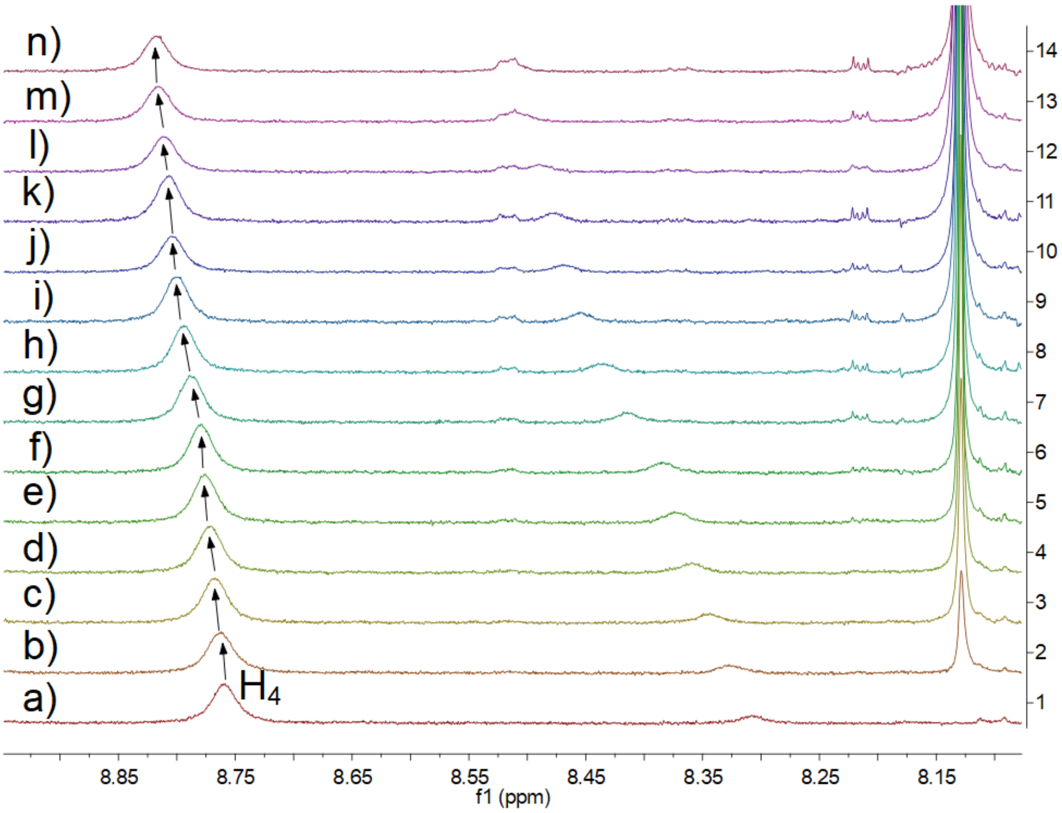

Fig. S16 ^{1}H NMR spectra of **4** in a 1:1 mixture of CD_3CN/CD_3OD recorded at a concentration of 5.00 mM in the presence of increasing concentrations of **PZDO**: (a) 0.00 mM; (b) 1.18 mM; (c) 2.23 mM; (d) 3.18 mM; (e) 4.03 mM; (f) 4.8 mM; (g) 6.94 mM; (h) 8.58 mM; (i) 9.87 mM; (j) 10.91 mM; (k) 11.77 mM; (l) 12.5 mM; (m) 13.65 mM; (n) 14.52 mM.

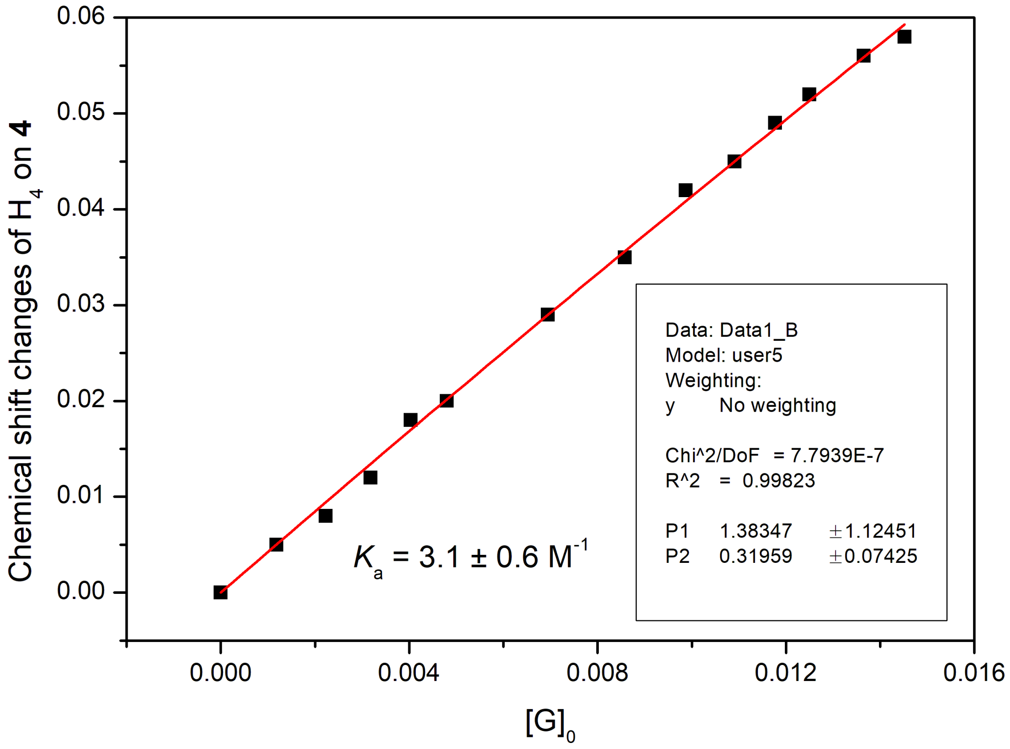

Fig. S17 Changes in the chemical shift corresponding to H_4 peak of **4** as a function of the added **PZDO**. The red solid line was obtained from a non-linear curve-fitting using **Eq. S1**.

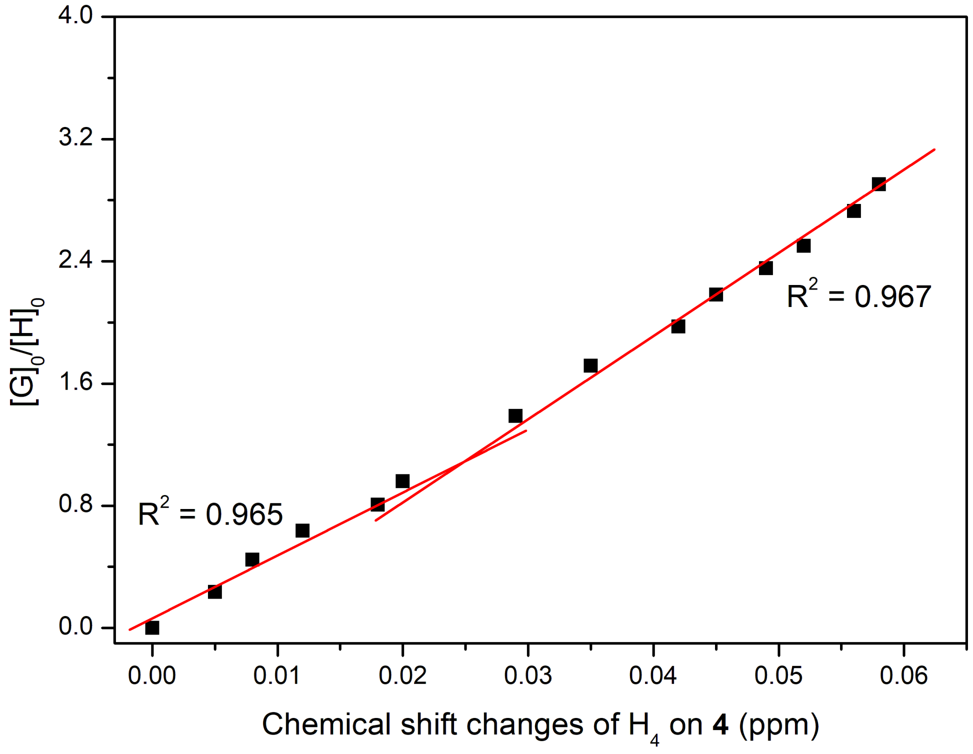

Fig. S18 Mole ratio plot for the complexation between **4** and **PZDO**. The result is consistent with a 1:1 binding stoichiometry.

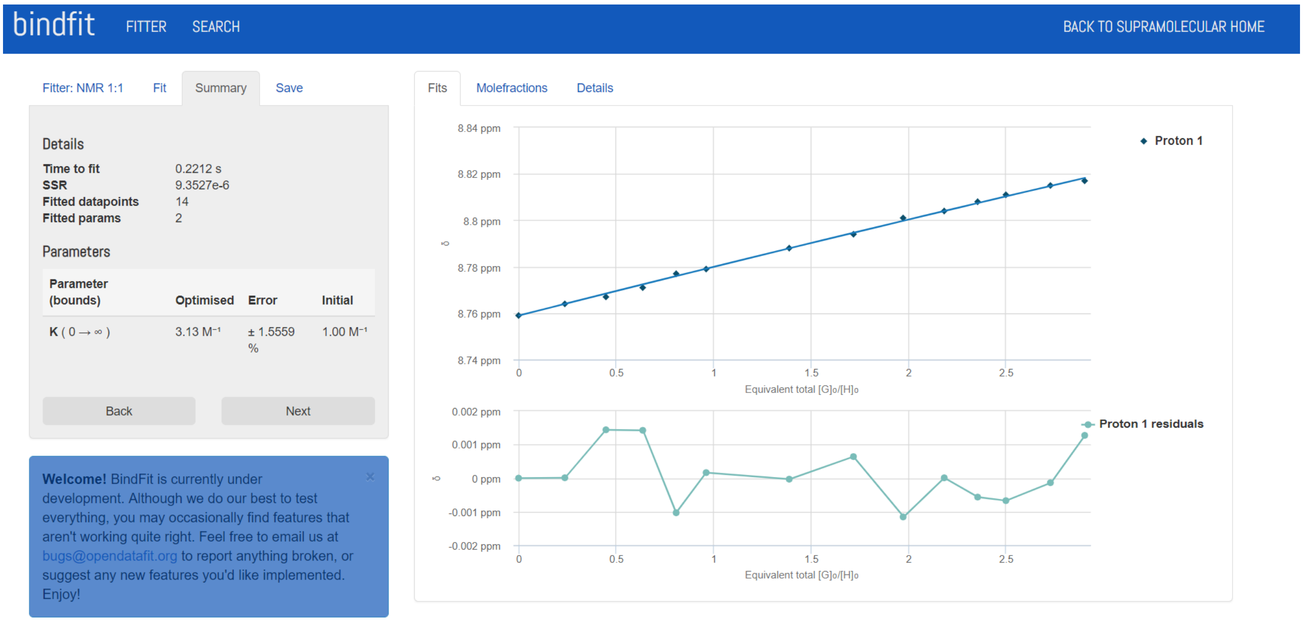

Fig. S19 Least-squares non-linear fitting of the changes in the chemical shift corresponding to the H_4 peak of **4** as a function of added **PZDO**. The solid lines were obtained from non-linear curve-fitting to a 1:1 binding model using the www.supramolecular.org web applet.

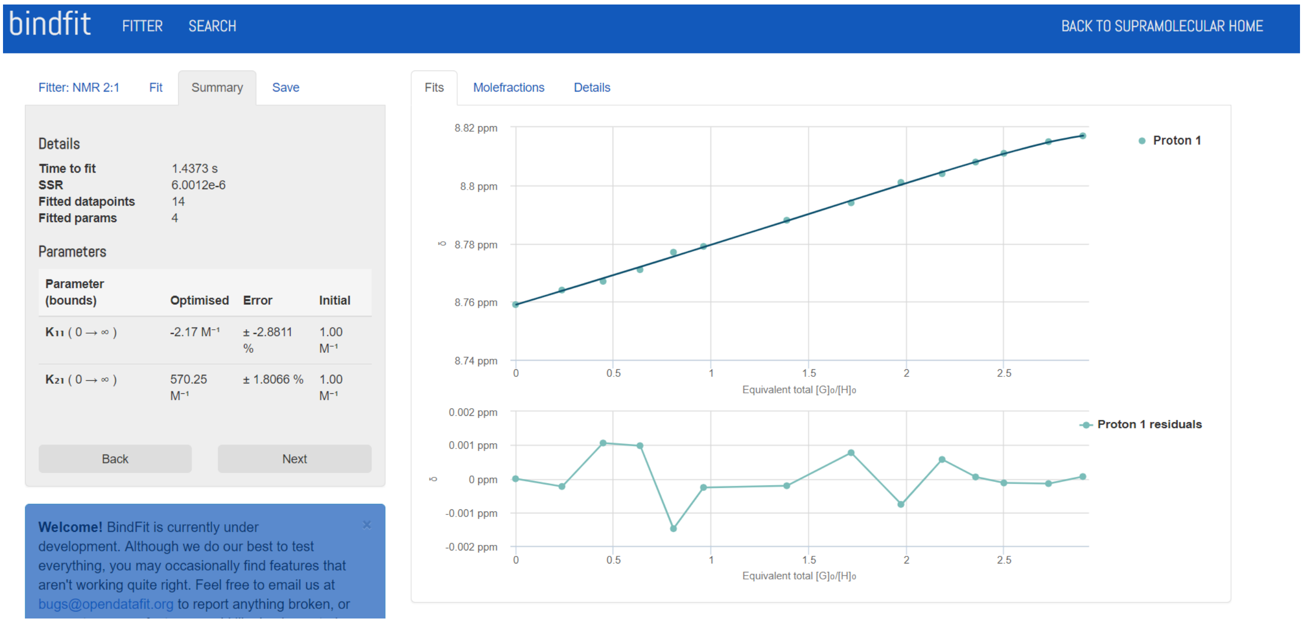

Fig. S20 Least-squares non-linear fitting of the changes in the chemical shift corresponding to the H_4 peak of **4** as a function of added **PZDO**. The solid lines were obtained from non-linear curve-fitting to a 2:1 binding model using the www.supramolecular.org web applet.

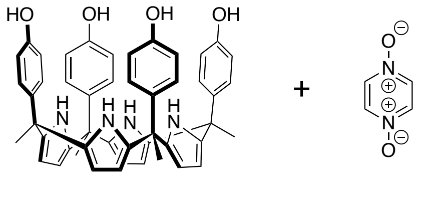

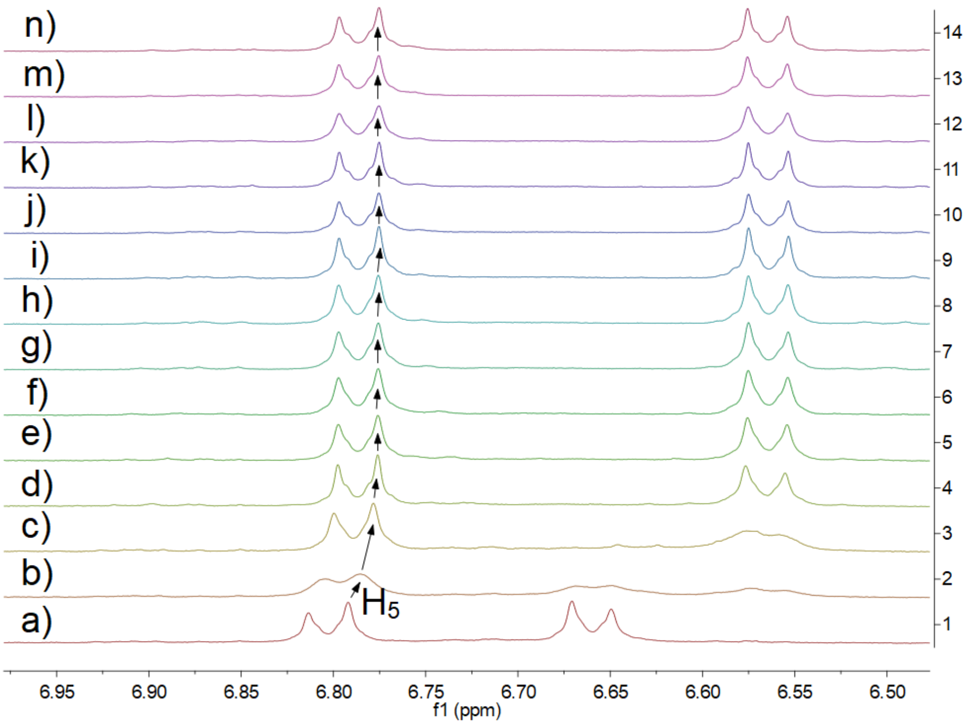

Fig. S21 ^{1}H NMR spectra of **5** in a 1:1 mixture of CD3CN/CD3OD recorded at a concentration of 5.00 mM in the presence of increasing concentrations of **PZDO**: (a) 0.00 mM; (b) 1.18 mM; (c) 2.23 mM; (d) 3.18 mM; (e) 4.03 mM; (f) 4.8 mM; (g) 6.94 mM; (h) 8.58 mM; (i) 9.87 mM; (j) 10.91 mM; (k) 11.77 mM; (l) 12.5 mM; (m) 13.65 mM; (n) 14.52 mM.

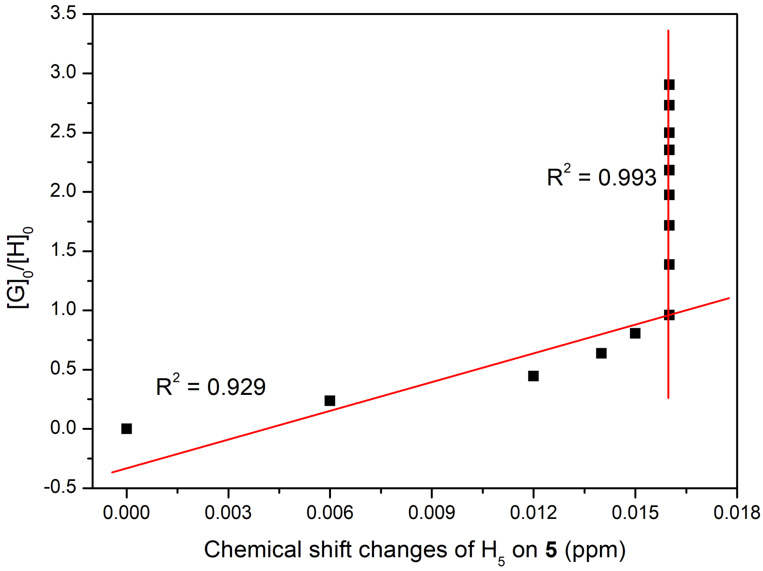

Fig. S22 Mole ratio plot for the complexation between **5** and **PZDO**. The result is consistent with a 1:1 binding stoichiometry.

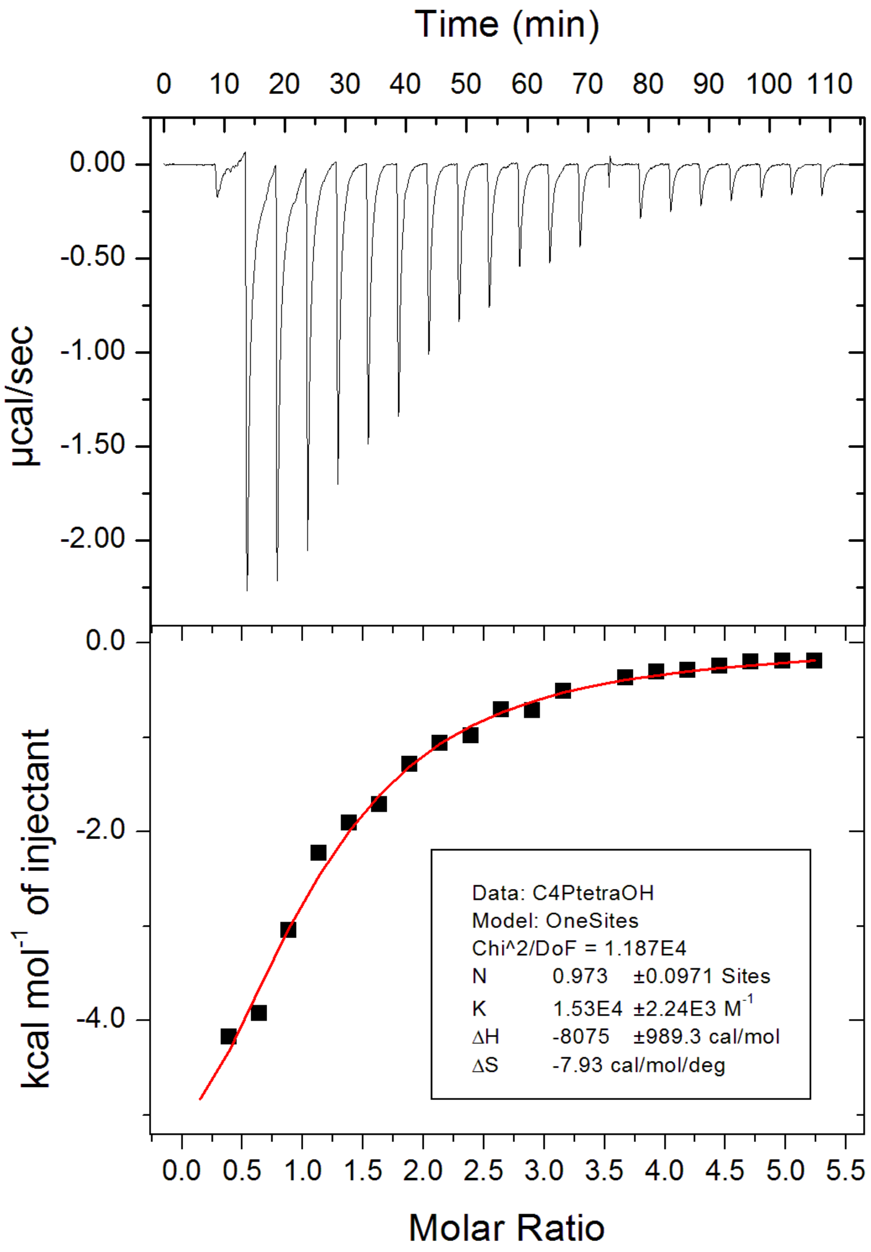

Fig. S23 Raw ITC curve (top panel) and the corresponding binding isotherm with the fitting curve (bottom panel) for the titration of **5** (0.10 mM) with **PZDO** in CH_3CN/CH_3OH (1:1 v/v) at 298 K.

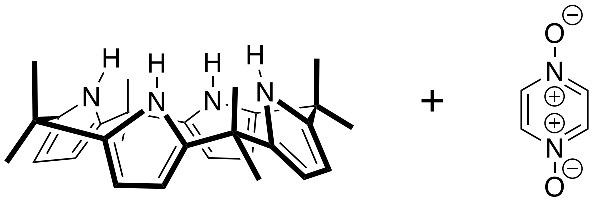

6 **PZDO**

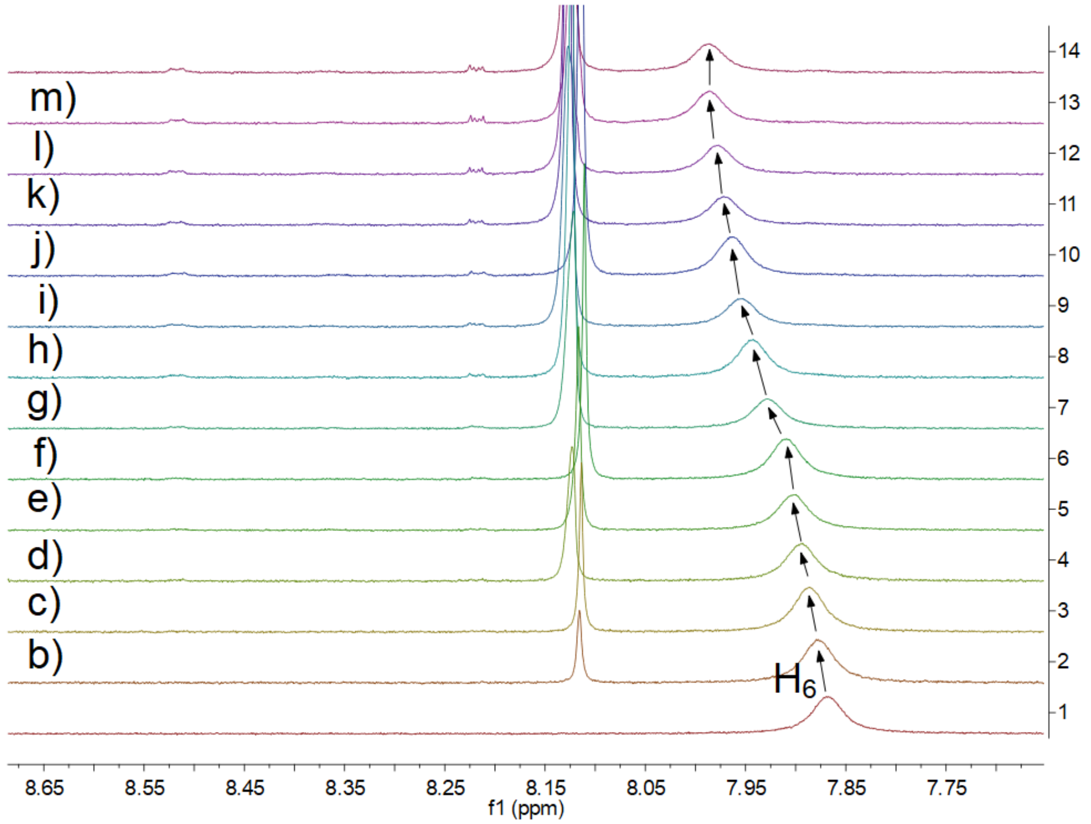

Fig. S24 ^{1}H NMR spectra of **6** in a 1:1 mixture of CD_3CN/CD_3OD recorded at a concentration of 5.00 mM in the presence of increasing concentrations of **PZDO**: (a) 0.00 mM; (b) 1.18 mM; (c) 2.23 mM; (d) 3.18 mM; (e) 4.03 mM; (f) 4.8 mM; (g) 6.94 mM; (h) 8.58 mM; (i) 9.87 mM; (j) 10.91 mM; (k) 11.77 mM; (l) 12.5 mM; (m) 13.65 mM; (n) 14.52 mM.

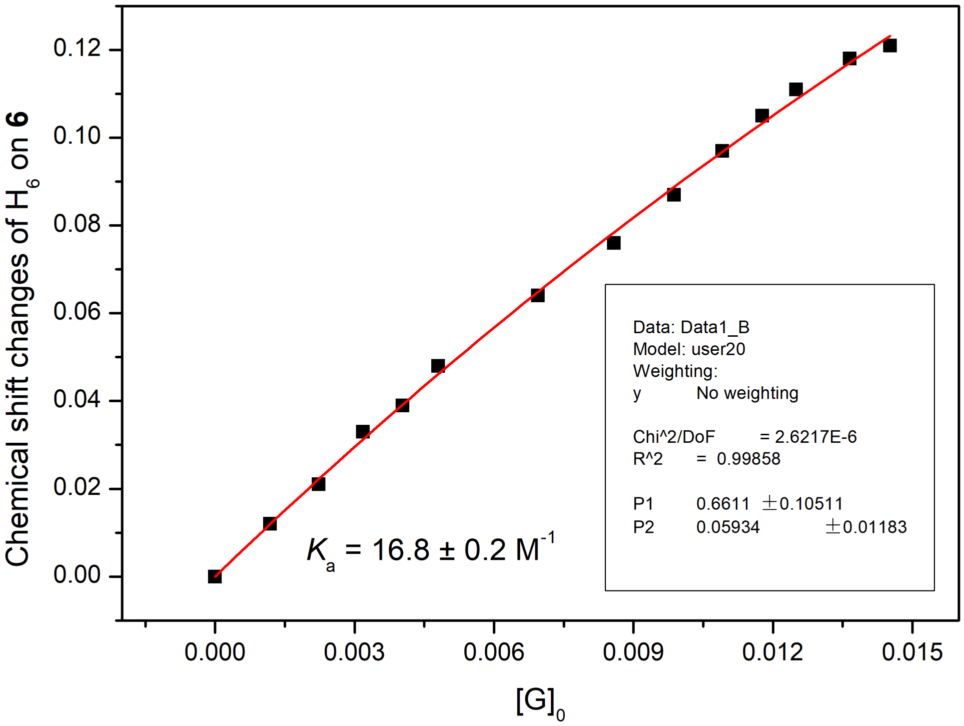

Fig. S25 Changes in the chemical shift corresponding to H_6 peak of **6** as a function of the added **PZDO**. The red solid line was obtained from a non-linear curve-fitting using **Eq. S1**.

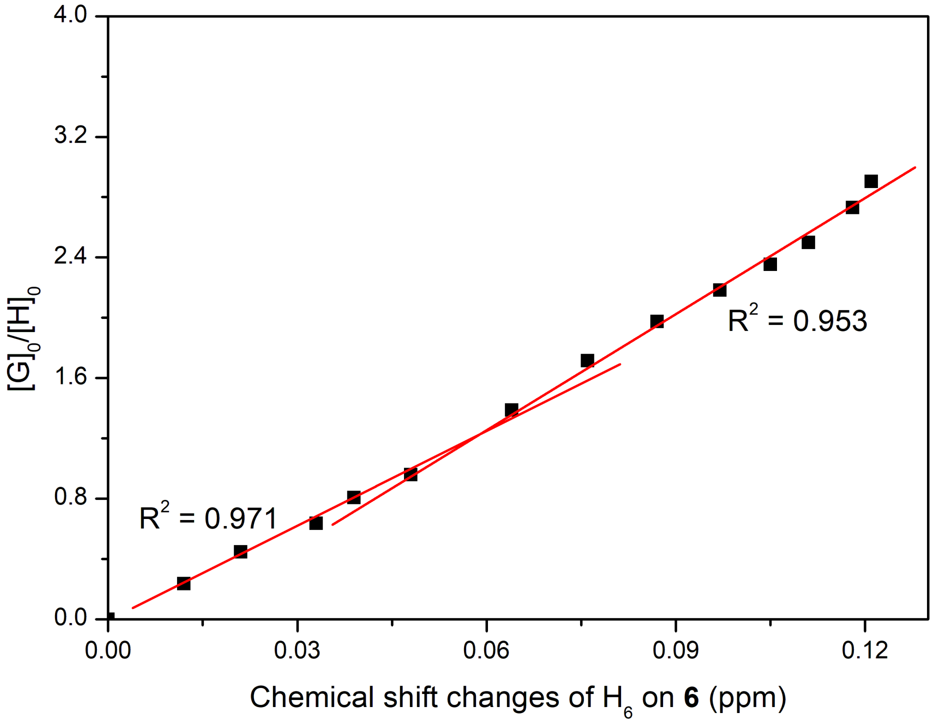

Fig. S26 Mole ratio plot for the complexation between **6** and **PZDO**. Although difficult to ascertain with certainty, the result is deemed consistent with a 1:1 binding stoichiometry.

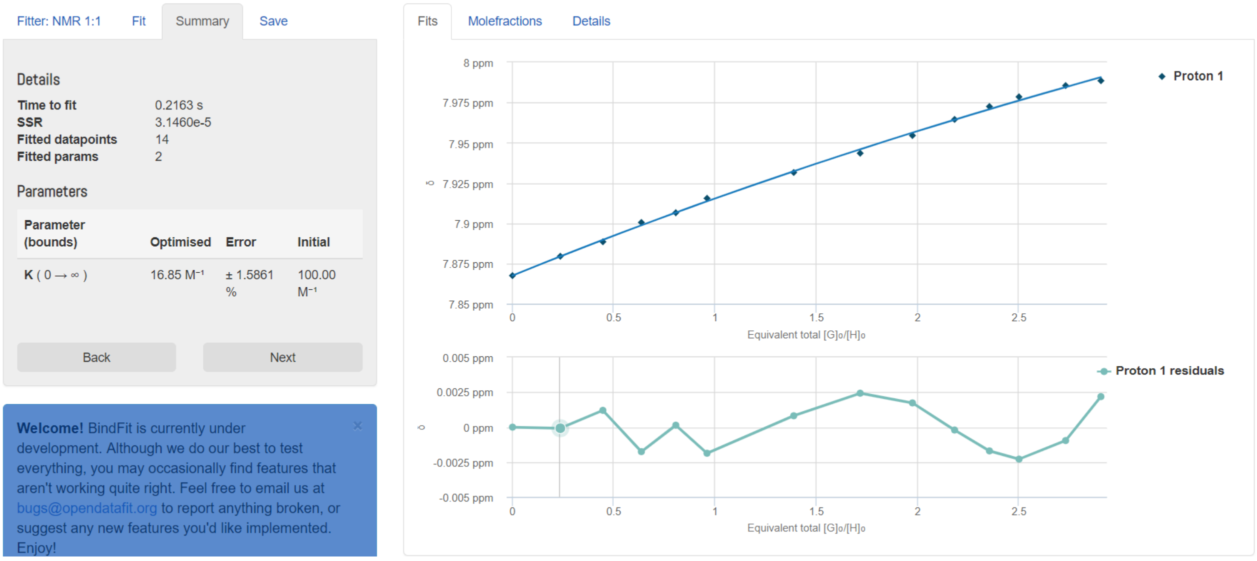

Fig. S27 Least-squares non-linear fitting of the changes in the chemical shift corresponding to the H_6 peak of **6** as a function of added **PZDO**. The solid lines were obtained from non-linear curve-fitting to a 1:1 binding model using the www.supramolecular.org web applet.

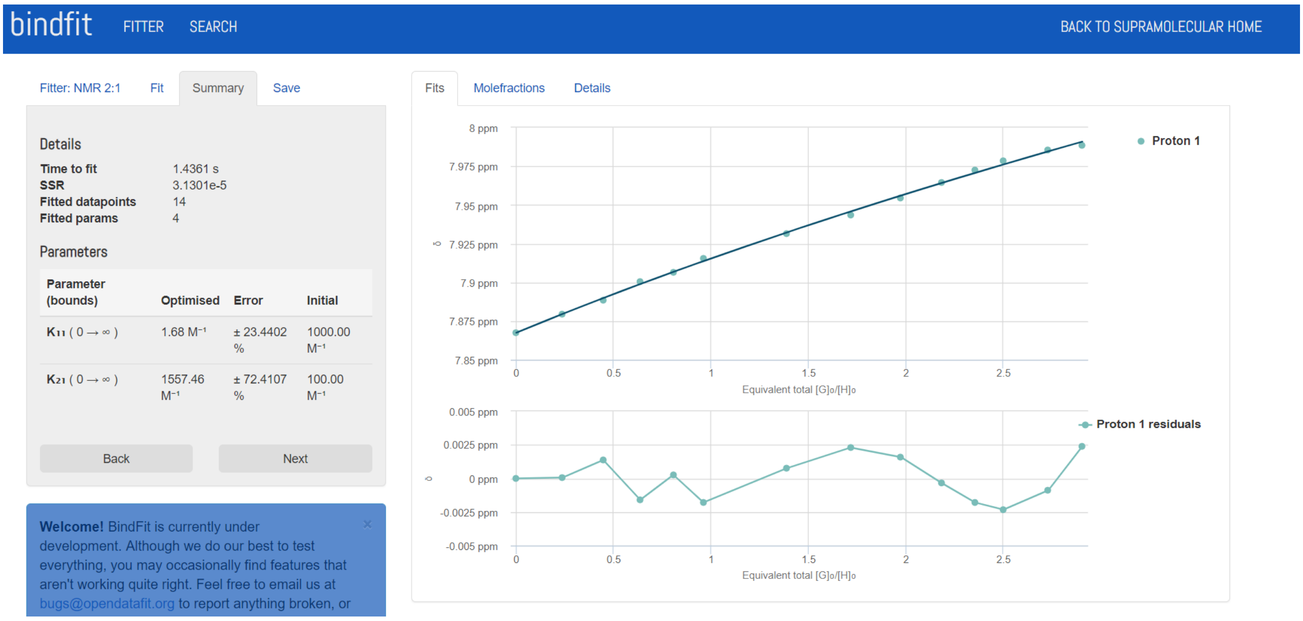

Fig. S28 Least-squares non-linear fitting of the changes in the chemical shift corresponding to the H_6 peak of **6** as a function of added **PZDO**. The solid lines were obtained from non-linear curve-fitting to a 2:1 binding model using the www.supramolecular.org web applet.

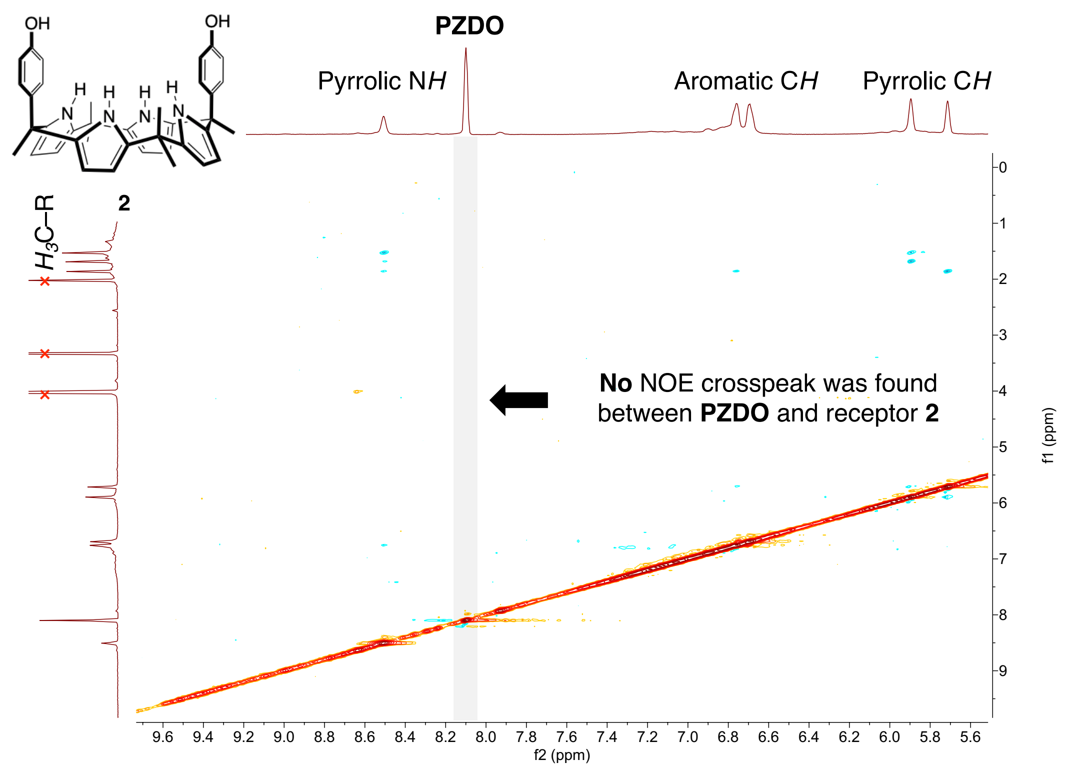

Fig. S29 Expanded view of the 2D NOESY NMR spectrum of receptor **2** (5.0 mM) with **PZDO** (5.0 mM) in a 1:1 (v/v) mixture of CD_3CN/CD_3OD at 298 K.

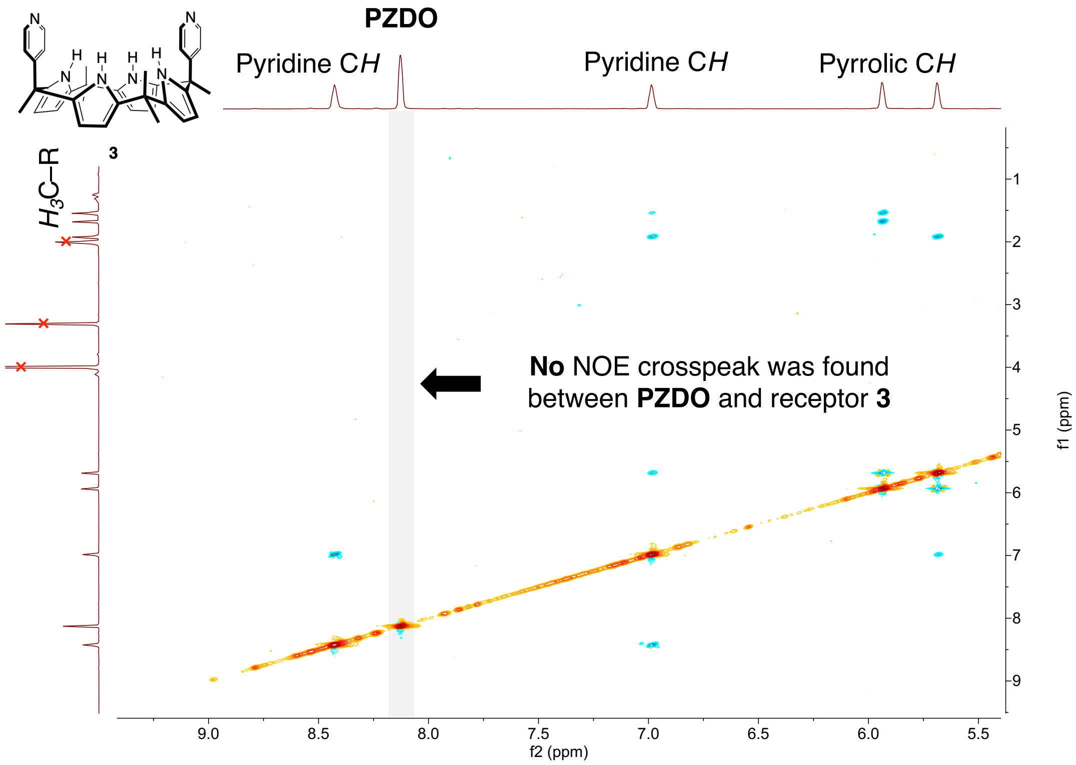

Fig. S30 Expanded view of the 2D NOESY NMR spectrum of receptor **3** (5.0 mM) with **PZDO** (5.0 mM) in a 1:1 (v/v) mixture of CD_3CN/CD_3OD at 298 K.

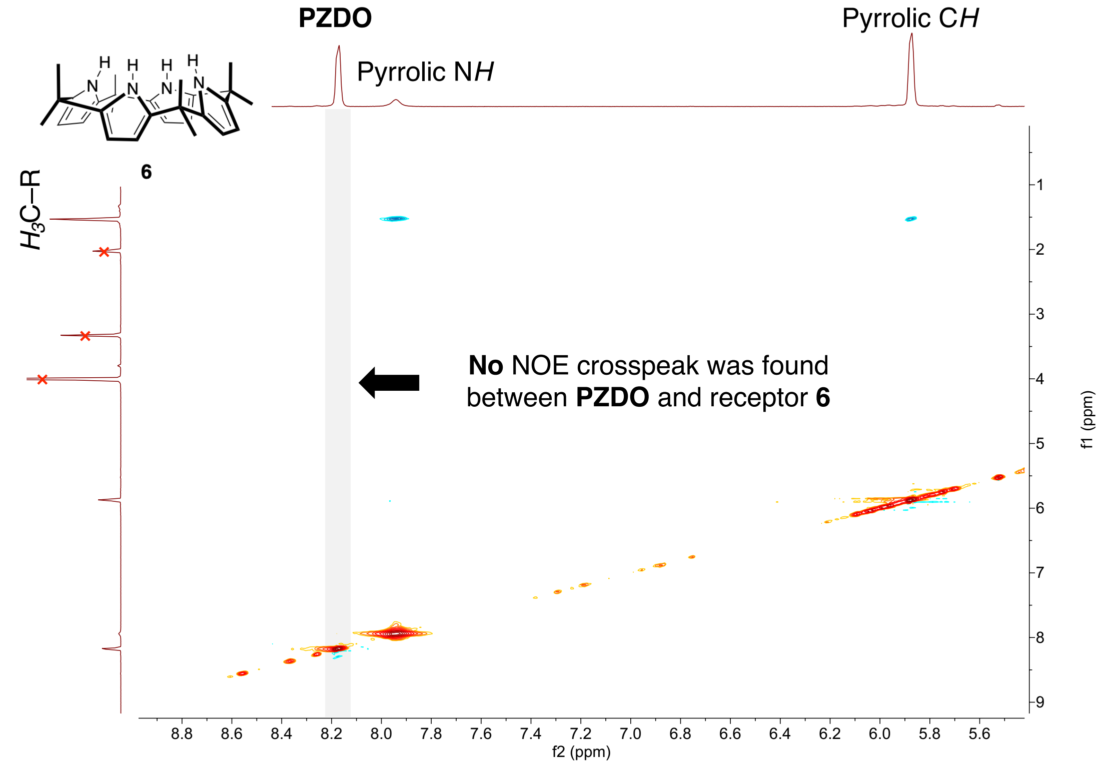

Fig. S31 Expanded view of the 2D NOESY NMR spectrum of receptor **6** (5.0 mM) with **PZDO** (5.0 mM) in a 1:1 (v/v) mixture of CD_3CN/CD_3OD at 298 K.

4. Computational analyses

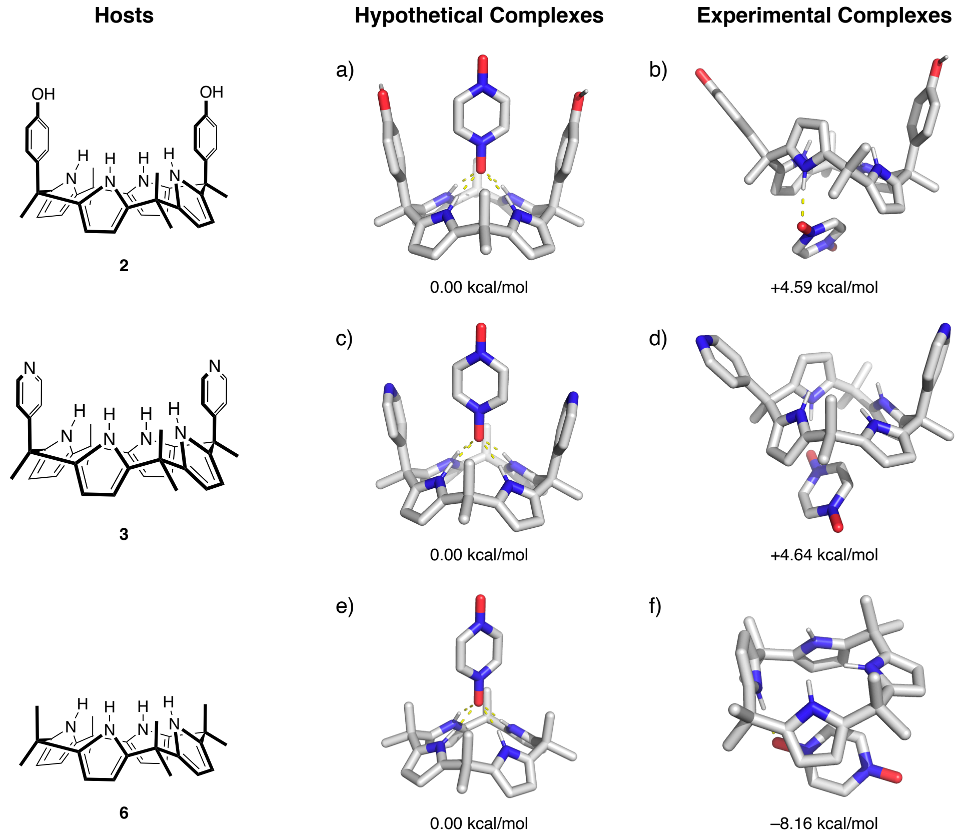

Fig. S32 DFT-optimised structures of the 1:1 host–guest complexes (a) (**2**·**PZDO**)$_{hyp}$, (b) (**2**·**PZDO**)$_{exp}$, (c) (**3**·**PZDO**)$_{hyp}$, (d) (**3**·**PZDO**)$_{exp}$, (e) (**6**·**PZDO**)$_{hyp}$ and (f) (**6**·**PZDO**)$_{exp}$, and their relative energies calculated at the CAM-B3LYP/6-31G(d)//CAM-B3LYP/6-31G(d) level in the gas phase. Intermolecular hydrogen bonds between the pyrrolic NH protons and **PZDO** are shown by yellow dashed lines. Non-polar hydrogen atoms are removed for clarity. All hypothetical complexes serve as the references for determining the relative energies compared to their respective experimental counterparts.

Total energy of the (**2**·**PZDO**)$_{hyp}$ complex: -2255.05734852 a.u.

Cartesian coordinates of the optimised complex (**2**·**PZDO**)$_{hyp}$:

Symbol	X	Y	Z	Symbol	X	Y	Z
C	2.277000	-2.730100	-3.166100	H	4.045200	-3.373300	2.322300
C	1.328400	-1.803800	-2.805600	H	-4.156500	-3.370600	2.142200
N	1.726600	-1.271500	-1.600900	H	-2.270700	-3.329500	4.064300
C	2.910800	-1.840500	-1.189400	H	-1.212500	-0.565300	1.087200
C	3.271400	-2.751600	-2.152600	H	1.013900	-1.539700	-5.481700
C	-2.123000	-2.731100	-3.260100	H	-0.762600	-1.539600	-5.520600
C	-3.164500	-2.748900	-2.295700	H	0.114100	-2.975200	-4.970700
C	-2.854200	-1.829600	-1.322900	H	-0.786500	0.559700	-4.178800
N	-1.652200	-1.260200	-1.680100	H	0.064000	0.679300	-2.634000
C	-1.195700	-1.798900	-2.861500	H	0.991500	0.555800	-4.134300
C	2.854000	-1.829800	1.322900	H	5.547700	-1.815400	1.016100
N	1.652000	-1.260300	1.680100	H	5.590900	-1.822000	-0.756500
C	1.195500	-1.799100	2.861400	H	4.890000	-3.197000	0.117600
C	2.122700	-2.731500	3.259900	H	-0.114200	-2.975500	4.970500
C	3.164200	-2.749300	2.295600	H	0.762400	-1.540000	5.520600
C	-2.911000	-1.840400	1.189400	H	-1.014100	-1.540100	5.481700
C	-3.271500	-2.751700	2.152400	H	0.786500	0.559400	4.179000
C	-2.277100	-2.730200	3.165900	H	-0.991500	0.555600	4.134600
C	-1.328600	-1.803800	2.805600	H	-0.064100	0.679300	2.634300
N	-1.726900	-1.271300	1.601000	H	-5.547900	-1.815000	-1.016100
C	0.082800	-1.331500	-3.533500	H	-5.591100	-1.821600	0.756500
C	0.113900	-1.882700	-4.964700	H	-4.890300	-3.196600	-0.117700
C	0.088400	0.210100	-3.621900	H	3.810300	0.337000	2.204000
C	3.625400	-1.417900	0.082000	H	4.112900	0.220700	-2.061000
C	5.002100	-2.110200	0.117100	H	4.663600	2.641900	-2.086300
C	-0.083000	-1.331600	3.533500	H	4.337200	2.744800	2.183000
C	-0.114100	-1.883000	4.964700	H	-3.810500	0.337300	-2.203900
C	-0.088400	0.209900	3.622100	H	-4.112800	0.221000	2.061100
C	-3.625600	-1.417700	-0.082100	H	-4.663300	2.642200	2.086400
C	-5.002300	-2.109800	-0.117100	H	-4.337200	2.745100	-2.183000
C	3.894500	0.101100	0.072100	C	-4.518200	2.845200	-0.039800
C	3.986800	0.834000	1.256000	C	4.518300	2.844800	0.039900
C	4.157200	0.772500	-1.127400	O	-4.746500	4.191700	0.042400
C	4.466500	2.126600	-1.152100	H	-4.882300	4.547200	-0.847900
C	4.289900	2.192400	1.247000	O	4.746700	4.191400	-0.042300
C	-3.894500	0.101400	-0.072100	H	4.882800	4.546800	0.848000
C	-3.986800	0.834300	-1.256000	H	2.020600	1.993000	-0.441000
C	-4.157100	0.772800	1.127500	C	1.136400	2.582400	-0.242800
C	-4.466300	2.126900	1.152100	C	-1.145300	3.950600	0.239700
C	-4.289900	2.192700	-1.247000	N	0.000100	1.897600	-0.000100
H	2.270600	-3.329200	-4.064600	C	1.145900	3.950400	-0.239700
H	1.212200	-0.565500	-1.087100	N	0.000300	4.662500	0.000200
H	4.156400	-3.370500	-2.142500	C	-1.136000	2.582500	0.242600
H	-2.070500	-3.337900	-4.151900	H	2.038200	4.536400	-0.413600
H	-4.045600	-3.372800	-2.322500	H	-2.020400	1.993300	0.440600
H	-1.176200	-0.534000	-1.156800	H	-2.037600	4.536700	0.413800
H	1.176100	-0.534100	1.156800	O	0.000500	5.915900	0.000400
H	2.070100	-3.338300	4.151700	O	0.000000	0.580000	-0.000000

Total energy of the (**2**·**PZDO**)$_{exp}$ complex: -2255.05002807 a.u.

Cartesian coordinates of the optimised complex (**2**·**PZDO**)$_{exp}$:

Symbol	X	Y	Z	Symbol	X	Y	Z
C	1.039600	4.836900	0.931700	C	-5.244000	0.852000	-0.203000
H	1.553200	5.759500	0.704800	H	-5.876600	0.305300	0.499100
C	1.416700	3.985400	1.933400	H	-5.721600	0.831600	-1.185000
H	2.260100	4.167400	2.583800	H	-5.174500	1.891900	0.127000
C	-0.332700	2.563000	1.390700	C	0.498600	2.459100	-4.050300
H	-0.872900	1.659000	1.633500	H	0.395600	3.446300	-3.592800
C	-0.712700	3.405900	0.380900	H	-0.216800	2.389300	-4.874000
H	-1.557200	3.213300	-0.267500	H	1.507900	2.368700	-4.459300
N	-0.011200	4.545000	0.112500	C	0.328800	-0.015100	-3.750500
N	0.753500	2.827200	2.157900	H	1.330100	-0.171100	-4.163500
O	-0.315000	5.300900	-0.853200	H	-0.396600	-0.046100	-4.568200
O	1.154400	2.011500	3.079200	H	0.098700	-0.857000	-3.088500
C	2.128900	-1.198300	1.201100	C	3.978500	0.395600	1.809900
C	1.721100	-2.472200	0.872400	H	3.303200	1.059500	2.356200
H	2.222900	-3.137700	0.185000	H	4.807000	0.988100	1.412300
C	0.566900	-2.766400	1.651600	H	4.393400	-0.332000	2.511100
H	0.027300	-3.702800	1.665900	C	-4.038300	-1.244400	-0.793200
C	0.298800	-1.662100	2.430000	C	-3.842800	-1.581400	-2.134800
C	-0.820200	-1.389400	3.412100	H	-3.498700	-0.824900	-2.831700
C	-1.841100	-0.476500	2.764200	C	-4.087000	-2.864400	-2.603100
C	-2.599900	0.561000	3.249900	H	-3.931200	-3.120600	-3.645400
H	-2.536000	0.980300	4.243600	C	-4.541300	-3.847900	-1.730100
C	-3.481800	0.977200	2.212000	C	-4.752500	-3.531900	-0.390800
H	-4.205300	1.777500	2.271900	H	-5.112800	-4.292200	0.298700
C	-3.243900	0.186900	1.112600	C	-4.504200	-2.243400	0.063900
C	-3.851800	0.196600	-0.277500	H	-4.679000	-2.012600	1.110500
C	-2.989400	1.005700	-1.228400	C	4.279700	-1.197800	-0.088800
C	-3.306900	2.068000	-2.040000	C	4.704800	-0.905300	-1.381000
H	-4.268600	2.556100	-2.097400	H	4.280400	-0.058800	-1.909500
C	-2.157200	2.389100	-2.813100	C	5.680200	-1.670800	-2.014500
H	-2.083800	3.177200	-3.547100	H	5.993800	-1.420300	-3.025700
C	-1.155100	1.517500	-2.463100	C	6.253500	-2.750700	-1.356000
C	0.243500	1.345100	-3.026100	C	5.846300	-3.058800	-0.059300
C	1.305800	1.428300	-1.948300	H	6.301100	-3.903400	0.447000
C	2.075600	2.488900	-1.531500	C	4.875600	-2.288200	0.557100
H	2.032400	3.492100	-1.931400	H	4.562500	-2.547100	1.563300
C	2.911900	2.034000	-0.474100	N	1.247900	-0.715500	2.136800
H	3.649600	2.617700	0.057600	H	1.254700	0.238300	2.513200
C	2.637500	0.705100	-0.264200	N	-2.230700	-0.678100	1.460700
C	3.250300	-0.329900	0.657000	H	-1.903200	-1.454000	0.901800
C	-0.278300	-0.734200	4.692500	N	-1.674600	0.685800	-1.494300
H	0.489900	-1.370200	5.141200	H	-1.142500	-0.003400	-0.985200
H	-1.083700	-0.609200	5.423000	N	1.657000	0.354700	-1.161100
H	0.152000	0.249800	4.491800	H	1.324000	-0.594700	-1.248800
C	-1.506900	-2.716300	3.787700	O	-4.759500	-5.092500	-2.237000
H	-1.941000	-3.209100	2.912700	H	-5.080200	-5.672100	-1.531700
H	-2.315800	-2.525000	4.498000	O	7.210700	-3.539600	-1.921500
H	-0.790800	-3.404100	4.247300	H	7.402300	-3.213300	-2.812000

Total energy of the (**3**·**PZDO**)$_{hyp}$ complex: -2136.74437482 a.u.

Cartesian coordinates of the optimised complex (**3**·**PZDO**)$_{hyp}$:

Symbol	X	Y	Z	Symbol	X	Y	Z
C	2.105300	-2.516300	-3.289700	H	1.218600	-0.359100	1.082600
C	1.178500	-1.589700	-2.877400	H	2.279900	-3.120600	4.061000
N	1.637200	-1.063700	-1.691300	H	4.145900	-3.191300	2.121400
C	2.841000	-1.637000	-1.345000	H	-4.030200	-3.157300	2.380000
C	3.149700	-2.544300	-2.329300	H	-2.049400	-3.094800	4.201200
C	-2.282700	-2.540300	-3.148900	H	-1.159800	-0.338500	1.161100
C	-3.266300	-2.573500	-2.125800	H	0.720900	-1.313300	-5.532600
C	-2.905700	-1.659100	-1.166000	H	-1.054700	-1.321000	-5.476600
N	-1.731800	-1.075900	-1.588700	H	-0.144300	-2.756100	-4.983500
C	-1.341100	-1.603400	-2.798000	H	-1.025800	0.767500	-4.116100
C	2.906400	-1.653400	1.172800	H	-0.083200	0.884800	-2.624600
N	1.732200	-1.068700	1.592700	H	0.750900	0.776700	-4.179000
C	1.341800	-1.590700	2.804500	H	5.584600	-1.660200	0.728900
C	2.283800	-2.525600	3.159800	H	5.536700	-1.645900	-1.044700
C	3.267400	-2.563200	2.136700	H	4.867600	-3.023700	-0.149700
C	-2.840300	-1.631700	1.351600	H	0.144800	-2.734100	4.994900
C	-3.148800	-2.534500	2.340100	H	1.055300	-1.296900	5.481800
C	-2.104400	-2.502000	3.300300	H	-0.720200	-1.288900	5.537900
C	-1.177800	-1.577000	2.883800	H	1.025600	0.785600	4.113700
N	-1.636700	-1.056400	1.695400	H	-0.751200	0.795100	4.174200
C	-0.105800	-1.120700	-3.536400	H	0.085100	0.896900	2.620500
C	-0.148500	-1.663700	-4.970400	H	-5.583900	-1.665000	-0.722100
C	-0.116800	0.421200	-3.614900	H	-5.536000	-1.643000	1.051400
C	3.617000	-1.240800	-0.103200	H	-4.866300	-3.024400	0.162400
C	4.991700	-1.938700	-0.144800	H	4.146700	0.409000	2.052200
C	0.106400	-1.105000	3.540800	H	3.809900	0.558700	-2.223600
C	0.149100	-1.641700	4.977100	H	4.345100	2.974700	-2.090600
C	0.117200	0.437200	3.612700	H	4.686700	2.828900	2.004300
C	-3.616400	-1.241200	0.108100	H	-4.146600	0.399400	-2.054300
C	-4.990900	-1.939500	0.152800	H	-3.810600	0.567000	2.220900
C	3.908900	0.270100	-0.087900	H	-4.347200	2.982200	2.077800
C	4.180100	0.940600	1.107200	H	-4.688000	2.819100	-2.016500
C	3.990600	1.022100	-1.260100	N	-4.520500	3.027200	0.026600
C	4.294100	2.376900	-1.183000	N	4.518800	3.028400	-0.039700
C	4.480800	2.296800	1.077900	H	-2.037900	2.209400	-0.347800
C	-3.909100	0.269500	0.086400	C	-1.146100	2.797800	-0.192100
C	-4.180500	0.934900	-1.111500	C	1.155900	4.165800	0.172900
C	-3.991400	1.026300	1.255500	N	-0.000500	2.111700	-0.004300
C	-4.295700	2.380700	1.172700	C	-1.157300	4.164900	-0.188800
C	-4.481900	2.291000	-1.087900	N	-0.000700	4.878600	-0.009300
H	2.050400	-3.113200	-4.187900	C	1.145000	2.798700	0.181100
H	1.160300	-0.343100	-1.160500	H	-2.060000	4.745400	-0.317000
H	4.031200	-3.167000	-2.366500	H	2.036800	2.210900	0.339200
H	-2.278500	-3.139500	-4.047300	H	2.058600	4.746900	0.298900
H	-4.144500	-3.201900	-2.107500	O	-0.000900	6.129700	-0.011600
H	-1.218500	-0.363500	-1.082000	O	-0.000200	0.791400	-0.001900

Total energy of the (**3**·**PZDO**)$_{exp}$ complex: -2136.73698024 a.u.

Cartesian coordinates of the optimised complex (**3**·**PZDO**)$_{exp}$:

Symbol	X	Y	Z	Symbol	X	Y	Z
C	2.957800	1.300900	-0.750100	H	0.647800	-5.089600	2.093000
C	3.288900	2.605900	-1.024000	H	2.158600	-4.423600	2.751200
H	4.246800	3.069300	-0.839300	C	5.177300	0.723400	0.204100
C	2.159500	3.228700	-1.622900	H	5.693300	1.140400	-0.663400
H	2.100700	4.253700	-1.956100	H	5.795400	-0.071900	0.624900
C	1.156600	2.294400	-1.707900	H	5.064900	1.512000	0.952000
C	-0.223700	2.382200	-2.332200	C	-4.339900	-1.135700	-0.882200
C	-1.313600	2.000100	-1.350800	C	-4.752800	-0.300400	-1.915500
C	-2.092100	2.781000	-0.528900	H	-4.335700	0.693700	-2.027400
H	-2.038300	3.857500	-0.448400	C	-5.724100	-0.746600	-2.806800
C	-2.954900	1.919600	0.204300	H	-6.052100	-0.099100	-3.617600
H	-3.705100	2.221300	0.921100	C	-5.907200	-2.740300	-1.748600
C	-2.686100	0.629700	-0.181900	H	-6.384600	-3.716800	-1.700300
C	-3.320000	-0.697000	0.180800	C	-4.948300	-2.390500	-0.809400
C	-2.216900	-1.732900	0.303800	H	-4.670000	-3.096800	-0.035100
C	-1.798800	-2.728100	-0.552000	C	4.061500	-0.894900	-1.300400
H	-2.281500	-3.013500	-1.475600	C	4.521200	-2.169100	-0.962800
C	-0.661100	-3.347500	0.037200	H	4.656300	-2.451700	0.076200
H	-0.120300	-4.196100	-0.357300	C	4.807600	-3.079700	-1.971000
C	-0.413400	-2.709500	1.232400	H	5.165700	-4.075600	-1.718700
C	0.678600	-2.915600	2.260100	C	4.239300	-1.598800	-3.589400
C	1.706000	-1.810400	2.125000	H	4.134800	-1.391300	-4.652300
C	2.433700	-1.103100	3.051000	C	3.925100	-0.614900	-2.657800
H	2.333200	-1.176100	4.124100	H	3.583800	0.358000	-2.992500
C	3.340900	-0.265000	2.342800	N	1.655200	1.127100	-1.170000
H	4.050700	0.421300	2.781700	N	-1.684100	0.694700	-1.120200
C	3.148800	-0.474600	0.998300	N	-1.358200	-1.724800	1.374300
C	3.803000	0.162600	-0.211800	N	2.137000	-1.403300	0.883500
C	-0.463600	3.826300	-2.792700	N	-6.302500	-1.942800	-2.742900
H	-0.386000	4.526200	-1.956900	N	4.670600	-2.817500	-3.271000
H	-1.459700	3.919000	-3.232100	H	-1.375800	-1.039200	2.137100
H	0.275100	4.112900	-3.546000	H	1.836600	-1.848800	0.027300
C	-0.274600	1.459900	-3.568700	H	1.107800	0.296300	-1.003100
H	0.471400	1.780300	-4.301100	H	-1.348100	-0.123500	-1.607600
H	-1.262700	1.495100	-4.037400	C	0.210500	1.538800	2.201900
H	-0.050900	0.416100	-3.322600	C	-1.553900	2.573300	3.294800
C	-4.078800	-0.547100	1.518300	N	-0.891500	1.434600	2.984500
H	-4.509000	-1.505000	1.818300	O	-1.308300	0.292500	3.429700
H	-4.896900	0.169700	1.406400	H	0.748700	0.619300	2.021300
H	-3.414200	-0.198900	2.313000	H	-2.409900	2.447700	3.942100
C	0.097700	-2.910000	3.683200	C	-1.160300	3.783200	2.792700
H	0.882300	-3.133300	4.412700	C	0.607500	2.744100	1.688400
H	-0.676200	-3.677200	3.773400	N	-0.092200	3.886200	1.950000
H	-0.336000	-1.940200	3.938900	O	0.229000	4.993900	1.436100
C	1.368200	-4.270400	2.011600	H	-1.671600	4.712200	2.998100
H	1.826500	-4.316700	1.019200	H	1.465100	2.860800	1.039200

Total energy of the (**6·PZDO**)$_{hyp}$ complex: -1721.39721331 a.u.

Cartesian coordinates of the optimised complex (**6·PZDO**)$_{hyp}$:

Symbol	X	Y	Z	Symbol	X	Y	Z
C	0.718700	-3.641500	-2.268300	H	-1.337100	4.167900	-2.975400
C	1.138100	-2.853700	-1.224600	H	-0.000500	1.751700	0.184100
N	0.002900	-2.379500	-0.604100	H	3.225800	-4.561100	-1.054800
C	-1.128100	-2.854100	-1.232300	H	4.529300	-3.356900	-0.957300
C	-0.701300	-3.641700	-2.273100	H	3.538900	-3.475700	-2.418200
C	3.991800	-0.712300	-1.940300	H	3.596600	-2.471500	1.173900
C	3.991600	0.708600	-1.942000	H	1.898300	-1.993200	1.309800
C	2.977100	1.131400	-1.119300	H	2.309600	-3.698800	1.103800
N	2.375500	-0.000500	-0.613500	H	-4.520800	-3.358300	-0.987700
C	2.977400	-1.133500	-1.116500	H	-3.216200	-4.562000	-1.076700
C	-2.968600	-1.134300	-1.136500	H	-3.520600	-3.476600	-2.442000
N	-2.370300	-0.001200	-0.629600	H	-3.521600	3.469400	-2.450800
C	-2.968900	1.130600	-1.139300	H	-4.521800	3.354500	-0.996200
C	-3.978100	0.707400	-1.968400	H	-3.217600	4.558300	-1.088200
C	-3.977900	-0.713600	-1.966600	H	-3.603500	2.474200	1.143400
C	1.137300	2.851000	-1.231600	H	-2.316300	3.701700	1.078800
C	0.717700	3.636200	-2.277100	H	-1.906000	1.996700	1.291900
C	-0.702300	3.636000	-2.281900	H	4.528400	3.355600	-0.965300
C	-1.128900	2.850600	-1.239400	H	3.224600	4.559200	-1.065700
N	0.002200	2.377800	-0.610000	H	3.538000	3.470700	-2.426500
C	2.535400	-2.531500	-0.726500	C	2.585500	2.684300	0.802600
C	3.517900	-3.543900	-1.328100	H	3.595700	2.474700	1.168000
C	2.586300	-2.681900	0.809100	H	2.308800	3.701900	1.094900
C	-2.528700	-2.532300	-0.743500	H	1.897400	1.996800	1.304800
C	-3.506900	-3.544900	-1.351800	C	-2.590000	-2.682700	0.791600
C	-2.529500	2.529600	-0.749900	H	-3.602600	-2.472300	1.149700
C	-3.507900	3.540500	-1.360800	H	-2.315200	-3.699700	1.088100
C	-2.590800	2.684000	0.784900	H	-1.905200	-1.994100	1.296900
C	2.534600	2.530300	-0.732600	H	2.055700	0.003700	2.724200
C	3.516900	3.541500	-1.336600	C	1.149400	0.004300	3.314300
H	1.358100	-4.175000	-2.956200	C	-1.186800	0.006000	4.672000
H	0.000100	-1.751600	0.188500	N	-0.009500	0.003300	2.617500
H	-1.336000	-4.175300	-2.965300	C	1.150600	0.006100	4.681900
H	4.670900	-1.349900	-2.487000	N	-0.021100	0.006900	5.390600
H	4.670500	1.345000	-2.490400	C	-1.174100	0.004300	3.304500
H	1.556700	0.000100	-0.020500	H	2.049200	0.006900	5.281500
H	-1.554800	-0.000300	-0.032100	H	-2.075400	0.003500	2.706800
H	-4.653700	1.343600	-2.521100	H	-2.090400	0.006800	5.264100
H	-4.653400	-1.351300	-2.517600	O	-0.026400	0.008500	6.642600
H	1.357000	4.168300	-2.966200	O	-0.003900	0.001600	1.308400

Total energy of the (**6**·**PZDO**)$_{exp}$ complex: -1721.41022430 a.u.

Cartesian coordinates of the optimised complex (**6**·**PZDO**)$_{exp}$:

Symbol	X	Y	Z	Symbol	X	Y	Z
C	3.242000	-0.417200	-0.587500	C	-0.412800	5.037600	-0.196200
C	3.433000	-0.601100	-1.939500	H	-1.156200	5.527600	-0.830700
H	4.104400	-0.035700	-2.570300	H	0.495600	5.645000	-0.190700
C	2.628500	-1.705400	-2.339700	H	-0.807600	5.003600	0.822200
H	2.577700	-2.135200	-3.330000	C	4.286300	-0.015800	1.662800
C	1.968800	-2.163900	-1.221700	H	3.472900	-0.494600	2.213300
C	1.000300	-3.312000	-1.035900	H	4.735300	0.742400	2.312200
C	-0.379000	-2.774100	-0.717900	H	5.050700	-0.765600	1.440100
C	-1.341600	-3.181800	0.170700	C	4.964600	1.364900	-0.309800
H	-1.243600	-3.992200	0.878700	H	5.766700	0.663400	-0.558600
C	-2.489200	-2.362500	-0.023200	H	5.360700	2.123700	0.370200
H	-3.420500	-2.420700	0.521700	H	4.654400	1.870600	-1.228800
C	-2.208300	-1.467500	-1.026600	N	2.341500	-1.364500	-0.169900
C	-3.061800	-0.375300	-1.638200	N	-0.915700	-1.727800	-1.433600
C	-2.695400	1.003500	-1.119800	N	-1.408300	1.499900	-1.164400
C	-3.491800	2.015000	-0.643500	N	1.837200	2.047300	-0.323600
H	-4.559600	1.963400	-0.493600	H	1.911000	1.714000	-1.274100
C	-2.663800	3.143900	-0.389600	H	-0.433900	-1.297500	-2.209000
H	-2.993600	4.107100	-0.028700	H	1.981300	-1.450100	0.786800
C	-1.373600	2.804800	-0.715600	H	-0.589000	0.930600	-1.315500
C	-0.101700	3.631600	-0.725400	C	0.400900	3.765900	-2.178100
C	0.969200	3.006800	0.146800	H	-0.355300	4.270500	-2.785300
C	1.271900	3.189000	1.476300	H	0.587000	2.794200	-2.648100
H	0.771000	3.868500	2.151100	H	1.326900	4.347800	-2.216100
C	2.353300	2.323100	1.804400	C	0.932400	-4.143400	-2.329900
H	2.828400	2.227300	2.770100	H	0.583800	-3.545500	-3.177300
C	2.691200	1.627400	0.668700	H	0.234500	-4.974500	-2.198500
C	3.788700	0.629700	0.359500	H	1.917200	-4.548700	-2.581000
C	1.474900	-4.221600	0.110100	C	-0.353500	0.022500	2.021300
H	1.496300	-3.687000	1.063500	C	-0.867900	-1.864100	3.267500
H	2.480900	-4.593400	-0.103200	N	0.052700	-1.139400	2.590100
H	0.808700	-5.083900	0.214000	O	1.274700	-1.553700	2.499800
C	-4.530500	-0.650400	-1.288500	H	0.418700	0.610900	1.545500
H	-4.817000	-1.649500	-1.626000	H	-0.495700	-2.768100	3.727400
H	-5.180400	0.077900	-1.780600	C	-2.176300	-1.471000	3.328300
H	-4.696100	-0.581300	-0.210000	C	-1.663600	0.418100	2.067700
C	-2.908900	-0.390200	-3.174700	N	-2.606900	-0.342600	2.694700
H	-1.887600	-0.151800	-3.491300	O	-3.829500	-0.021700	2.693300
H	-3.565200	0.361800	-3.621300	H	-2.946700	-2.030700	3.838100
H	-3.171300	-1.372600	-3.579100	H	-2.028000	1.321900	1.597300

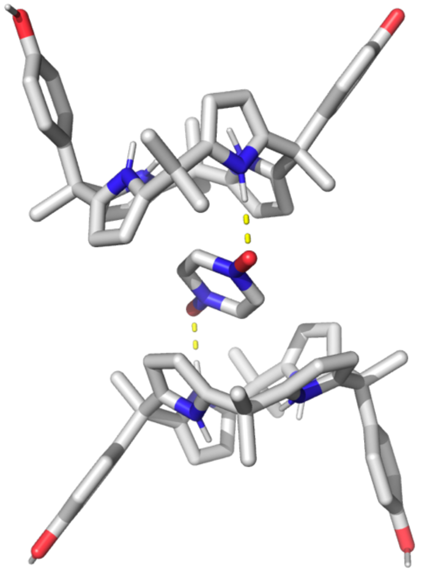

Fig. S33 Optimised structure of the (**2**$_2$·**PZDO**)$_{exp}$ complex. The intermolecular hydrogen bonds between the pyrrolic NH protons and **PZDO** are shown by yellow dashed lines. Non-polar hydrogen atoms are removed for clarity.

Total energy of the (**2**$_2$·**PZDO**)$_{exp}$ complex: -4095.65043753 a.u.

Cartesian coordinates of the optimised complex (**2**$_2$·**PZDO**)$_{exp}$:

Symbol	X	Y	Z	Symbol	X	Y	Z
C	1.135500	0.549600	0.547000	H	6.529000	-2.722000	0.958300
H	2.023800	0.948000	1.014000	C	5.995000	-0.976100	2.261500
C	0.633300	1.031300	-0.632600	H	6.954600	-0.554000	2.524900
H	1.102600	1.825900	-1.197200	C	4.766700	-0.543900	2.710300
C	-1.131400	-0.474600	-0.489900	C	4.385700	0.637300	3.577100
H	-2.020900	-0.872000	-0.955300	C	2.574500	-0.498800	-4.650100
C	-0.629300	-0.956500	0.689600	H	1.519900	-0.260500	-4.490700
H	-1.098100	-1.752000	1.253500	H	2.647400	-1.528300	-5.009100
N	0.511700	-0.451200	1.214000	H	2.960000	0.167600	-5.426500
N	-0.504500	0.521800	-1.159900	C	4.867200	-0.615000	-3.668300
O	0.981500	-0.913900	2.322300	H	5.214900	0.063200	-4.452500
O	-0.966800	0.975800	-2.275200	H	5.003300	-1.645600	-4.009900
C	3.810800	1.733900	2.703700	H	5.514600	-0.455500	-2.799100
C	2.783500	2.627500	2.889600	C	3.751600	5.158400	-0.523100
H	2.112400	2.653200	3.736100	H	4.156800	5.747800	0.301800
C	2.769700	3.512300	1.773800	H	2.660500	5.220000	-0.492700
H	2.079700	4.328800	1.615800	H	4.097300	5.597100	-1.461700
C	3.787800	3.141700	0.927500	C	3.352300	0.226200	4.638000
C	4.230400	3.698400	-0.412800	H	3.745900	-0.594200	5.244700
C	3.617100	2.903300	-1.550600	H	2.411500	-0.092400	4.182700
C	2.880500	3.316500	-2.634600	H	3.139000	1.068100	5.304000
H	2.558300	4.328100	-2.833000	C	5.640500	1.175700	4.290300
C	2.644900	2.184700	-3.463000	H	6.398100	1.513700	3.577100
H	2.095400	2.179700	-4.392200	H	6.086800	0.401500	4.921600
C	3.241900	1.097400	-2.873600	H	5.370600	2.028700	4.918700
C	3.381900	-0.334800	-3.355500	C	2.489300	-3.963200	1.572000
C	2.866900	-1.329800	-2.334800	H	1.795100	-3.191400	1.914900
C	1.635100	-1.928200	-2.208800	H	2.941700	-4.438300	2.445500
H	0.784900	-1.769000	-2.857200	H	1.922900	-4.725900	1.030600
C	1.672900	-2.771700	-1.063800	C	5.769200	3.699700	-0.508700
H	0.865600	-3.390900	-0.699800	C	6.542100	4.094200	0.590200
C	2.925800	-2.672200	-0.510900	H	6.049700	4.343300	1.525600
C	3.586300	-3.376000	0.656900	C	7.922900	4.176000	0.516900
C	4.417200	-2.352200	1.412300	H	8.515800	4.480600	1.372500
C	5.774200	-2.116400	1.438500	C	8.571300	3.864600	-0.676400

C	7.822900	3.479700	-1.782900
H	8.320700	3.239100	-2.719700
C	6.436800	3.401700	-1.694600
H	5.870500	3.109300	-2.572100
C	4.478800	-4.534300	0.175500
C	5.274500	-5.229500	1.088700
H	5.302500	-4.910900	2.125600
C	6.047700	-6.314300	0.700100
H	6.661000	-6.835700	1.432000
C	6.037700	-6.734300	-0.627700
C	5.248300	-6.060900	-1.552000
H	5.243800	-6.398200	-2.582900
C	4.480200	-4.976700	-1.147600
H	3.860900	-4.472700	-1.881500
N	4.397100	2.047000	1.499400
H	5.250400	1.614100	1.173700
N	3.825400	1.549100	-1.709100
H	4.260000	0.955100	-1.019200
N	3.635300	-1.788200	-1.288400
H	4.612500	-1.592800	-1.125200
N	3.819600	-1.382000	2.177800
H	2.807300	-1.255800	2.279200
O	9.929700	3.953800	-0.697300
H	10.250000	3.722900	-1.580700
O	6.777100	-7.790800	-1.071000
H	7.267800	-8.166600	-0.326700
C	-4.438200	2.349900	-1.422400
C	-5.789900	2.087900	-1.466400
H	-6.563300	2.681800	-1.001100
C	-5.976800	0.939600	-2.286600
H	-6.924400	0.498500	-2.562000
C	-4.734000	0.529100	-2.716000
C	-4.317400	-0.647200	-3.573000
C	-3.730300	-1.729000	-2.689100
C	-2.683500	-2.602500	-2.860200
H	-2.002300	-2.618000	-3.698800
C	-2.665900	-3.484600	-1.742100
H	-1.962200	-4.287500	-1.574700
C	-3.701400	-3.132700	-0.909100
C	-4.150100	-3.696300	0.426400
C	-3.576800	-2.881300	1.571000
C	-2.844100	-3.270000	2.666800
H	-2.494500	-4.271000	2.872300
C	-2.655300	-2.130000	3.496200
H	-2.120100	-2.106700	4.433400
C	-3.276200	-1.062600	2.895500
C	-3.464200	0.367200	3.367600
C	-2.943800	1.368500	2.355500
C	-1.718500	1.984600	2.250600
H	-0.879200	1.841200	2.916700
C	-1.746700	2.822900	1.101300
H	-0.942400	3.453300	0.750000
C	-2.987300	2.702500	0.525000
C	-3.637900	3.391000	-0.657600
C	-3.280000	-0.218100	-4.622900
H	-3.683200	0.591900	-5.237100
H	-3.040700	-1.057500	-5.283200
H	-2.351900	0.121700	-4.157000
C	-5.551600	-1.214300	-4.299600
H	-6.310700	-1.564900	-3.593900
H	-5.256300	-2.064000	-4.921100
H	-6.006100	-0.451900	-4.939300
C	-3.633600	-5.142300	0.550200
H	-4.008600	-5.744900	-0.279400
H	-3.983300	-5.587200	1.484300
H	-2.540900	-5.174300	0.538600
C	-2.698500	0.556200	4.683800
H	-1.633500	0.347000	4.556000
H	-3.087600	-0.118600	5.451100
H	-2.809200	1.584300	5.037200
C	-4.963800	0.612900	3.637500
H	-5.133800	1.641600	3.969700
H	-5.317000	-0.069400	4.415600
H	-5.583800	0.433700	2.752500
C	-2.535200	3.993600	-1.555500
H	-1.822700	3.232600	-1.884400
H	-1.990900	4.766800	-1.006600
H	-2.980300	4.459200	-2.437800
C	-5.689900	-3.739200	0.499200
C	-6.383100	-3.471800	1.682200
H	-5.833600	-3.173800	2.568600
C	-7.763900	-3.586300	1.755100
H	-8.296900	-3.375100	2.675800
C	-8.488500	-3.979400	0.634300
C	-7.817400	-4.260000	-0.552800
H	-8.374300	-4.573100	-1.433200
C	-6.435000	-4.141400	-0.611100
H	-5.926800	-4.368300	-1.543500
C	-4.558100	4.535500	-0.197200
C	-4.588800	4.982400	1.120200
H	-3.975100	4.494100	1.869100
C	-5.384300	6.059200	1.502600
H	-5.389800	6.387800	2.539700
C	-6.167500	6.714500	0.561400
C	-6.148000	6.287200	-0.764900
H	-6.761800	6.806700	-1.492800
C	-5.351600	5.215200	-1.129600
H	-5.354300	4.889100	-2.164600
N	-3.811200	1.388100	-2.174500
H	-2.795200	1.281500	-2.261300
N	-4.325000	-2.051100	-1.491100
H	-5.190500	-1.634100	-1.177000
N	-3.827600	-1.533700	1.723300
H	-4.276800	-0.955400	1.029600
N	-3.698200	1.811400	1.292800
H	-4.668200	1.598600	1.109900
O	-9.841600	-4.070300	0.755200
H	-10.217900	-4.360800	-0.087600
O	-6.968700	7.772800	0.872400
H	-6.881400	7.967100	1.816200

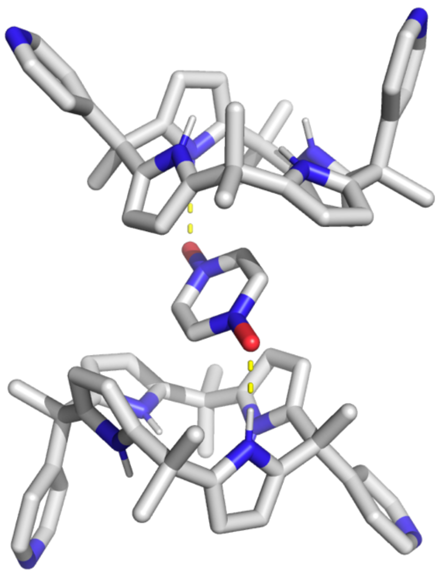

Fig. S34 Optimised structure of the (**3**$_2$·**PZDO**)$_{exp}$ complex. The intermolecular hydrogen bonds between the pyrrolic NH protons and **PZDO** are shown by yellow dashed lines. Non-polar hydrogen atoms are removed for clarity.

Total energy of the (**3**$_2$·**PZDO**)$_{exp}$ complex: -3859.02428886 a.u.

Cartesian coordinates of the optimised complex (**3**$_2$·**PZDO**)$_{exp}$:

**Symbol**	**X**	**Y**	**Z**	**Symbol**	**X**	**Y**	**Z**
C	-3.674400	-2.790300	-1.556500	H	-5.490900	0.578500	-2.885100
C	-2.947200	-3.253500	-2.626500	C	-2.346200	4.118500	1.368900
H	-2.648200	-4.277600	-2.794300	H	-2.786800	4.637100	2.223200
C	-2.687200	-2.153500	-3.489600	H	-1.740100	4.838900	0.812900
H	-2.139500	-2.190000	-4.419100	H	-1.692000	3.325900	1.740300
C	-3.261100	-1.035900	-2.935300	C	-3.462900	0.079200	4.556500
C	-3.367000	0.384900	-3.457800	H	-3.296100	-0.747500	5.253900
C	-2.812400	1.389600	-2.467500	H	-3.846700	0.930100	5.125900
C	-1.555500	1.936200	-2.351400	H	-2.499500	0.353600	4.119800
H	-0.710500	1.720700	-2.990100	C	-5.770100	-0.807600	4.170300
C	-1.561600	2.814400	-1.232200	H	-6.519500	-1.140600	3.446000
H	-0.730100	3.408900	-0.881800	H	-6.206900	-0.001000	4.766400
C	-2.820700	2.786100	-0.684700	H	-5.546900	-1.650100	4.830200
C	-3.457000	3.549300	0.458400	C	-3.850300	-5.008500	-0.452000
C	-4.346600	2.590500	1.230100	H	-4.189900	-5.476900	-1.378200
C	-5.712400	2.410800	1.232800	H	-4.269600	-5.562900	0.389600
H	-6.434200	3.031900	0.722100	H	-2.760600	-5.077900	-0.408500
C	-5.995700	1.303000	2.080500	C	-4.283700	4.737900	-0.057700
H	-6.977200	0.929100	2.335500	C	-4.245900	5.157900	-1.383600
C	-4.795200	0.834500	2.566700	H	-3.648700	4.629100	-2.117500
C	-4.478700	-0.331700	3.478500	C	-4.975400	6.280100	-1.764900
C	-3.914600	-1.472400	2.656100	H	-4.951000	6.614700	-2.800100
C	-2.918600	-2.387100	2.898400	C	-5.756400	6.597200	0.336900
H	-2.270500	-2.403000	3.762700	H	-6.369900	7.190600	1.011500
C	-2.903200	-3.311200	1.815300	C	-5.066000	5.494300	0.817400
H	-2.234800	-4.153000	1.705100	H	-5.148100	5.221200	1.863600
C	-3.888300	-2.942600	0.930700	C	-5.843500	-3.541300	-0.521500
C	-4.308600	-3.537800	-0.399000	C	-6.645600	-3.869400	0.573000
C	-2.570100	0.491500	-4.764800	H	-6.202600	-4.079400	1.541200
H	-1.519400	0.233500	-4.611000	C	-8.023800	-3.927000	0.414900
H	-2.622800	1.511500	-5.153100	H	-8.658200	-4.181200	1.261400
H	-2.980000	-0.187400	-5.517400	C	-7.882500	-3.378700	-1.779400
C	-4.847100	0.697600	-3.763400	H	-8.401800	-3.186500	-2.715900
H	-5.221200	0.009500	-4.526300	C	-6.495000	-3.294900	-1.727300
H	-4.958600	1.721900	-4.131500	H	-5.934200	-3.045600	-2.621000

N	-3.855100	-1.437400	-1.757900
N	-3.564900	1.912100	-1.440600
N	-3.804100	1.618800	2.032700
N	-4.480100	-1.810000	1.447700
N	-5.725300	7.000000	-0.934900
N	-8.651200	-3.683800	-0.736200
H	-2.799900	1.455700	2.159900
H	-5.313300	-1.362600	1.090700
H	-4.269600	-0.809800	-1.085500
H	-4.549800	1.760500	-1.276900
C	-1.169900	-0.444700	0.500100
C	0.659000	0.982100	0.651400
N	-0.517200	0.543500	1.158000
O	-0.992200	1.056300	2.241500
H	-2.088800	-0.787200	0.951800
H	1.149100	1.770300	1.207500
C	1.169900	0.444100	-0.499800
C	-0.659000	-0.982700	-0.651100
N	0.517200	-0.544100	-1.157700
O	0.992200	-1.056800	-2.241300
H	2.088800	0.786700	-0.951600
H	-1.149100	-1.770900	-1.207200
C	3.674100	2.790400	1.556400
C	2.946900	3.253300	2.626500
H	2.647700	4.277400	2.794300
C	2.687300	2.153300	3.489700
H	2.139700	2.189800	4.419300
C	3.261400	1.035800	2.935400
C	3.367700	-0.384900	3.457900
C	2.813000	-1.389600	2.467800
C	1.556100	-1.936300	2.351800
H	0.711200	-1.720900	2.990700
C	1.562100	-2.814600	1.232700
H	0.730500	-3.409200	0.882400
C	2.821100	-2.786200	0.685000
C	3.457300	-3.549400	-0.458200
C	4.346700	-2.590600	-1.230000
C	5.712500	-2.410700	-1.232700
H	6.434400	-3.031600	-0.722000
C	5.995600	-1.302900	-2.080500
H	6.977000	-0.928900	-2.335600
C	4.795100	-0.834600	-2.566700
C	4.478300	0.331500	-3.478500
C	3.914100	1.472300	-2.656100
C	2.918000	2.386800	-2.898500
H	2.269800	2.402500	-3.762700
C	2.902500	3.310900	-1.815300
H	2.233900	4.152600	-1.705100
C	3.887700	2.942500	-0.930800
C	4.308000	3.537900	0.398800
C	2.571200	-0.491600	4.765100
H	1.520400	-0.233800	4.611600
H	2.624200	-1.511500	5.153400
H	2.981200	0.187400	5.517600
C	4.847900	-0.697300	3.763100
H	5.222200	-0.009200	4.525800
H	4.959700	-1.721600	4.131200
H	5.491500	-0.578100	2.884700
C	2.346400	-4.118700	-1.368500
H	2.786900	-4.637200	-2.222900
H	1.740400	-4.839100	-0.812500
H	1.692100	-3.326100	-1.739900
C	3.462600	-0.079500	-4.556400
H	3.295600	0.747100	-5.253900
H	3.846400	-0.930400	-5.125800
H	2.499300	-0.354000	-4.119700
C	5.769700	0.807600	-4.170300
H	6.519100	1.140700	-3.446200
H	6.206600	0.001000	-4.766500
H	5.546300	1.650000	-4.830300
C	3.849400	5.008500	0.451800
H	4.189000	5.477000	1.377900
H	4.268500	5.563000	-0.389800
H	2.759700	5.077600	0.408400
C	4.284100	-4.737900	0.057800
C	4.246400	-5.158100	1.383700
H	3.649200	-4.629300	2.117700
C	4.976000	-6.280200	1.764900
H	4.951800	-6.614900	2.800100
C	5.756900	-6.597100	-0.337000
H	6.370500	-7.190400	-1.011600
C	5.066400	-5.494200	-0.817400
H	5.148500	-5.221100	-1.863600
C	5.842900	3.541800	0.521100
C	6.644900	3.870000	-0.573500
H	6.201700	4.079800	-1.541600
C	8.023100	3.927800	-0.415500
H	8.657300	4.182100	-1.262100
C	7.882100	3.379800	1.778900
H	8.401500	3.187700	2.715400
C	6.494600	3.295600	1.726900
H	5.934000	3.046300	2.620600
N	3.855100	1.437500	1.757800
N	3.565300	-1.912200	1.440800
N	3.804000	-1.619000	-2.032600
N	4.479600	1.810000	-1.447800
N	5.726000	-7.000000	0.934800
N	8.650600	3.685000	0.735500
H	2.799800	-1.456100	-2.159800
H	5.312900	1.362800	-1.090800
H	4.269900	0.810000	1.085600
H	4.550200	-1.760400	1.276800

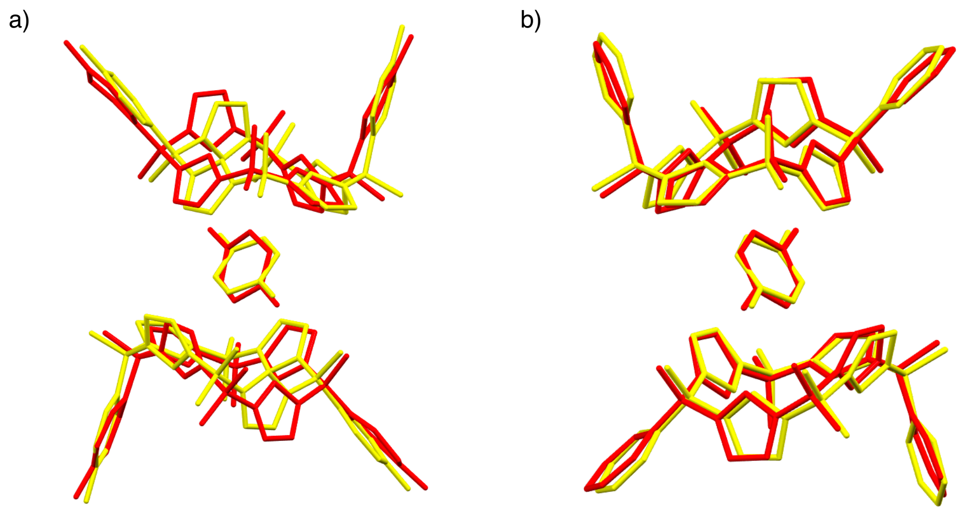

Fig. S35 (a) Overlay of the observed **2**$_2$·**PZDO** complex and the DFT-optimised **2**$_2$·**PZDO** complex. (b) Overlay of the observed **3**$_2$·**PZDO** complex and the DFT-optimised **3**$_2$·**PZDO** complex. In both cases, the original molecules are rendered with red whereas the optimised molecules are rendered with yellow. Both observed 2:1 host–guest complexes are extracted from their corresponding crystal structures. All hydrogen atoms are removed for clarity.

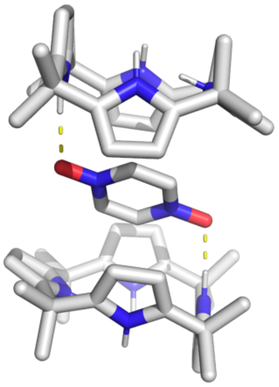

Fig. S36 Optimised structure of the (**6**$_2$·**PZDO**)$_{exp}$ complex. The intermolecular hydrogen bonds between the pyrrolic NH protons and **PZDO** are shown by yellow dashed lines. Non-polar hydrogen atoms are removed for clarity.

Total energy of the (**6**$_2$·**PZDO**)$_{exp}$ complex: -3028.37072468 a.u.

Cartesian coordinates of the optimised complex (**6**$_2$·**PZDO**)$_{exp}$:

Symbol	X	Y	Z	Symbol	X	Y	Z
C	5.079900	-2.106400	0.760300	C	2.521200	2.123100	-3.296000
C	6.207700	-2.129200	-0.030900	C	2.689300	2.838500	-1.967700
H	7.227500	-2.021400	0.310800	C	2.164300	4.026600	-1.523900
C	5.787900	-2.376600	-1.368700	H	1.458500	4.645700	-2.056700
H	6.429900	-2.493600	-2.230200	C	2.719000	4.300900	-0.242900
C	4.416400	-2.496800	-1.357100	H	2.522200	5.173700	0.362500
C	3.428500	-2.773200	-2.470200	C	3.575700	3.276000	0.076500
C	2.577600	-1.545500	-2.718300	C	4.494500	3.086400	1.268900
C	1.235900	-1.390500	-2.958900	C	4.205900	1.784900	1.991000
H	0.497800	-2.179600	-2.961000	C	3.370800	1.501200	3.046900
C	0.994500	-0.010900	-3.211800	H	2.757000	2.215300	3.577500
H	0.036500	0.439900	-3.428400	C	3.457200	0.105600	3.314400
C	2.193000	0.654200	-3.121700	H	2.928900	-0.432900	4.088300

C	4.343600	-0.438300	2.416000
C	4.908800	-1.833200	2.239500
C	2.523600	-3.957600	-2.090300
H	1.921600	-3.735700	-1.205100
H	3.134800	-4.840700	-1.884200
H	1.844800	-4.198200	-2.914400
C	1.375100	2.792000	-4.066500
H	1.211900	2.279300	-5.017700
H	1.617300	3.837200	-4.276100
H	0.447000	2.766700	-3.489300
C	3.820900	2.276700	-4.115600
H	4.690300	1.861500	-3.594300
H	4.028900	3.336200	-4.288500
H	3.729400	1.772600	-5.082600
C	4.291700	4.250000	2.248100
H	4.532600	5.200800	1.765100
H	4.942600	4.125600	3.117000
H	3.258300	4.301700	2.599500
C	3.964000	-2.861800	2.881400
H	2.956000	-2.799500	2.464500
H	3.896400	-2.690900	3.960500
H	4.349000	-3.873100	2.722700
C	6.278200	-1.913900	2.939200
H	6.717600	-2.908100	2.813600
H	6.159500	-1.715200	4.007600
H	6.979900	-1.176200	2.539200
N	4.001400	-2.318000	-0.061200
N	3.146100	-0.294800	-2.812200
N	3.552400	2.395100	-0.986100
N	4.780500	0.591000	1.616300
H	5.480200	0.455900	0.900500
H	4.142600	-0.145300	-2.756600
H	3.022400	-2.320400	0.244900
H	3.954300	1.469600	-0.966000
C	4.191600	-3.128900	-3.759200
H	4.842100	-2.311200	-4.083800
H	3.480900	-3.329700	-4.565200
H	4.811500	-4.017900	-3.608700
C	5.959500	3.116400	0.785200
H	6.174800	4.081500	0.318900
H	6.167600	2.348200	0.032600
H	6.647900	2.970200	1.623200
C	1.235600	0.065000	0.532200
C	-0.592800	-1.206400	-0.132100
N	0.649700	-1.149400	0.401700
O	1.250000	-2.227600	0.777100
H	2.210400	0.073600	0.997400
H	-1.027200	-2.193500	-0.218500
C	-1.235600	-0.065200	-0.532700
C	0.592800	1.206200	0.131600
N	-0.649700	1.149200	-0.402200
O	-1.250100	2.227400	-0.777500
H	-2.210300	-0.073800	-0.997900
H	1.027200	2.193200	0.218000
C	-5.079800	2.106500	-0.760500
C	-6.207700	2.129600	0.030600
H	-7.227500	2.022000	-0.311300
C	-5.787900	2.377000	1.368500
H	-6.430100	2.494000	2.229900
C	-4.416400	2.496700	1.356900
C	-3.428600	2.773100	2.470100
C	-2.577500	1.545500	2.718100
C	-1.235800	1.390700	2.958600
H	-0.497800	2.180000	2.960500
C	-0.994100	0.011300	3.211500
H	-0.036000	-0.439400	3.428000
C	-2.192400	-0.654200	3.121600
C	-2.520200	-2.123100	3.295900
C	-2.689000	-2.838500	1.967700
C	-2.164400	-4.026700	1.523900
H	-1.458600	-4.646000	2.056500
C	-2.719600	-4.301100	0.243000
H	-2.523300	-5.173900	-0.362200
C	-3.576200	-3.276000	-0.076200
C	-4.495300	-3.086300	-1.268300
C	-4.206500	-1.785000	-1.990600
C	-3.371400	-1.501700	-3.046600
H	-2.757800	-2.215900	-3.577200
C	-3.457500	-0.106100	-3.314300
H	-2.929200	0.432200	-4.088400
C	-4.343700	0.438200	-2.416000
C	-4.908700	1.833300	-2.239700
C	-2.523900	3.957700	2.090400
H	-1.921900	3.736000	1.205100
H	-3.135200	4.840700	1.884400
H	-1.845000	4.198300	2.914500
C	-1.373600	-2.791700	4.065800
H	-1.210000	-2.279100	5.017000
H	-1.615600	-3.837000	4.275600
H	-0.445900	-2.766400	3.488000
C	-3.819500	-2.276900	4.116200
H	-4.689300	-1.861800	3.595400
H	-4.027300	-3.336400	4.289200
H	-3.727600	-1.772800	5.083200
C	-4.293200	-4.250000	-2.247400
H	-4.534200	-5.200700	-1.764100
H	-4.944400	-4.125600	-3.116000
H	-3.260000	-4.302100	-2.599100
C	-3.963600	2.861600	-2.881700
H	-2.955600	2.799100	-2.464700
H	-3.896000	2.690600	-3.960700
H	-4.348400	3.872900	-2.723000
C	-6.278000	1.914100	-2.939500
H	-6.717200	2.908400	-2.814000
H	-6.159200	1.715300	-4.007900
H	-6.979900	1.176500	-2.539500
N	-4.001400	2.317900	0.061100
N	-3.145800	0.294700	2.812100
N	-3.552400	-2.395000	0.986400
N	-4.780800	-0.590900	-1.616000
H	-5.480400	-0.455500	-0.900200
H	-4.142300	0.145000	2.757100
H	-3.022300	2.320200	-0.244900
H	-3.953800	-1.469300	0.966200
C	-4.191800	3.128600	3.759200
H	-4.811800	4.017500	3.608700
H	-3.481200	3.329300	4.565200
H	-4.842300	2.310700	4.083600
C	-5.960200	-3.115700	-0.784000
H	-6.167800	-2.347300	-0.031500
H	-6.175700	-4.080700	-0.317600
H	-6.648800	-2.969300	-1.621900

5. Structural information derived from single-crystal diffraction analyses

Table S1 Crystal data and structure refinements of **1**$_2$·**PZDO**, **2**$_2$·**PZDO** and **3**$_2$·**PZDO**

Crystal Data	**1**$_2$·**PZDO**	**2**$_2$·**PZDO**	**3**$_2$·**PZDO**
CCDC number	1976059	1976060	1976062
Crystal size, mm^3	0.350×0.100×0.050	0.290×0.150×0.130	Not Available
Empirical formula	$C_{82}H_{88}N_{14}O_{12}$	$C_{82}H_{92}N_{10}O_8$	$C_{78}H_{92}N_{14}O_6$
Formula wt	1461.66	1345.65	1321.65
T, K	100(2)	100(2)	100(2)
Crystal system	Triclinic	Trigonal	Monoclinic
Space group	P-1	R -3	C 2/c
a, Å	10.5727(5)	43.6284(11)	12.761(3)
b, Å	12.9980(5)	43.6284(11)	17.133(3)
c, Å	15.4783(8)	10.7942(3)	31.684(5)
α, deg	104.398(2)	90	90
β, deg	102.401(2)	90	93.976(10)
γ, deg	109.792(2)	120	90
V, Å^3	1831.31(15)	17793.4(10)	6911(2)
Z	1	9	4
Density, g/cm^3	1.325	1.130	1.270
μ, mm^{-1}	0.091	0.074	0.082
θ range, deg	3.048–27.529	2.173–25.349	1.993–25.493
No. of reflns collected	34417	7231	63040
No. of independent reflns	8391	7231	6385
R(int)	0.0506	?	0.1768
GOF	1.065	1.035	1.045
$R1[I > 2\sigma(I)]$	0.0474	0.0706	0.0644
$wR2[I > 2\sigma(I)]$	0.0999	0.1611	0.1483
$R1$ (all data)	0.0690	0.0979	0.1191
$wR2$ (all data)	0.1102	0.1751	0.1703

(See the next page)

(Continued)

Table S1 Crystal data and structure refinements of **4_2·PZDO**, **5·PZDO** and **PZDO**

Crystal Data	**4_2·PZDO**	**5·PZDO**	**PZDO**
CCDC number	1976064	1976063	1976061
Crystal size, mm^3	0.210×0.130×0.094	Not Available	0.430×0.160×0.070
Empirical formula	$C_{86}H_{88}N_{14}O_4$	$C_{53}H_{52}N_6O_7$	$C_4H_4N_2O_2$
Formula wt	1381.70	885.00	112.09
T, K	100(2)	100(2)	100(2)
Crystal system	Monoclinic	Monoclinic	Orthorhombic
Space group	P 21/n	P 21/c	P n m a
a, Å	16.7110(7)	10.9587(2)	11.866(3)
b, Å	12.6971(5)	59.0761(10)	11.883(3)
c, Å	18.2586(7)	22.0090(4)	6.3933(15)
α, deg	90	90	90
β, deg	99.327(4)	101.952(2)	90
γ, deg	90	90	90
V, $Å^3$	3822.9(3)	13939.7(4)	901.4(4)
Z	2	12	8
Density, g/cm^3	1.200	1.265	1.652
μ, mm^{-1}	0.076	0.198	0.136
θ range, deg	1.804–25.349	1.609–26.390	3.429–28.332
No. of reflns collected	45512	21770	6876
No. of independent reflns	7000	8216	1170
R(int)	0.0864	0.0455	0.0699
GOF	1.170	1.047	1.041
$R1[I > 2\sigma(I)]$	0.0982	0.0630	0.0448
$wR2[I > 2\sigma(I)]$	0.2234	0.1718	0.0951
$R1$ (all data)	0.1226	0.0743	0.0860
$wR2$ (all data)	0.2381	0.1813	0.1108

Table S2 Bond lengths corresponding to various non-covalent interactions inferred from the crystal structures

	**1**$_2$·**PZDO**	**2**$_2$·**PZDO**	**3**$_2$·**PZDO**	**4**$_2$·**PZDO**	**5**·**PZDO**
Hydrogen bonding between receptor and **PZDO**	2.79 Å	2.98 Å	2.79 Å	2.91 Å	2.97 Å
Hydrogen bonding between receptor and solvent	3.08 Å	3.08 Å	3.02 Å (w/ MeOH)<br>2.91 Å (w/ H_2O)	2.91 Å	N/A
C–H···π Interactions	2.41 Å	2.59 Å	2.40 Å	2.25 Å	2.44 Å
O–H···π Interactions	2.43 Å	2.32 Å	2.56 Å	2.62 Å	N/A
Donor–acceptor π–π interactions	3.56 Å	4.06 Å	3.84 Å	3.49 Å	3.94 Å

Note:

1. In the case that multiple bonds under one particular type of interaction were found in the crystal structures, the average bond lengths were calculated and reported.
2. The centroids of the aromatic rings were used to determine the bond lengths of C–H···π, O–H···π and donor–acceptor π–π interactions.
3. N/A (*i.e.* not available) indicates the absence of a particular type of non-covalent interactions.

6. X-ray experimental[S10–S17]

X-ray experimental for **1₂·PZDO**:

Single crystals of **1₂·PZDO** were obtained as clusters of colourless prisms by vapour diffusion of *n*-heptane into a chloroform/methanol solution containing a mixture of receptor **1** and **PZDO**. The data crystal was cut from a larger crystal and had approximate dimensions of 0.35 × 0.10 × 0.05 mm. The data were collected on a Rigaku AFC12 diffractometer with a Saturn 724+ CCD using graphite monochromator with MoKα radiation source (λ = 0.71073 Å). A total of 1616 frames of data were collected using ω-scans with a scan range of 0.5° and a counting time of 37 seconds per frame. The data were collected at 100 K using a Rigaku XStream low temperature device. Details of crystal data, data collection and structure refinement are listed in Table S1. Data reduction were performed using the Rigaku Americas Corporation's CrystalClear version 1.40. The structure was solved by direct methods using SIR2004 and refined by full-matrix least-squares on F^2 with anisotropic displacement parameters for the non-H atoms using SHELXL-2016/6. Structural analysis was aided by use of the programs PLATON and WinGX. The hydrogen atoms on carbon were calculated in ideal positions with isotropic displacement parameters set to 1.2xUeq of the attached atom (1.5xUeq for methyl hydrogen atoms). The hydrogen atoms bound to the pyrrolic nitrogen and the methanol oxygen atoms were located in a ΔF map and refined with isotropic displacement parameters.

The function, $\Sigma w(|F_o|^2 - |F_c|^2)^2$, was minimized, where $w = 1/[(\sigma(F_o))^2 + (0.1285\times P)^2]$ and $P = (|F_o|^2 + 2|F_c|^2)/3$. The data were checked for secondary extinction effects and no correction was necessary. Neutral atom scattering factors and values used to calculate the linear absorption coefficient are from the International Tables for X-ray Crystallography (1992). Tables of positional and thermal parameters, bond lengths and angles, and torsion angles are in the CIF file. CCDC deposition number: 1976059.

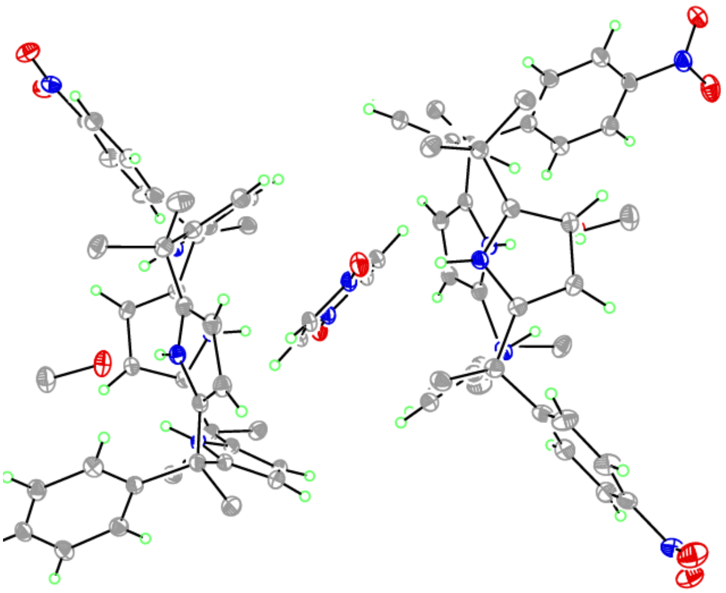

Fig. S37 View of **1₂·PZDO** showing the atom labelling scheme. Displacement ellipsoids are scaled to the 50% probability level. The methyl group hydrogen atoms have been removed for clarity.

X-ray experimental for **2₂·PZDO**:

Single crystals of **2_2·PZDO** were obtained as clusters of yellowish prisms by vapour diffusion of *n*-heptane into a chloroform/methanol solution containing a mixture of receptor **2** and **PZDO**. The data crystal was cut from a larger crystal and had approximate dimensions of 0.29 × 0.15 × 0.13 mm. The data were collected on a Rigaku AFC12 diffractometer with a Saturn 724+ CCD using a graphite monochromator with MoKα radiation (λ = 0.71073 Å). A total of 901 frames of data were collected using ω-scans with a scan range of 0.5° and a counting time of 45 seconds per frame. The data were collected at 100 K using a Rigaku XStream Cryostream low temperature device. Details of crystal data, data collection and structure refinement are listed in Table S1. Data collection was performed using the Rigaku Americas Corporation's CrystalClear version 1.40. Unit cell refinement and data reduction were performed using Rigaku Oxford Diffraction's CrysAlisPro V 1.171.40.53. The structure was solved by direct methods using SHELXT and refined by full-matrix least-squares on F^2 with anisotropic displacement parameters for the non-H atoms using SHELXL-2016/6. Structure analysis was aided by use of the programs PLATON, OLEX2 and WinGX. The hydrogen atoms on carbon were calculated in ideal positions with isotropic displacement parameters set to 1.2xUeq of the attached atom (1.5xUeq for methyl hydrogen atoms).

Some disordered solvent molecules centred near 0, 0, ½ could not be adequately modelled. The contributions to the scattering factors due to this solvent molecule were removed by use of the utility SQUEEZE in PLATON. PLATON was used as incorporated in WinGX. The crystal was also twinned. The twin law was determined using PLATON.

The function, $\Sigma w(|F_o|^2 - |F_c|^2)^2$, was minimized, where $w = 1/[(\sigma(F_o))^2 + (0.1285\times P)^2]$ and $P = (|F_o|^2 + 2|F_c|^2)/3$. The data were checked for secondary extinction effects and no correction was necessary. Neutral atom scattering factors and values used to calculate the linear absorption coefficient are from the International Tables for X-ray Crystallography (1992). Tables of positional and thermal parameters, bond lengths and angles, and torsion angles are in the CIF file. CCDC deposition number: 1976060.

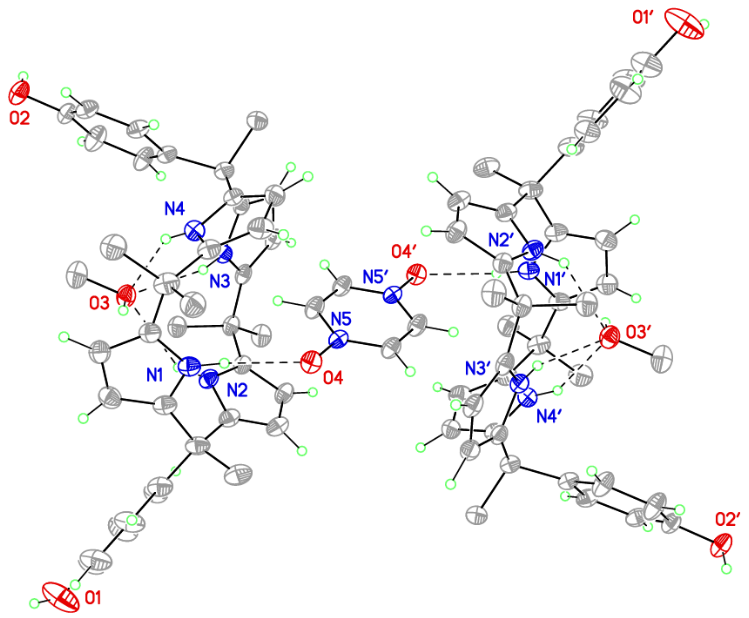

Fig. S38 View of **2_2·PZDO** showing the atom labelling scheme. Displacement ellipsoids are scaled to the 50% probability level. The methyl group hydrogen atoms have been removed for clarity.

X-ray experimental for **3_2·PZDO**:

Single crystals of **3_2·PZDO** were obtained as yellow prisms by vapour diffusion of *n*-heptane into a chloroform/methanol solution containing a mixture of receptor **3** and **PZDO**. The data crystal had approximate dimensions of 0.21 × 0.13 × 0.094 mm. The data were collected at -173 °C on a Nonius Kappa CCD diffractometer using a Bruker AXS Apex II detector and a graphite monochromator with MoKα radiation (λ = 0.71073 Å). Reduced temperatures were maintained by use of an Oxford Cryosystems 700 low-temperature device. A total of 1767 frames of data were collected using ω- and ϕ-scans with a scan range of 0.6° and a counting time of 67 seconds per frame. Details of crystal data, data collection and structure refinement are listed in Table S1. Data reduction were performed using Bruker AXS, Inc's SAINT V827B. The structure was solved by direct methods using SHELXT and refined by full-matrix least-squares on F^2 with anisotropic displacement parameters for the non-H atoms using SHELXL-2016/6. Structure analysis was aided by use of the programs PLATON and WinGX. The hydrogen atoms bound to carbon atoms were calculated in idealised positions. The hydrogen atoms on the pyrrole nitrogen atoms and the oxygen atoms were observed in a Δ*F* map and refined with isotropic displacement parameters.

The function, $\Sigma w(|F_o|^2 - |F_c|^2)^2$, was minimized, where $w = 1/[(\sigma(F_o))^2 + (0.1285\times P)^2]$ and $P = (|F_o|^2 + 2|F_c|^2)/3$. The data were corrected for secondary extinction effects. The correction takes the form: $F_{corr} = kF_c/[1 + (4.2(2)\times10^{-6})\times F_c^2\lambda^3/(\sin 2\theta)]^{0.25}$, where *k* is the overall scale factor. Neutral atom scattering factors and values used to calculate the linear absorption coefficient are from the International Tables for X-ray Crystallography (1992). Tables of positional and thermal parameters, bond lengths and angles, and torsion angles are in the CIF file. CCDC deposition number: 1976062.

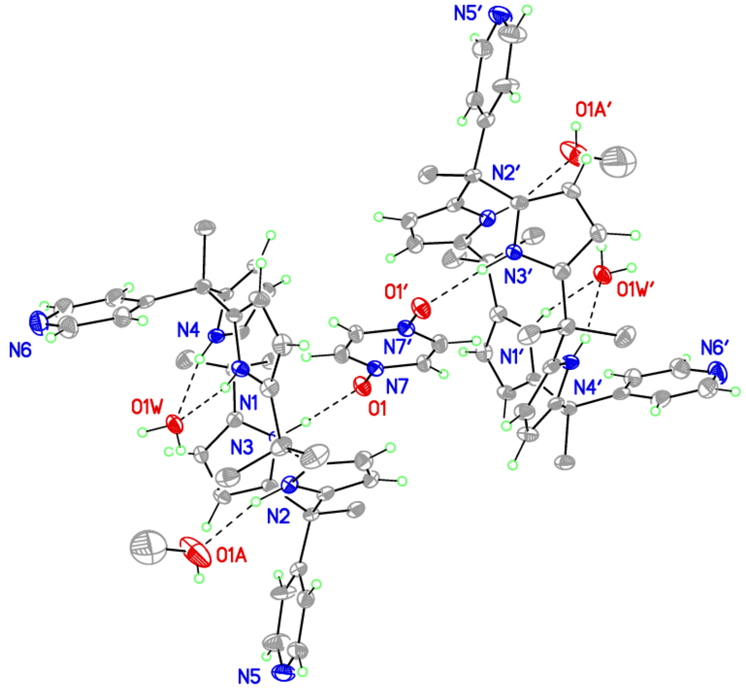

Fig. S39 View of **3_2·PZDO** showing the atom labelling scheme. Displacement ellipsoids are scaled to the 50% probability level. The methyl group hydrogen atoms have been removed for clarity.

X-ray experimental for **4_2·PZDO**:

Single crystals of **4_2·PZDO** were obtained as yellow prisms by vapour diffusion of *n*-heptane into a chloroform/methanol solution containing a mixture of receptor **4** and **PZDO**. The data crystal had approximate dimensions of 0.21 × 0.13 × 0.094 mm. The data were collected on a Rigaku AFC12 diffractometer with a Saturn 724+ CCD using a graphite monochromator with MoKα radiation (λ = 0.71073 Å). A total of 1368 frames of data were collected using ω-scans with a scan range of 0.5° and a counting time of 50 seconds per frame. The data were collected at 100 K using a Rigaku XStream Cryostream low temperature device. Details of crystal data, data collection and structure refinement are listed in Table S1. Data collection was performed using the Rigaku Americas Corporation's CrystalClear version 1.40. Unit cell refinement and data reduction were performed using Rigaku Oxford Diffraction's CrysAlisPro V 1.171.40.37a. The structure was solved by direct methods using SHELXT and refined by full-matrix least-squares on F^2 with anisotropic displacement parameters for the non-H atoms using SHELXL-2016/6. Structure analysis was aided by use of the programs PLATON and WinGX. The hydrogen atoms on carbon were calculated in ideal positions with isotropic displacement parameters set to 1.2xUeq of the attached atom (1.5xUeq for methyl hydrogen atoms).

The function, $\Sigma w(|F_o|^2 - |F_c|^2)^2$, was minimized, where $w = 1/[(\sigma(F_o))^2 + (0.1285 \times P)^2]$ and $P = (|F_o|^2 + 2|F_c|^2)/3$. The data were checked for secondary extinction effects and no correction was necessary. Neutral atom scattering factors and values used to calculate the linear absorption coefficient are from the International Tables for X-ray Crystallography (1992). Tables of positional and thermal parameters, bond lengths and angles, and torsion angles are in the CIF file. CCDC deposition number: 1976064.

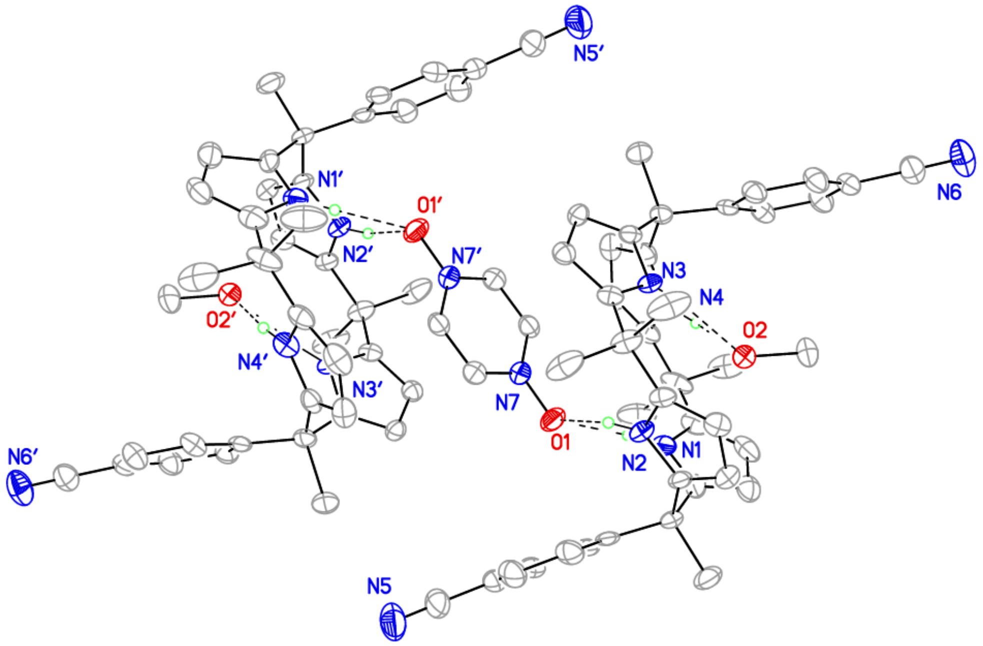

Fig. S40 View of **4_2·PZDO** showing the atom labelling scheme. Displacement ellipsoids are scaled to the 50% probability level. The methyl group hydrogen atoms have been removed for clarity.

X-ray experimental for **5·PZDO**:

Single crystals of **5·PZDO** were obtained as thin colourless needles by vapour diffusion of *n*-heptane into a chloroform/methanol solution containing a mixture of receptor **5** and **PZDO**. The data crystal was cut from a larger crystal and had an approximate maximum dimension of 0.1 mm. The data were collected on Beamline 5.0.3 at the Advanced Light Source at the Lawrence Berkeley National Laboratory. The synchrotron beamline produced a wavelength of 0.97741 Å. A total of 180 frames of data were collected using ϕ-scans with a scan range of 1° and a counting time of 0.5 seconds per frame for frames collected with a detector offset of 0.0°. The data were collected at 100 K using an Oxford Cryostream low temperature device. Details of crystal data, data collection and structure refinement are listed in Table S1. Data collection was performed using the Beamline Operating Software, BOS/B3. The unit cell refinement and data reduction were performed using Agilent Technologies CrysAlisPro V 1.171.40.37a. The structure was solved by direct methods using SHELXT and refined by full-matrix least-squares on F^2 with anisotropic displacement parameters for the non-H atoms using SHELXL-2016/6. Structure analysis was aided by use of the programs PLATON and WinGX. The hydrogen atoms were calculated in ideal positions with isotropic displacement parameters set to 1.2xUeq of the attached atom (1.5xUeq for methyl hydrogen atoms).

A solvent void existed in a channel along *x*, 1/6, ½. The solvent molecules could not be adequately modelled. The contribution to the scattering due to the solvent was removed using SQUEEZE as found in PLATON.

The function, $\Sigma w(|F_o|^2 - |F_c|^2)^2$, was minimized, where $w = 1/[(\sigma(F_o))^2 + (0.1285\times P)^2]$ and $P = (|F_o|^2 + 2|F_c|^2)/3$. The data were checked for secondary extinction effects and no correction was necessary. Neutral atom scattering factors and values used to calculate the linear absorption coefficient are from the International Tables for X-ray Crystallography (1992). Tables of positional and thermal parameters, bond lengths and angles, and torsion angles are in the CIF file. CCDC deposition number: 1976063.

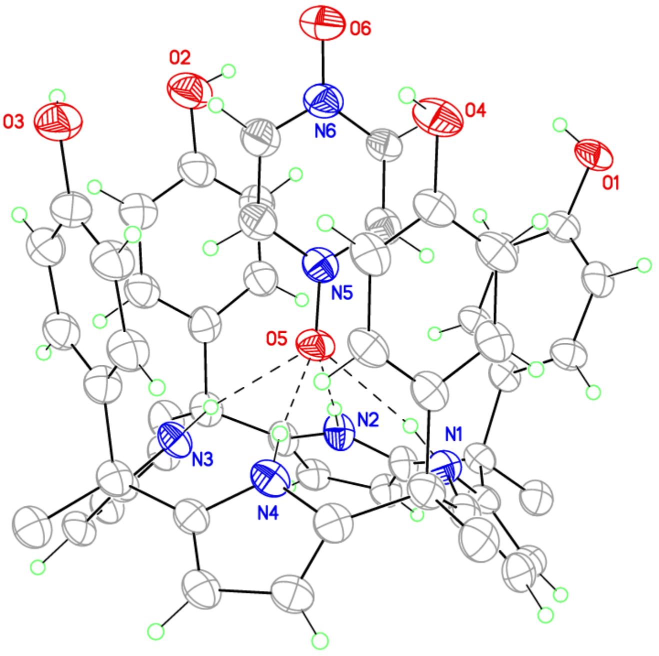

Fig. S41 View of **5·PZDO** showing the atom labelling scheme. Displacement ellipsoids are scaled to the 30% probability level. The methyl group hydrogen atoms have been removed for clarity.

X-ray experimental for **PZDO**:

Single crystals of **PZDO** were obtained as colourless needles by vapour diffusion of *n*-heptane into a chloroform/methanol solution containing **PZDO**. The data crystal had approximate dimensions of 0.43 × 0.16 × 0.07 mm. The data were collected at -173 °C on a Nonius Kappa CCD diffractometer using a Bruker AXS Apex II detector and a graphite monochromator with MoKα radiation (λ = 0.71073 Å). Reduced temperatures were maintained by use of an Oxford Cryosystems 700 low-temperature device. A total of 556 frames of data were collected using ω- and ϕ-scans with a scan range of 0.7° and a counting time of 29 seconds per frame. Details of crystal data, data collection and structure refinement are listed in Table S1. Data reduction were performed using Bruker AXS, Inc's SAINT V827B. The structure was solved by direct methods using SHELXT and refined by full-matrix least-squares on F^2 with anisotropic displacement parameters for the non-H atoms using SHELXL-2016/6. Structure analysis was aided by use of the programs PLATON and WinGX. The hydrogen atoms bound to carbon atoms were calculated in idealised positions. There are two crystallographically unique molecules in the asymmetric unit. One molecule resides on a crystallographic mirror plane of symmetry, whereas the other molecule resides around a crystallographic inversion centre.

The function, $\Sigma w(|F_o|^2 - |F_c|^2)^2$, was minimized, where $w = 1/[(\sigma(F_o))^2 + (0.1285\times P)^2]$ and $P = (|F_o|^2 + 2|F_c|^2)/3$. The data were checked for secondary extinction effects and no correction was necessary. Neutral atom scattering factors and values used to calculate the linear absorption coefficient are from the International Tables for X-ray Crystallography (1992). Tables of positional and thermal parameters, bond lengths and angles, and torsion angles are in the CIF file. CCDC deposition number: 1976061.

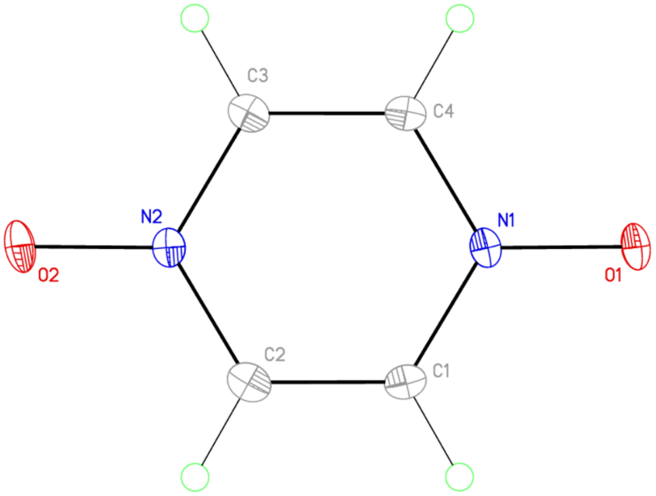

Fig. S42 View of **PZDO** showing the atom labelling scheme. Displacement ellipsoids are scaled to the 50% probability level.

7. ^{1}H NMR, ^{13}C NMR and HRMS spectra

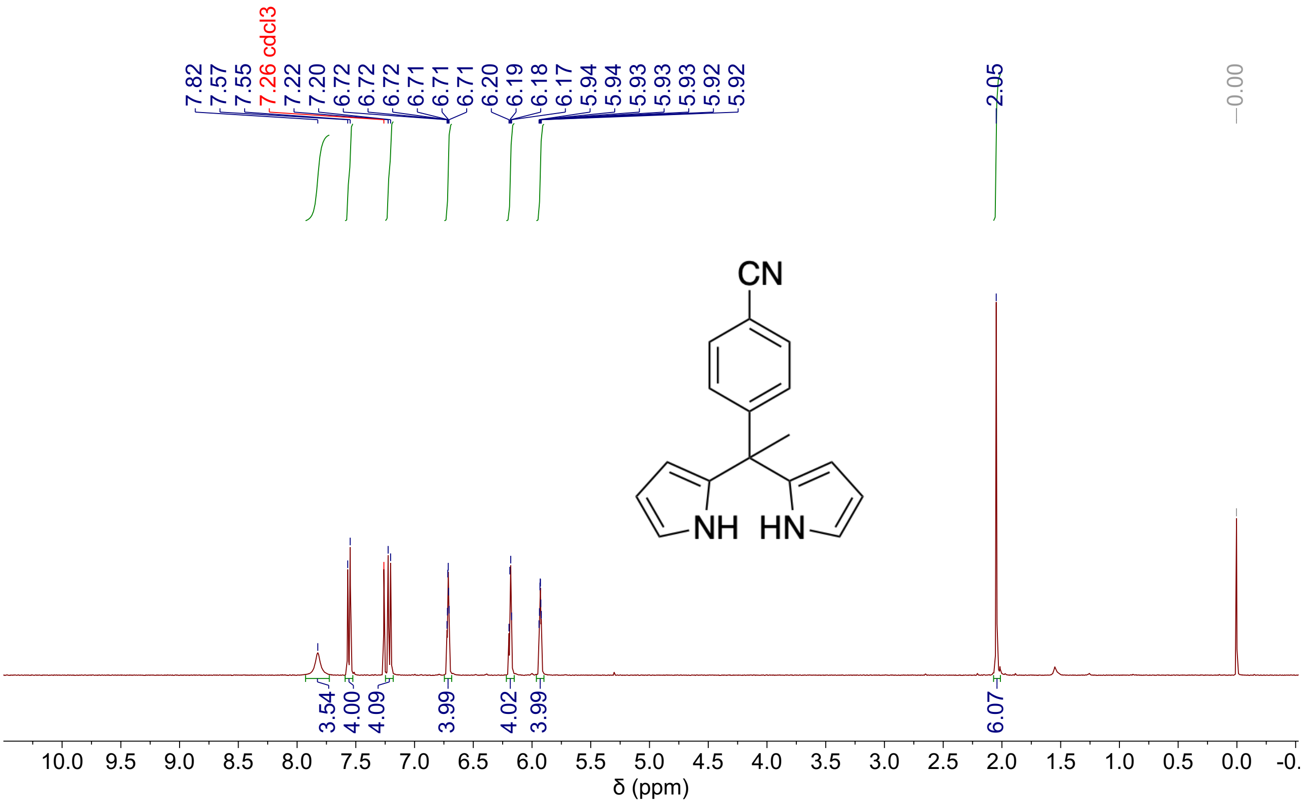

Fig. S43 ^{1}H NMR spectrum of **7** recorded in $CDCl_3$.

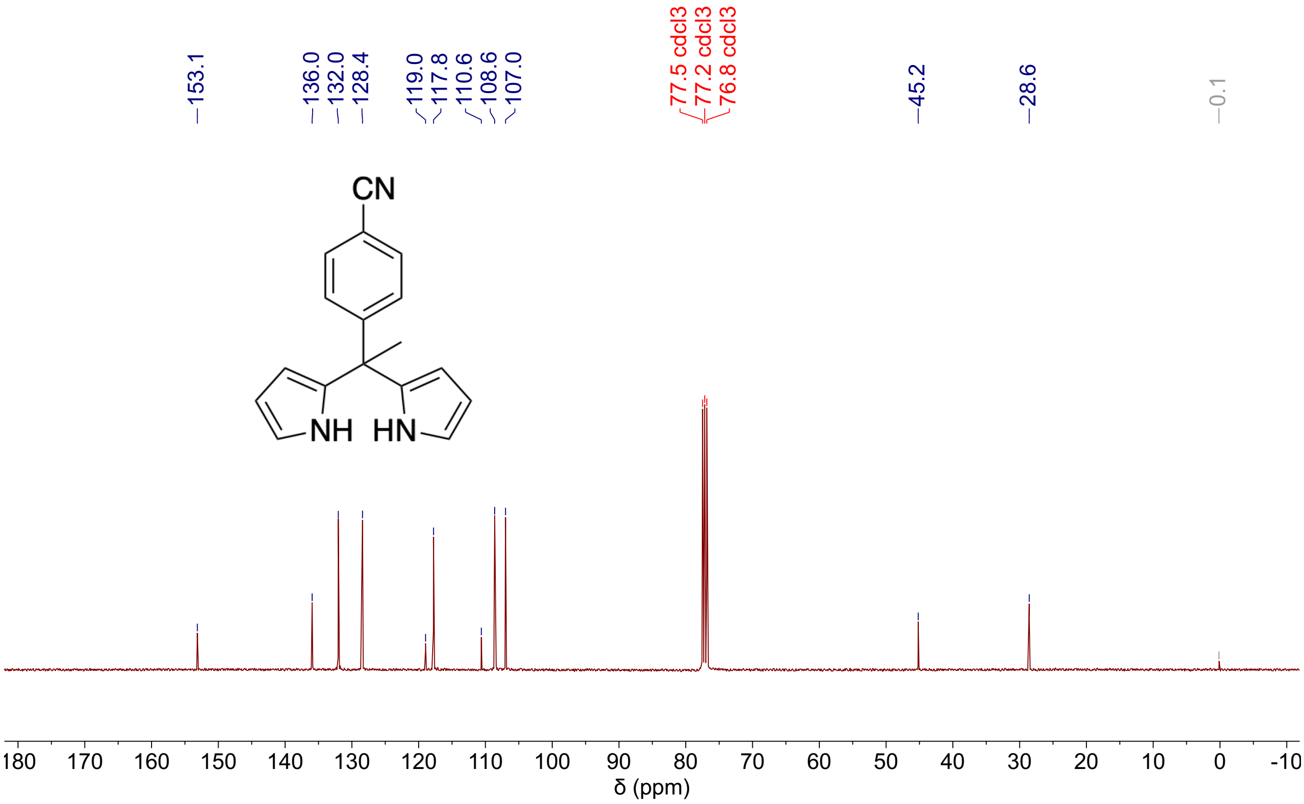

Fig. S44 ^{13}C NMR spectrum of **7** recorded in $CDCl_3$.

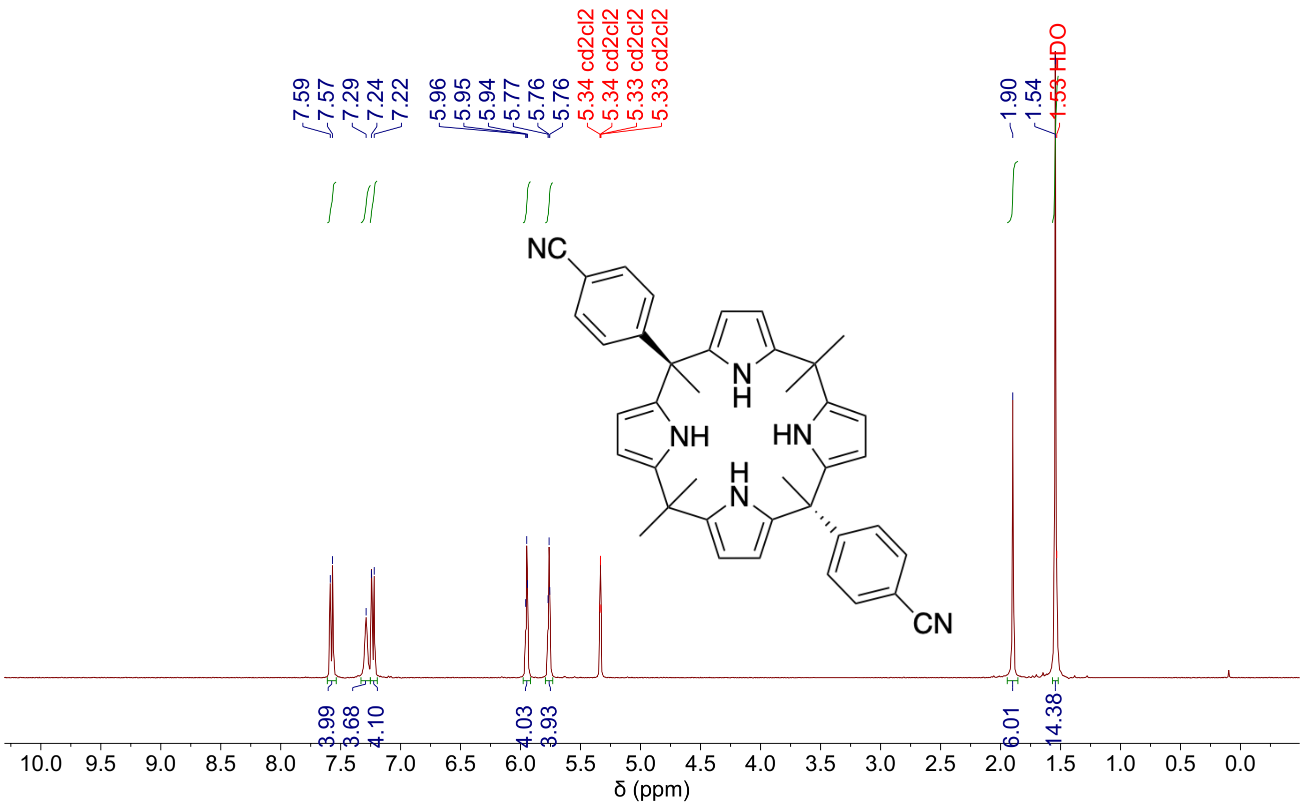

Fig. S45 ^{1}H NMR spectrum of **4** recorded in CD_2Cl_2. Note: Overlap of the peak at 1.54 ppm with the water peak at 1.53 ppm is thought to account for why the observed integral (*i.e.* 14.38) is slightly higher than the theoretical value (*i.e.* 12.00).

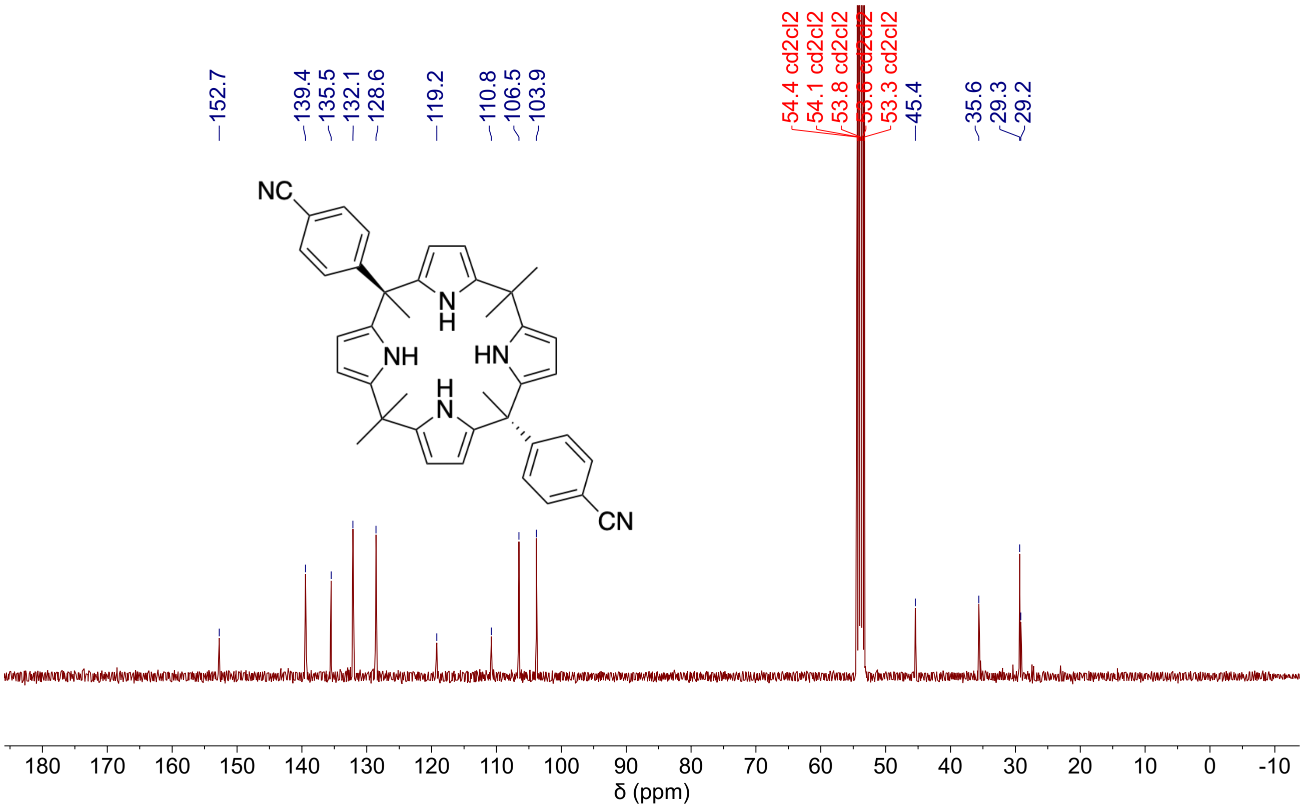

Fig. S46 ^{13}C NMR spectrum of **4** recorded in CD_2Cl_2.

Target Compound Screening Report

Results Acquired by The University of Texas at Austin Mass Spectrometry Facility

**Data File**	MSF20-0240(cg294)_hrESIpos1.d	**Sample Name**	0240(cg294)	**Comment**	0240(cg294)
**Position**	P1-B2	**Instrument Name**	Instrument 1	**User Name**	
**Acq Method**	FIA_pos.m	**Acquired Time**	2/21/2020 12:45:51 PM	**DA Method**	KS.m

MS Zoomed Spectrum

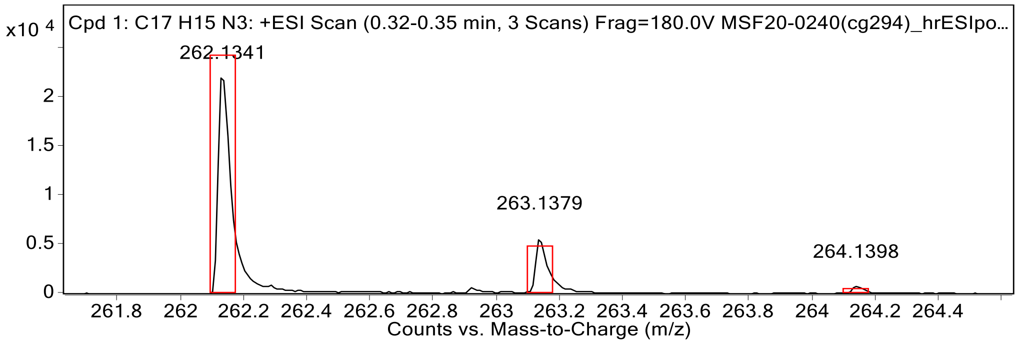

MS Spectrum Peak List

Obs. m/z	Calc. m/z	Charge	Abundance	Formula	Ion Species	Tgt Mass Error (ppm)
195.0919			156319			
262.1341	262.1339	1	22939	C17H15N3	(M+H)+	-0.89
263.1379	263.1369	1	5680	C17H15N3	(M+H)+	-3.61
264.1398	264.1399	1	760	C17H15N3	(M+H)+	0.33

--- End Of Report ---

Fig. S47 HR-ESI$^+$-MS reports for [**7**+H]$^+$.

Target Compound Screening Report

Results Acquired by The University of Texas at Austin Mass Spectrometry Facility

**Data File**	MSF20-0241(cg299_trans)_hrESIpos1.d	**Sample Name**	0241(cg299_trans)	**Comment**	0241(cg299_trans)
**Position**	P1-B3	**Instrument Name**	Instrument 1	**User Name**	
**Acq Method**	FIA_pos.m	**Acquired Time**	2/21/2020 12:51:59 PM	**DA Method**	KS.m

MS Zoomed Spectrum

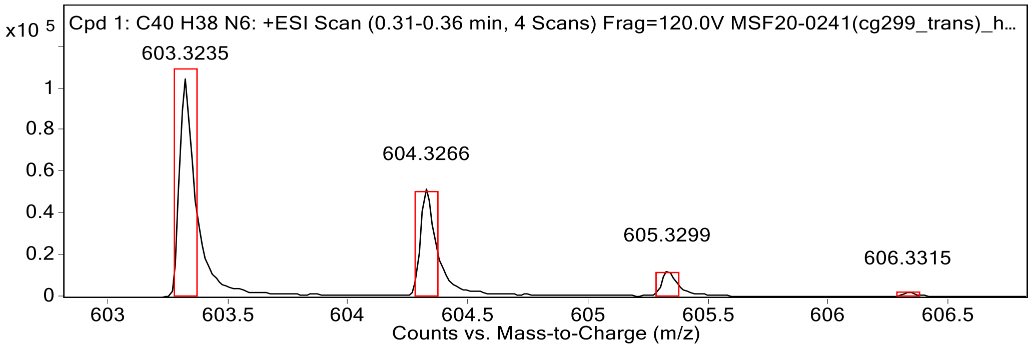

MS Spectrum Peak List

Obs. m/z	Calc. m/z	Charge	Abundance	Formula	Ion Species	Tgt Mass Error (ppm)
603.3235	603.3231	1	104845	C40H38N6	(M+H)+	-0.66
604.3266	604.3262	1	52098	C40H38N6	(M+H)+	-0.74
605.3299	605.3292	1	12988	C40H38N6	(M+H)+	-1.07
606.3315	606.3323	1	1970	C40H38N6	(M+H)+	1.29
607.3250	607.3354	1	508	C40H38N6	(M+H)+	17.04
625.3052			110436			

--- End Of Report ---

Fig. S48 HR-ESI$^+$-MS reports for [**4**+H]$^+$.

8. References

S1. Y. Shao, L. F. Molnar, Y. Jung, J. Kussmann, C. Ochsenfeld, S. T. Brown, A. T. B. Gilbert, L. V. Slipchenko, S. V. Levchenko, D. P. O'Neill, R. A. DiStasio Jr, R. C. Lochan, T. Wang, G. J. O. Beran, N. A. Besley, J. M. Herbert, C. Yeh Lin, T. Van Voorhis, S. Hung Chien, A. Sodt, R. P. Steele, V. A. Rassolov, P. E. Maslen, P. P. Korambath, R. D. Adamson, B. Austin, J. Baker, E. F. C. Byrd, H. Dachsel, R. J. Doerksen, A. Dreuw, B. D. Dunietz, A. D. Dutoi, T. R. Furlani, S. R. Gwaltney, A. Heyden, S. Hirata, C.-P. Hsu, G. Kedziora, R. Z. Khalliulin, P. Klunzinger, A. M. Lee, M. S. Lee, W. Liang, I. Lotan, N. Nair, B. Peters, E. I. Proynov, P. A. Pieniazek, Y. Min Rhee, J. Ritchie, E. Rosta, C. David Sherrill, A. C. Simmonett, J. E. Subotnik, H. Lee Woodcock Iii, W. Zhang, A. T. Bell, A. K. Chakraborty, D. M. Chipman, F. J. Keil, A. Warshel, W. J. Hehre, H. F. Schaefer Iii, J. Kong, A. I. Krylov, P. M. W. Gill and M. Head-Gordon, *Phys. Chem. Chem. Phys.*, 2006, **8**, 3172–3191.

S2. Gaussian 16, Revision C.01, M. J. Frisch, G. W. Trucks, H. B. Schlegel, G. E. Scuseria, M. A. Robb, J. R. Cheeseman, G. Scalmani, V. Barone, G. A. Petersson, H. Nakatsuji, X. Li, M. Caricato, A. V. Marenich, J. Bloino, B. G. Janesko, R. Gomperts, B. Mennucci, H. P. Hratchian, J. V. Ortiz, A. F. Izmaylov, J. L. Sonnenberg, D. Williams-Young, F. Ding, F. Lipparini, F. Egidi, J. Goings, B. Peng, A. Petrone, T. Henderson, D. Ranasinghe, V. G. Zakrzewski, J. Gao, N. Rega, G. Zheng, W. Liang, M. Hada, M. Ehara, K. Toyota, R. Fukuda, J. Hasegawa, M. Ishida, T. Nakajima, Y. Honda, O. Kitao, H. Nakai, T. Vreven, K. Throssell, J. A. Montgomery, Jr., J. E. Peralta, F. Ogliaro, M. J. Bearpark, J. J. Heyd, E. N. Brothers, K. N. Kudin, V. N. Staroverov, T. A. Keith, R. Kobayashi, J. Normand, K. Raghavachari, A. P. Rendell, J. C. Burant, S. S. Iyengar, J. Tomasi, M. Cossi, J. M. Millam, M. Klene, C. Adamo, R. Cammi, J. W. Ochterski, R. L. Martin, K. Morokuma, O. Farkas, J. B. Foresman, and D. J. Fox, Gaussian, Inc., Wallingford CT, 2016.

S3. P. Thordarson, *Chem. Soc. Rev.,* 2011, **40**, 1305–1323.

S4. G. Bruno, G. Cafeo, F. H. Kohnke and F. Nicolò, *Tetrahedron*, 2007, **63**, 10003–10010.

S5. L. Adriaenssens, G. Gil-Ramírez, A. Frontera, D. Quiñonero, E. C. Escudero-Adán and P. Ballester, *J. Am. Chem. Soc.*, 2014, **136**, 3208–3218.

S6. D. S. Kim, V. M. Lynch, J. S. Park and J. L. Sessler, *J. Am. Chem. Soc.*, 2013, **135**, 14889–14894.

S7. P. Anzenbacher, K. Jursíková, V. M. Lynch, P. A. Gale and J. L. Sessler, *J. Am. Chem. Soc.*, 1999, **121**, 11020–11021.

S8. A. Baeyer, *Ber. Dtsch. Chem. Ges.*, 1886, **19**, 2184–2185.

S9. N. J. Babu and A. Nangia, *CrystEngComm*, 2007, **9**, 980–983.

S10. CrysAlisPro V 1.171.40 (2019). Rigaku Oxford Diffraction, Tokyo, Japan.

S11. G. M. Sheldrick, *Acta Cryst. A*, 2015, **71**, 3–8.

S12. G. M. Sheldrick, *Acta Cryst. C*, 2015, **71**, 3–8.

S13. O. V. Dolomanov, L. J. Bourhis, R. J. Gildea, J. A. K. Howard and H. *J.* Puschmann *Appl. Cryst.*, 2009, **42**, 339–341.

S14. A. L. Spek, *Acta Cryst. D*, 2009, **65**, 148–155.

S15. A. L. Spek, *Acta Cryst. C*, 2015, **71**, 9–18.

S16. L. J. Farrugia, *J. Appl. Cryst.*, 1999, **32**, 837–838.

S17. A. J. C. Wilson (editor), International Tables for X-ray Crystallography. Vol. C*, Tables 4.2.6.8 and 6.1.1.4*, Kluwer Academic Press, 1992.
